# A Bayesian Approach Towards Missing Covariate Data in Multilevel Latent Regression Models

**DOI:** 10.1007/s11336-022-09888-0

**Published:** 2022-11-23

**Authors:** Christian Aßmann, Jean-Christoph Gaasch, Doris Stingl

**Affiliations:** 1 Leibniz Institute for Educational Trajectories Bamberg, Bamberg, Germany; 2 Otto-Friedrich-Universität Bamberg, Bamberg, Germany; 3 Otto-Friedrich-Universität Bamberg, Bamberg, Germany

**Keywords:** Item response theory, population heterogeneity, Markov chain Monte Carlo, classification and regression trees, missing values

## Abstract

The measurement of latent traits and investigation of relations between these and a potentially large set of explaining variables is typical in psychology, economics, and the social sciences. Corresponding analysis often relies on surveyed data from large-scale studies involving hierarchical structures and missing values in the set of considered covariates. This paper proposes a Bayesian estimation approach based on the device of data augmentation that addresses the handling of missing values in multilevel latent regression models. Population heterogeneity is modeled via multiple groups enriched with random intercepts. Bayesian estimation is implemented in terms of a Markov chain Monte Carlo sampling approach. To handle missing values, the sampling scheme is augmented to incorporate sampling from the full conditional distributions of missing values. We suggest to model the full conditional distributions of missing values in terms of non-parametric classification and regression trees. This offers the possibility to consider information from latent quantities functioning as sufficient statistics. A simulation study reveals that this Bayesian approach provides valid inference and outperforms complete cases analysis and multiple imputation in terms of statistical efficiency and computation time involved. An empirical illustration using data on mathematical competencies demonstrates the usefulness of the suggested approach.

**Supplementary Information:**

The online version contains supplementary material available at 10.1007/s11336-022-09888-0.

## Introduction

Models for measurement and structural analysis of latent traits have been developed among others by Muthén (1979), Zwinderman (1991) and Adams et al. (1997). These latent regression models (LRM) typically use a regression equation to assess the relationship between the latent trait and additional covariates and link measurements to the latent trait via a model, possibly arising from the context of item response theory (IRT; e.g., Embretson & Reise, 2000). As demonstrated by Rijmen et al. (2003), and described extensively in Wilson and De Boeck (2004), these models can be conceptualized within the wider context of nonlinear mixed models. Since the derived likelihood functions involve multiple integrals arising from the involved latent variables, a Bayesian framework using Markov chain Monte Carlo (MCMC) techniques is eminently suited to provide inference, see e.g. Edwards (2010). The seminal article of Albert (1992) adopts data augmentation (DA), see Tanner and Wong (1987), within a Bayesian estimation approach for measurement models with dichotomous items.^1^ Further work adopted Albert’s DA procedure for extended model structures incorporating multilevel and clustered data structures (Aßmann & Boysen-Hogrefe, 2011; Fox, 2005; Fox & Glas, 2001; Johnson & Jenkins, 2005). Prominent applications of these models arise in the context of large-scale assessment studies like the Programme for International Student Assessment (PISA; e.g., OECD, 2014), the Trends in International Mathematics and Science Study (TIMSS; e.g., Mullis & Martin, 2013), the Programme for the International Assessment of Adult Competencies (PIAAC; e.g., OECD, 2013b) or the National Assessment of Educational Progress (NAEP; e.g., Allen et al., 1999).

However, surveyed information is often seriously afflicted by item nonresponse. Si and Reiter (2013), for example, report less than five percent complete cases on a set of 80 background variables in a data file of the Trends in International Mathematics and Science Study (TIMSS; e.g., Mullis & Martin, 2013). Especially in multilevel contexts, such a large fraction of missing values poses a challenge to efficient parameter estimation. An appropriate strategy for handling missing values and corresponding model specification is required when analyzing the data. While several studies deal with the impact of missing or omitted competence items (Köhler et al., 2015; Pohl et al., 2014), there has been less work on missing values in background variables. By default, the educational assessment studies cited above treat missing values in context questionnaires via dummy variable adjustments, see e.g. OECD (2014). Aside from the obvious information loss, dummy-variable adjustments for missing values can cause biased estimation, see Jones (1996). The involved categorization of information may have negative side effects on the assumed functional relationship, see also Grund et al. (2020) for a more detailed discussion. These results are in line with a recent study by Rutkowski (2011) who found non negligible bias and misleading interpretations at the population level when partially missing covariates are dummy coded.^2^

With the latent factor being of substantial interest, the Bayesian approach implemented in terms of a MCMC algorithm using the DA device has the advantage to provide direct access to the latent factors in terms of the posterior distribution.^3^ Furthermore, in the presence of missing values in background variables, DA in the Bayesian context offers a conceptually straightforward way to deal with missing values. The vector of unknown quantities can be augmented with the missing values in covariates. Correspondingly, the MCMC sampling scheme incorporates the set of full conditional distributions of the missing values. This approach has the advantage that the modeling of the full conditional distributions can incorporate information available in form of a latent variable serving in the considered model context as a sufficient statistic.^4^ These advantages result in increased statistical efficiency and reduced computational costs as illustrated in this paper. Such a handling of information is in principle also possible in the context of Maximum Likelihood estimation in terms of a chained equation approach via iteratively sampling from an assumed or approximated set of full conditional distributions, see Grund et al. (2020) for a discussion in the absence of hierarchical structures. In addition, in data contexts with a large number of covariates relative to the number of observations, the Bayesian approach incorporates shrinkage in terms of the involved prior distributions and facilitates updating of information with regard to the modeled relationships. Next, Bayesian estimators of parameters or functions thereof, like context effects and uncertainty measures, are directly accessible without the use of combining rules.

The DA principle has been successfully applied in different contexts ranging from multivariate panel models to social network analysis and educational large-scale assessments by Liu et al. (2000), Koskinen et al. (2010), Blackwell et al. (2017) and Kaplan and Su (2018). Full conditional distributions of missing values are typically operationalized in terms of a parametric modeling approach as discussed by Grund et al. (2020) and Erler et al. (2016). Goldstein et al. (2014), Erler et al. (2016) and Grund et al. (2018) provide a discussion in the context of linear regression models for metrically scaled hierarchical data.

In this article, we extend the DA approach towards missing values in covariate data in extended hierarchical structures in LRMs for dependent variables with binary and ordinal scale.^5^ We also illustrate that DA allows for direct access to a valid model specification for the missing values incorporating information available in form of sufficient statistics as suggested by the Hammersley–Clifford theorem, see Robert and Casella (2004). Further, specifying the full conditional distributions of missing values in terms of sufficient statistics arising in the hierarchical latent regression context has the potential to reduce the computational burden. The role of sufficient statistics has also been stressed by Neal and Kypraios (2015) discussing situations, where the augmented variables and sufficient statistics are discrete and the models of interest belong to well known probability distributions. Our approach extends on this as we consider hierarchical structures and identifying restrictions arising from the factor like model structures resulting in complex posterior distributions.^6^ Consideration of full conditional distributions for handling of missing values enriched with information from latent model structures extends also the sequential imputations approach discussed by Kong et al. (1994). Whereas the sequential imputations approach builds on predictive distributions for missing values separating thereby the model for the missing values in the covariate variables from the considered latent model structures, our approach is based on smoothed, i.e. full conditional distributions incorporating information from the latent model structures via the DA principle.^7^

In combination with modeling the full conditional distributions of missing values via non-parametric sequential regression trees as suggested by Burgette and Reiter (2010) and Doove et al. (2014), the DA approach suggested in this paper offers high flexibility in empirical applications to cope with nonlinear relationships, e.g. interaction terms, within a potentially large set of covariates having different scales. The proposed modeling approach allows hence for tackling research questions typically addressed in sociology, psychology, and economics in the field of educational inequality and the role of institutions, see among others Carlsson et al. (2015), Passaretta and Skopek (2021) and Cornelissen and Dustmann (2019). It simultaneously addresses the uncertainty associated with the estimation of a latent trait variable and the imputation of missing values in manifest covariate variables. The reciprocal dependence of outcomes and predictors is reflected to the full extent by the Bayesian DA estimation algorithm. The benefits of the suggested fully Bayesian approach arise in terms of methodological stringency and gains in statistical efficiency. Illustration of the suggested approach is provided by means of a simulation study and an empirical application using the first wave of the starting cohort of ninth graders surveyed in the German National Educational Panel Study—Educational Trajectories in Germany (NEPS), see Blossfeld and Roßbach (2019). To highlight the benefits of considering sufficient statistics within the suggested DA approach towards missing values in covariates, we provide a comparison with a classical imputation setup, where the full conditional distributions of missing values are defined on the basis of directly observable quantities only, see e.g. von Hippel (2007). As shown in the simulations, the consideration of sufficient statistics accelerates the computation up to a third and ensure the feasibility of specifying full conditional distributions in multilevel contexts.

The paper proceeds as follows. Section 2 outlines the specification of the considered model setup and provides the corresponding Bayesian sampling algorithm that deals with structures reflecting heterogeneity and missing values in covariates via DA. Performance of the estimation routine is demonstrated through a simulation study in Sect. 3, whilst Sect. 4 provides the empirical illustration using data from the NEPS. Section 5 concludes.

## Model Setup and Bayesian Inference

### Model Setup

Consider *J* measurement items observed on *N* individuals summarized in a $N\times J$ data matrix $Y=(y_1,\ldots ,y_N)'$ with row vectors $y_i=(y_{i1},\ldots , y_{ij},\ldots ,y_{iJ})$ for each $i=1,\ldots ,N$ and $j=1,\ldots ,J$. In case of binary measurements $y_{ij}$ denotes a random variable taking the value $y_{ij}=1$ if in an educational assessment context respondent *i* is able to solve item *j* and the value $y_{ij}=0$ otherwise. To analyze this kind of test items, Lord (1952, 1953) proposes an IRT model generally known as the two-parameter normal ogive (2PNO) stating the probability ($\text {Pr}$) for a correctly solved item as $\text {Pr}(y_{ij}=1|\theta _i,\alpha _j,\beta _j) = \Phi (\alpha _j\theta _i-\beta _j)$, where $\theta _i$ denotes a scalar person parameter, $\alpha _j$ is a item discrimination parameter and $\beta _j$ denotes the item difficulty or item fixed effect. We adopt the standard normal cumulative distribution function $\Phi (\cdot )$ as the link function, as it offers computational advantages for MCMC based Bayesian estimation. Also, it allows for an alternative representation in terms of a threshold mechanism, which was first formalized in the context of individual level data by McKelvey and Zavoina (1975) and can be found for multivariate binary variables in Maddala (1983, p. 138). Extending towards the analysis of ordered polytomous item responses, see Samejima (1969), the observed item responses can be seen as a ordered polytomous version of an underlying continuous variable $y_{ij}^*=\alpha _j\theta _i-\beta _j+\varepsilon _{ij}$, where the independent and identically distributed error term $\varepsilon _{ij}$ follows a standard normal distribution. Then one can link the observed categorical and the underlying continuous variable using a threshold mechanism, namely

1 $$\begin{aligned} y_{ij}=\sum _{q=1}^{Q_j}(q-1){\mathcal {I}}\left( \kappa _{jq-1} < y_{ij}^* \le \kappa _{jq}\right) , \end{aligned}$$

where $\kappa _j=(\kappa _{j0},\kappa _{j1},\ldots ,\kappa _{jQ_j})'$ is the $(Q_j+1)$-dimensional vector of item category cutoff parameters and ${\mathcal {I}}(\cdot )$ denotes the indicator function. The resulting probability that respondent *i* achieves grade *q* on item *j*, given his latent trait and item parameters, is hence implied by

$$\begin{aligned} \text {Pr}\big (y_{ij}=q|\theta _i,\alpha _j,\beta _j,\kappa _j\big )=\Phi \big ( \kappa _{jq+1}-\big (\alpha _j\theta _i - \beta _j\big ) \big ) - \Phi \big (\kappa _{jq}-\big (\alpha _j\theta _i - \beta _j\big )\big ), \end{aligned}$$

thus nesting the binary case as well. This probability can be represented as in terms of the latent variables as $\int f(y_{ij},y_{ij}^*|\theta _i,\alpha _j,\beta _j,\kappa _j) {\textrm{d}}y_{ij}^*$, where

2 $$\begin{aligned} f\big (y_{ij},y_{ij}^*|\theta _i,\alpha _j,\beta _j,\kappa _j\big )=\frac{1}{\sqrt{2\pi }}\exp \left\{ -\frac{1}{2}\big (y_{ij}^*-\big (\alpha _j\theta _i-\beta _j\big )\big )^2\right\} {\mathcal {I}}\big (\kappa _{jy_{ij}}<y_{ij}^* <\kappa _{jy_{ij}+1}\big ).\nonumber \\ \end{aligned}$$

The necessary identifying restrictions for all parameters will be discussed jointly below.

IRT models are designed to directly link items and persons to a common scale. To enlarge their scope, the focus of analysis was broadened towards structural analysis by Muthén (1979) addressing the issue that persons may not only differ in terms of their competence, but also in terms of covariates which are correlated with their competence. The standard framework assuming $\theta _i$, $i=1,\ldots ,N$, to be identically and independently normally distributed can be extended to incorporate a conditional mean operationalized as $\text {E}[\theta _i|X_i]=X_i\gamma$, $i=1,\ldots ,N$. Thereby $X=(X_1',\ldots ,X_N')'=(X^{(1)},\ldots ,X^{(P)})$ in terms of row vectors $X_i$, $i=1,\ldots ,N$ and column vectors $X^{(p)}$, $p=1,\ldots ,P$ denotes a matrix of $N\times P$ individual specific covariates and $\gamma$ the corresponding vector of regression coefficients. When hierarchical clustering in observations is present, this needs to be incorporated in the model as well, as consideration of hierarchical data structures is an important prerequisite for valid inference on the relationship between explaining and latent variables. The multiple forms of population heterogeneity in educational research are reviewed in Muthén (1989) and Burstein (1980), whereas Greene (2004b) provides a discussion for economic applications of the panel probit model incorporating latent heterogeneity structures. Population heterogeneity may be considered in terms of a nested multilevel structure thereby assuming a composite population consisting of a finite number of *G* mutually exclusive groups indexed by $g=1,\ldots ,G$, where $L=(L_1,\ldots ,L_N)$ with $L_i\in \{1,\ldots ,G\}$, $i=1,\ldots ,N$ denotes the individual group membership. Within these groups, separate LRMs may hold. Sample stratification may be based on an explicitly observed cluster variable, e.g., gender or school type. This type of modeling dates back to the early works of Muthén and Christoffersson (1981) and Mislevy (1985), but without consideration of covariates except the cluster variable. Often, the specification is theory driven with the aim to discover substantial differences of covariate effects and variances for predefined groups. These differences are captured through the estimation of group-specific latent trait distributions. Additionally, hierarchical structures may be related to random effects. As in multilevel models there is a composite population consisting of clusters $c=1,\ldots ,C$, where the individual cluster membership is also known a-priori and is captured by $S=(S_1,\ldots ,S_N)$ with $S_i\in \{1,\ldots ,C\}$ for all $i=1,\ldots ,N$. While fixed group-specific regression parameters are suitable for a relative small number of groups, consideration of hierarchical structures with regard to schools or classes often implies a prohibitively large number of parameters. Difficulties regarding the computation and the statistical properties of the maximum likelihood estimator in this context were studied by Greene (2004a).^8^ Thus, the introduction of identically and independently normally distributed cluster-specific random effects $\omega =(\omega _1,\ldots ,\omega _C)$ offers an appropriate alternative or addition to the fixed effects approach. The most basic multilevel specification is the random intercept latent regression item response model. Depending on the specific hierarchical structure under consideration, combinations of both approaches are possible and allow for multiple hierarchical levels.

To illustrate, consider a model with nested hierarchical structure with $S_i=S_{i'}$ implying $L_i=L_{i'}$, i.e. individuals within the same cluster also refer to the same group, but not vice-versa, given as

3 $$\begin{aligned} \theta _{i}=\omega _{S_i}+X_{i}\gamma _{L_i}+\epsilon _{i}. \end{aligned}$$

Thereby $\epsilon _{i}$, $i=1,\ldots ,N$, is independently normally distributed with mean zero and heteroscedastic variance $\sigma ^2_{L_{i}}$. Likewise $\omega _{S_i}$ is independently normally distributed with mean zero and heteroscedastic variance $\upsilon ^2_{L_i}$. The assumed heteroscedasticity is hence a further way to implement features of (nested) hierarchical structures.^9^ We summarize all model parameters as $\psi =(\{\alpha _j,\beta _j,\kappa _j\}_{j=1}^J,\{\gamma _g,\sigma ^2_g,\upsilon ^2_g\}_{g=1}^G)$. The implied conditional covariance structure with regard to two elements of $\theta =(\theta _1,\ldots ,\theta _N)$ denoted with *i* and $i'$ can be described as

$$\begin{aligned} \text {Cov}(\theta _i,\theta _{i'}|\psi ,X,S,L)=\left\{ \begin{array}{ll} 0, &{}\quad \text {for } i\ne i'\text { and }S_i\ne S_{i'}, \\ \upsilon _{L_i}^2=\upsilon _{L_{i'}}^2, &{}\quad \text {for } i\ne i'\text { with } S_i=S_{i'}\text { and } L_i=L_{i'},\\ \sigma _{L_i}^2+\upsilon _{L_i}^2=\sigma _{L_{i'}}^2+\upsilon _{L_{i'}}^2, &{}\quad \text {for } i=i'\text { with } S_i=S_{i'}\text { and } L_i=L_{i'}. \end{array} \right. \end{aligned}$$

This covariance structure allows for group specific conditional variances but possibly similar or different correlations within clusters. The corresponding likelihood function in case of completely observed data is given as

4 $$\begin{aligned} f(Y|\psi ,X,S,L) = \int f(Y,Y^*,\theta ,\omega |\psi ,X,S,L) {\textrm{d}}Y^*{\textrm{d}}\theta {\textrm{d}}\omega . \end{aligned}$$

Thereby

5 $$\begin{aligned} f(Y,Y^*,\theta ,\omega |\psi ,X,S,L)= \left[ \prod _{i=1}^{N} f(y_i,y_{i}^*|\theta _i,\psi ) f(\theta _{i}|X_i,\psi ,\omega ,S_i,L_i)\right] f(\omega |\psi ,S,L), \end{aligned}$$

where $f(y_{i},y_{i}^*|\theta _i,\psi )=\prod _{j=1}^{J} f(y_{ij},y_{ij}^*|\psi ,\theta _i)$ with $f(y_{ij},y_{ij}^*|\psi ,\theta _i)$ as in Eq. (2),

$$\begin{aligned} f(\theta _{i}|X_i,\psi ,\omega ,S_i,L_i)=(2\pi )^{-\frac{1}{2}}\big (\sigma _{L_i}^{2}\big )^{-\frac{1}{2}}\exp \left\{ -\frac{1}{2\sigma _{L_i}^2}(\theta _{i}-(\omega _{S_i}-X_{i}\gamma _{L_i}))^2\right\} , \end{aligned}$$

and $f(\omega |\psi ,S,L)$ following a multivariate normal distribution with mean zero and covariance matrix $\text {diag}(\upsilon _{L_1}^2,\ldots ,\upsilon _{L_N}^2)$.

In case of completely observed data *Y* and *X*, the Bayesian model setup is then completed by an appropriate prior distribution $\pi (\psi )$. However, the estimation of IRT models is in general plagued by an identification problem, where the classical identification strategies impose restrictions on the parameter space. For the given model, the identification problem can be described as follows. First, the overall means of $y_{ij}^*$ are implied by the mean values of $\theta _i$, $\beta _j$, and $\kappa _j$, as well as the signs of $\alpha _j$. The mean values of $\theta _i$ in turn arise from the regression coefficients $\gamma _g$ in combination with the observed covariates $X_i$. Second, the scaling of $y_{ij}^*$ is implied by the scaling of $\theta _i$ and $\alpha _j$, where the scaling of $\theta _i$ arises from the variance parameters $\upsilon _g^2$ and $\sigma _g^2$. The given interdependencies lead to the fact that these parameters are not jointly identifiable. However, for given signs of $\alpha _j$ and mean values for two of the three quantities $\theta _i$, $\beta _j$, and $\kappa _j$, mean values for the remaining quantity become identifiable. The same holds for the scaling issue, where for given signs of $\alpha _j$ and a given scaling for one of the two quantities $\theta _i$ and $\alpha _j$, the remaining scaling becomes identifiable. The decision which mean and scaling parameters to fix is in principle arbitrary. However, for the considered hierarchical structures it is more convenient, also in terms of the implied sampling scheme, to restrict the item parameters $\alpha _j$, $\beta _j$, and $\kappa _j$. The typical choice as discussed in the literature by Fox (2010) and Albert and Chib (1997) imposes the following ordering and value constraints on the parameter space. With regard to the threshold parameter the restrictions can be formulated in terms of the condition $\prod _{j=1}^J {\mathcal {I}}(\kappa _{j0}=-\infty ,\kappa _{j1}=0< \kappa _{j2}<\cdots< \kappa _{jQ_j-1} < \kappa _{jQ_j}=+\infty )$, while for the item difficulties and discrimination parameters, we have ${\mathcal {I}}(\sum _{j=1}^J \beta _j=0)$ and ${\mathcal {I}}(\prod _{j=1}^J \alpha _j {\mathcal {I}}(\alpha _j>0)=1)$. Given these identifying restrictions, appropriate (conjugate) prior distributions can be formulated as given in Table 1. In the light of the Clifford–Hammersley theorem, see Robert and Casella (2004) for theorem and proof, the implied joint posterior distribution

6 $$\begin{aligned} f(\theta ,Y^*,\omega ,\psi |Y,X,L,S)\propto f(Y,Y^*,\theta ,\omega |\psi ,X,S,L)\pi (\psi ) \end{aligned}$$

is accessible in terms of the corresponding set of full conditional distributions. With $Z=\{Y,X,S,L\}$, we have

7 $$\begin{aligned} f(\theta ,Y^*,\omega ,\psi |Z)\propto \frac{f(\theta |{\tilde{Y}}^*,{\tilde{\omega }},{\tilde{\psi }},Z)}{f({\tilde{\theta }}|{\tilde{Y}}^*,{\tilde{\omega }},{\tilde{\psi }},Z)}\frac{f(Y^*|\theta ,{\tilde{\omega }},{\tilde{\psi }},Z)}{f({\tilde{Y}}^*|\theta ,{\tilde{\omega }},{\tilde{\psi }},Z)}\frac{f(\omega |\theta ,Y^*,{\tilde{\psi }},Z)}{f({\tilde{\omega }}|\theta ,Y^*,{\tilde{\psi }},Z)}\frac{f(\psi |\theta ,Y^*,\omega ,Z)}{f({\tilde{\psi }}|\theta ,Y^*,\omega ,Z)}, \end{aligned}$$

where the chosen sequence ordering $\theta ,Y^*,\omega ,\psi$ is arbitrary and  ${\tilde{\cdot }}$  denotes any admissible point of the indicated variable. The set of full conditional distributions resulting from Eq. (7) and employed within an MCMC algorithm taking the form of an iterative sequential Metropolis-Hastings (MH) within Gibbs sampling scheme to provide inference based on a sample from the posterior distributions is given in detail in Sect. 2.2.

**Table 1:** Prior specifications and MCMC starting values.

Parameter	Functional form	Probability distribution	Initialization
*Structural model*			
$\{\gamma _g\}_{g=1}^G$	$\propto \prod _{g=1}^G \exp \{-\frac{1}{2}(\gamma _g - \nu _{\gamma _g})'\Omega _{\gamma _g}^{-1}(\gamma _g - \nu _{\gamma _g}) \}$	Normal	$\{0\}_{g=1}^G$
$\{\sigma _g^2\}_{g=1}^G$	$\propto \prod _{g=1}^G{(\sigma _g^2)}^{-a_{\sigma _g^2}-1}\exp \left\{ -\frac{b_{\sigma _g^2}}{(\sigma _g^2)}\right\} \mathcal {I}\big (\sigma _g^2>0\big )$	Inverse gamma	$\{1\}_{g=1}^G$
$\{\upsilon _g^2\}_{g=1}^G$	$\propto \prod _{g=1}^G{(\upsilon _g^2)}^{-a_{\upsilon _g^2}-1}\exp \left\{ -\frac{b_{\upsilon _g^2}}{(\upsilon _g^2)}\right\} \mathcal {I}\big (\upsilon _g^2>0\big )$	Inverse gamma	$\{1\}_{g=1}^G$
*Item characteristics*			
$\{\alpha _j\}_{j=1}^J$	$$\begin{array}{l} \propto \prod _{j=1}^J\exp \left\{ -\frac{1}{2\Omega _{\alpha _j}^2} \big (\alpha _j-\nu _{\alpha _j}\big )^2\right\} \mathcal {I}\big (\prod _{j=1}^J \alpha _j\mathcal {I}(\alpha _j > 0)=1\big )\end{array}$$	Defective truncated normal	$\{1\}_{j=1}^J$
$\{\beta _j\}_{j=1}^J$	$\propto \prod _{j=1}^J\exp \left\{ -\frac{1}{2\Omega _{\beta _j}^2} (\beta _j-\nu _{\beta _j})^2\right\} \mathcal {I}\big (\sum _{j=1}^J \beta _j=0\big )$	Defective normal	$\{0\}_{j=1}^J$
$\{\kappa _j\}_{j=1}^J$	$$\begin{array}{l}\propto \prod _{j=1}^J \frac{\exp \left\{ -\left( \frac{(\ln \kappa _{j2}-\nu _{\kappa _{j2}})^2}{2\Omega ^2_{\kappa _{j2}}}+\sum _{q=3}^{Q_j}\frac{(\ln (\kappa _{jq} -\kappa _{jq-1})-\nu _{\kappa _{jq}})^2}{2\Omega _{\kappa _{jq}^2}}\right) \right\} }{\kappa _{j2}\prod _{q=3}^{Q_j}(\kappa _{jq}-\kappa _{jq-1})}\mathcal {I}(\kappa _{j1}=0<\kappa _{j2}<\cdots <\kappa _{jQ_j})\end{array}$$	Defective lognormal	$\{0,1,2,\ldots ,Q_j-1\}_{j=1}^J$
*Missing values*			
$X_{\textrm{mis}}$	$\propto$ observed sample distribution	Nonparametric	Random draws

The hyperparameters are chosen as $\{\nu _{\gamma _g} = 0, \Omega _{\gamma _g} = 100\textbf{I}_{P+1},a_{\sigma _g^2}=3,\ b_{\sigma _g^2} = 1, a_{\upsilon _g^2}=3,\ b_{\upsilon _g^2} = 1\}_{g=1}^G$ and $\{\nu _{\alpha _j}=0,\Omega _{\alpha _j}=100, \nu _{\beta _j}=0,\Omega _{\beta _j}=100,\{\nu _{\kappa _{jq}}=0,\Omega ^2_{\kappa _{jq}}=100\}_{q=1}^{Q_j}\}_{j=1}^J$. The hyperparameters for the inverse gamma distribution are chosen to provide finite variance and smallest possible prior sample size.

Next, we will discuss the handling of missing values. Given the factorization of the likelihood described in Eqs. (2) and (5), handling of missing values in item responses $Y=(Y_{\text {obs}},Y_{\text {mis}})$ is directly possible by dropping the corresponding elements $Y_{\text {mis}}$ from the likelihood. That means, per item *j*, only the observed $y_{ij}$ are used to estimate the parameters. An alternative approach of handling missing values in *Y* may be to consider missing values as wrong answers. Our approach is also fully compatible with von Hippel (2007) suggesting to consider draws of $Y_{\text {mis}}$ from the posterior predictive distribution for the specification of the full conditional distributions of the missing values of the covariate variables *X* but not using them for analysis.^10^

However, when facing partially observed *X* one has to think of an appropriate missing data technique to facilitate estimation. In the following, we will denote $X=(X_{\text {obs}}, X_{\text {mis}})$. In the context of the considered model structure, the latent variables and hierarchical structures take the role of sufficient statistics and may play a crucial role for implementing appropriate models defining the uncertainty associated with missing values $X_{\text {mis}}$. We suggest to handle missing values $X_{\text {mis}}$ by means of DA, as this allows for advantageous use of the latent and hierarchical model structures within the modeling of missing values by means of Rao-Blackwellization and due to a lower dimensional representation of the relevant information also reducing the computational burden.^11^ The advantages relate to gains in statistical efficiency in estimmation of $\psi$ captured by the bias, root mean square error, and coverage. Hence, the augmented posterior distribution

$$\begin{aligned} f(\theta ,Y^*,\omega ,\psi ,X_{\text {mis}}|Y,X_{\text {obs}},S,L)\propto f(Y,Y^*,\theta ,\omega |\psi ,X,S,L)\pi (X_{\text {mis}}|X_{\text {obs}},\psi )\pi (\psi ), \end{aligned}$$

incorporating an appropriate prior distribution $\pi (X_{\text {mis}}|X_{\text {obs}},\psi )$, is of interest and subject to inference. The characterization in terms of the full conditional distributions given in Eq. (7) is then extended as follows. With ${\tilde{Z}}=\{Y,X_{\text {obs}},{\tilde{X}}_{\text {mis}},S,L\}$, we have

8 $$\begin{aligned} f(\theta ,Y^*,\omega ,\psi ,X_{\text {mis}}|Y,X_{\text {obs}},S,L)\propto f(\theta ,Y^*,\omega ,\psi |{\tilde{Z}})\frac{f(X_{\text {mis}}|\theta ,Y^*,\omega ,\psi ,Y,X_{\text {obs}},S,L)}{f({\tilde{X}}_{\text {mis}}|\theta ,Y^*,\omega ,\psi ,Y,X_{\text {obs}},S,L)}, \end{aligned}$$

thereby augmenting the MCMC sampling scheme.^12^

The suggested sequential sampling is also well suited to deal with regression specifications involving cross products of variables considered in *X*. Given an initialization of $X_{\text {mis}}$ and thus the involved cross products, missing values for one variable can be drawn. If this variable is involved in cross products, these cross products are updated. This procedure is then repeated for each variable in *X*. In order to establish highly flexible modeling of the distributions of $X_{\text {mis}}$ and allow for handling of a possibly large number of background variables, we adopt sequential recursive classification and regression trees in combination with sampling via a Bayesian bootstrap (CART-BB) for the construction of full conditional distributions, see Burgette and Reiter (2010) and Rubin (1981). Modeling the full conditional distributions of missing values in this way is compatible with assuming prior distributions for the missing values proportional to the empirical densities observed for each variable, see also Table 1.^13^ This choice is motivated by the flexibility of CART-BB to handle variables of any scale and the potential to cope with nonlinear relationships among the variables, see also Doove et al. (2014). The application of CART-BB to model the full conditional distributions of missing values is particularly useful because the analyst does not need to specify the full conditional distributions of missing values (imputation models) explicitly. The complete set of full conditional distributions and further details referring to the augmented parameter vector are provided in the following. We label the suggested Bayesian estimation approach using data augmentation and sequential recursive partitioning classification and regression trees combined with a Bayesian bootstrap for handling missing values in covariate variables as DART approach.

### Bayesian Inference

Bayesian inference is based on a posterior sample generated via the following MCMC algorithm iteratively sampling from the set of full conditional distributions.^14^ The algorithm is based on the blocking scheme $y_{11}^*,\ldots ,y_{NJ}^*$, $\alpha _1,\beta _1,\ldots ,\alpha _J,\beta _J$, $\kappa _1,\ldots ,\kappa _J$, $X_{\text {mis}}^{(1)},\ldots ,X_{\text {mis}}^{(P)}$, $\theta _1,\ldots$, $\theta _N$, $\gamma _1,\ldots ,\gamma _G$, $\sigma _1^2,\ldots ,\sigma _G^2$, $\omega _1,\ldots ,\omega _C^2$, $\upsilon _1^2,\ldots ,\upsilon _G^2$, where the initialization of all quantities except $y_{11}^*,\ldots ,y_{NJ}^*$ is described in Table 1 and initial values for $\theta$ and $\omega$ are drawn from standard normal distributions. An implementation of this MCMC sampling algorithm in R is available within the supplementary material. The set of full conditional distributions can be described as follows.

$$f(y_{ij}^*|\cdot )$$
The full conditional distributions of the random variables $y_{ij}^*$, $i=1,\ldots ,N$ and $j=1\ldots ,J$ are independent and sampled from a truncated normal distribution with moments $\mu _{y_{ij}^*} = \alpha _j\theta _{i}-\beta _j$ and $\sigma ^2_{y_{ij}^*} = 1$, where the truncation sphere is $(\kappa _{jy_{ij}},\kappa _{jy_{ij}+1})$.
$$f(\alpha _1,\beta _1,\ldots ,\alpha _J,\beta _J|\cdot )$$
Note that for the assumed model structure in absence of the identifying restrictions all full conditional distributions of the item parameters $\xi _j=(\alpha _j,\beta _j)'$, $j=1,\ldots ,J$ are mutually independent. In the presence of the identifying restrictions, however an arbitrarily chosen single element, say $\xi _{j'}$, is completely determined by the others $J-1$ item parameters, i.e. $\xi _{j'}=((\prod _{j\ne j'}\alpha _j)^{-1},-\sum _{j\ne j'}\beta _j)$. In this sense, the joint distribution of all item parameters is defective, as the distribution of the element implied by the other elements is not specified. Further, sampling from the full conditional distribution of item parameters $\xi _j$ in absence of identifying restrictions can be characterized in terms of the linear regression equation $y_{j}^*=H\xi _j+\epsilon _j$, where *H* is a $N\times 2$ auxiliary matrix consisting of $\theta$ and $-\iota _N$, where $\iota _N$ denotes a $N\times 1$ vector of ones. Since $\epsilon _j$ is normally distributed, $\xi _j$ is proportional to a bivariate truncated normal distribution with covariance matrix and mean vector
  $$\begin{aligned} \Sigma _{\xi _j} = \big (H'H+\Omega _{\xi _j}^{-1}\big )^{-1} \quad \text {and}\quad \mu _{\xi _j} = \Sigma _{\xi _j}\big (H'y_{j}^* + \Omega _{\xi _j}^{-1}\nu _{\xi _j}\big ). \end{aligned}$$
  The positivity constraints on the item discrimination parameters causing the truncation are handled via accept reject sampling. In each iteration sampling is performed until a draw is accepted. The values of the hyperparameters $\nu _{\xi _j}$ and $\Omega _{\xi _j}$ are chosen as given in Table 1. Note that for any possible subset containing $J-1$ item parameters, the remainder item parameters, say $\xi _{j'}$, are implied by the assumed identifying restrictions. Although this element is determined by all other elements, the data driven information contained within the above regression is not incorporated in the characterization of these item parameters. Further, *J* equivalent possibilities exist to characterize the redundant element. Hence, incorporating these *J* alternative possibilities to draw from the full conditional distribution into a single raw via averaging seems preferable in order to use all available data based information and thus improve mixing and convergence issues. Given draws for $\alpha =(\alpha _1,\ldots ,\alpha _J)$ and $\beta =(\beta _1,\ldots ,\beta _J)$ averaging the *J* characterizations is possible in terms of the geometric mean and the arithmetic mean resulting in $\alpha =(\alpha _1(\prod _{j=1}^J\alpha _j)^{-\frac{1}{J}},\ldots ,\alpha _J(\prod _{j=1}^J\alpha _j)^{-\frac{1}{J}})$ and $\beta =(\beta _1-\frac{1}{J}\sum _{j=1}^J\beta _j,\ldots ,\beta _J-\frac{1}{J}\sum _{j=1}^J \beta _j)$. We refer to this approach to handling identifying restrictions as a kind of marginal data augmentation, see among others Imai and van Dyk (2005).
$$f(\kappa _j|\cdot )$$
Draws from the mutually independent full conditional distributions of the item category cutoff parameters $\kappa _j$ are retained via a MH step following Albert and Chib (1997). To perform this sampling step it is convenient to consider a reparameterization of the elements $\kappa _{j2},\ldots ,\kappa _{jQ_j-1}$, where $\kappa _{jq}=\sum _{w=2}^q \exp \{\tau _{jw}\}$ for all $j=1,\ldots ,J$ and $q=2,\ldots ,Q_j-1$. The threshold parameters can then be stated as $\kappa _j=(-\infty ,0,\kappa _{j2},\ldots ,\kappa _{jQ_j-1},\infty )=h(\tau _j)=(h_{j0},h_{j1},h_{j2},\ldots ,h_{jQ_j-1},h_{jQ_j})=(-\infty ,0,\exp \{\tau _{j2}\},\exp \{\tau _{j2}\}+\exp \{\tau _{j3}\},\ldots ,\sum _{q=2}^{Q_j-1}\exp \{\tau _{jq}\},\infty )$. Given the prior for $\kappa _j$ this transformation induces a multivariate normal prior for $\tau _j=(\tau _{j2},\ldots ,\tau _{jQ_j-1})$ given as
  $$\begin{aligned} \pi (\tau _j)\propto \prod _{q=2}^{Q_j-1} \exp \left\{ -\frac{1}{2\Omega _{\kappa _{jq}}^2}\big (\tau _{jq}-\nu _{\kappa _{jq}}\big )^2\right\} . \end{aligned}$$
  Hence, the posterior and thus full conditional distribution can be reformulated in terms of $\tau _j$. To generate a draw from the full conditional of $\tau _j$, we choose as a proposal a multivariate *t*-distribution with mean vector $m_j$, covariance matrix $V_j$ and $Q_j-2$ degrees of freedom, where
  $$\begin{aligned} m_j=\arg \underset{\tau _j}{\max }\ \text{ ln }\{f(y_{j}|\xi _j,h(\tau _j),\psi ,\theta )\pi (\tau _j)\} \end{aligned}$$
  and $V_j$ is the inverse of the Hessian of $\text{ ln }\{f(y_{j}|\xi _j,h(\tau _j),\psi ,\theta )\pi (\tau _j)\}$ evaluated at $m_j$. Note that $f(y_j|\xi _j,h(\tau _j),\theta ,\psi )=\prod _{i=1}^N [\Phi (h_{jy_{ij}+1}-(\alpha _j\theta _i-\beta _j))-\Phi (h_{jy_{ij}}-(\alpha _j\theta _i-\beta _j))]$. The probability of accepting candidate values $\tau _j^{\text{ cand }}$ is given as
  $$\begin{aligned} a_{\tau _j}=\min \left\{ 1,\frac{f\big (y_{j}|\xi _j,h\big (\tau ^{\text{ cand }}_j\big ),\psi ,\theta \big )\pi \big (\tau ^{\text{ cand }}_j\big )}{f\big (y_{j}|\xi _j,h(\tau _j),\psi ,\theta \big )\pi (\tau _j)}\frac{f_t\big (\tau _j|m_j,V_j,Q_j-2\big )}{f_t\big (\tau ^{\text{ cand }}_j|m_j,V_j,Q_j-2\big )}\right\} . \end{aligned}$$
  The acceptance rates within the simulation study and the empirical application where found to be at least 0.95. A draw for $\kappa _j$ is then implied by $h(\tau _j)$. The chosen hyperparameter values for $\Omega _{\kappa _{jq}}^2$ and $\nu _{\kappa _{jq}}$ are given in Table 1.
$$f(X_{\text {mis}}^{(p)}|\cdot )$$
Values of $X_{\text {mis}}$ are sampled sequentially for each column vector $X^{(p)}$, $p=1,\ldots ,P$ in two steps. Let $X_{\text{ com }}^{({\setminus } p)}=(X_{\text{ obs }}^{({\setminus } p)},X_{\text{ mis }}^{({\setminus } p)})$ denote the completed matrix of conditional variables in $X_{\text {}}$ except column *p*, with the operator $\setminus p$ meaning without *p*.^15^ First, a decision tree is built for $X_{\text{ com }}^{({\setminus } p)}$ conditional on the corresponding values of all remaining variables $X_{\text {com}}^{(\setminus p)}$ as well as conditional on $\theta ,\omega , S,L$, and *Y*. A further possibility is to consider only subsets of the conditioning variables $\theta$, $\omega$, *S*, *L*, and *Y*. To incorporate a priori uncertainty on the hyperparameters of the sequential partitioning regression trees, we build trees with a randomly varying minimum number of elements within nodes. Every missing observation can then be assigned to a node and thus a grouping of observations implied by the binary partition in terms of the conditioning variables. The values within each node provide access to an empirical distribution function serving as an approximation to the full conditional distribution of a missing value and thus as the key element for running the data generating mechanism for missing values. With prior distributions of missing values proportional to observed data densities, draws from the empirical distribution function within a node correspond to draws from the full conditional distributions of missing values. To account for the estimation uncertainty of the full conditional distribution, the Bayesian bootstrap is applied to the assigned group of observations, see Rubin (1981). Thereby, the uncertainty regarding the estimated empirical distribution implied by the proposed set of observed values is fully considered.^16^ The considered approach further offers the flexibility to consider any function of observed or augmented data within the set of conditioning variables as well. Next to the matrices $Y^*$ and *Y* also statistics thereof might be considered. This may include draws of missing values in *Y* or $Y^*$ from the posterior predictive distributions as suggested by von Hippel (2007). In case of restricting the analysis to observed values of *Y* only as in the empirical illustration, additionally missing categories might be considered. Note that this is the default of the R function rpart within the implementation of the CART-BB algorithm, see Therneau and Atkinson (2018). Further, also group specific or individual specific specifications of the full conditional distributions could be adapted by consideration of group specific variables within the set of conditioning variables only, i.e. create a binary partition only for those values fulfilling the conditions $L_i=g$ or $S_i=c$. The sampled $X_{\text {mis}}$ values allow to refer to an updated completed matrix of covariates in all other steps of the MCMC algorithm.
$$f(\theta _i|\cdot )$$
The full conditional distributions for $\theta _i$, $i=1,\ldots ,N$ are elementwise conditionally independent. Let $B_{i}=y_{i}^* + \beta$. This allows for stating the conditional distribution of the individual abilities as normal with moments
  9 $$\begin{aligned} \sigma ^2_{\theta _{i}} = \big (\alpha '\alpha + \sigma _{S_i}^{-2}\big )^{-1} \quad \text {and}\quad \mu _{\theta _{i}} = \sigma ^2_{\theta _{i}}\left( \alpha ' B_{i} + \sigma _{S_i}^{-2}(\omega _{S_i} + X_{i}\gamma _{L_i})\right) . \end{aligned}$$
$$f(\gamma _g|\cdot )$$
To sample from the full conditional distributions of the regression coefficients, let $D^C$ denote a $N\times C$ design matrix of zeros and ones. Each row of $D^C$ has a single entry 1 indicating the respondents’ cluster membership $S_i$. The operator [*g*] selects the elements of $\theta$, respectively the rows of *X* and $D^C$ for which the condition $L_i=g$ holds. Further, let $\Sigma _{\epsilon }$ be a $N_g\times N_g$ diagonal matrix with elements $\sigma ^2_{\epsilon ,g}$. Draws from the conditional distribution of $\gamma _g$ are obtained from a multivariate normal with covariance matrix and mean vector
  $$\begin{aligned} \Sigma _{\gamma _g} = \big (X_{[g]}'\Sigma _\epsilon ^{-1}X_{[g]} + \Omega _{\gamma _g}^{-1}\big )^{-1} \text { and } \mu _{\gamma _g} = \Sigma _{\gamma _g}\big (X_{[g]}'\Sigma _\epsilon ^{-1}(\theta _{[g]} - D^C_{[g]}\omega ) + \Omega _{\gamma _g}^{-1}\nu _{\gamma _g}\big ). \end{aligned}$$
  Note that values of hyperparameters $\nu _{\gamma _g}$ and $\Omega _{\gamma _g}$ are chosen as given in Table 1.
$$f(\sigma _{g}^2|\cdot )$$
In each group *g* you find $C_g$ clusters and $N_g$ respondents. It holds that $\sum _{g=1}^{G}C_g=C$ and $\sum _{g=1}^{G}N_g=N$. Choosing a conjugate prior, the full conditional distribution of $\sigma _{g}^2$ is distributed inverse gamma with shape and scale parameters
  $$\begin{aligned} a_{\sigma _{g}^2} = a^0_{\sigma _{g}^2} + N_g/2, ~ b_{\sigma _{g}^2}&= \Bigg (b^0_{\sigma _{g}^2} + \frac{1}{2}\big (\theta _{[g]} - D^C_{[g]}\omega - X_{[g]}\gamma _g\big )'\big (\theta _{[g]} - D^C_{[g]}\omega - X_{[g]}\gamma _g\big )\Bigg )^{-1}, \end{aligned}$$
  where the values of the hyperparameters $a^0_{\sigma _g^2}$ and $b^0_{\sigma _{g}^2}$ are chosen as given in Table 1.
$$f(\omega _c|\cdot )$$
Let the operator [*c*] select the elements of $\theta$, respective the rows of *X* belonging to cluster *c* and $N_c$ be the total number of persons in cluster *c*. The cluster-specific random intercepts $\omega _c$ are conditionally independent and follow a full conditional distribution given as a normal distribution with moments
  $$\begin{aligned} \sigma ^2_{\omega _c}&= \left( \upsilon ^{-2}_{S_c} + N_c/\sigma ^2_{S_c}\right) ^{-1} \quad \text {and}\quad \mu _{\omega _c} = \sigma ^2_{\omega _c}\left( \sigma ^{-2}_{S_c}(\theta _{[c]} - X_{[c]}\gamma _{S_c})'\iota _{N_c}\right) . \end{aligned}$$
  The chosen values for hyperparameters are given in Table 1.
$$f(\upsilon _g^2|\cdot )$$
Given a conjugate prior and making use of the operator [*g*], $\upsilon _{\omega ,g}^2$ is distributed inverse gamma with shape and scale parameter
  $$\begin{aligned} a_{\upsilon _g^2} = a^0_{\upsilon _g^2} + C_g/2 \quad \text {and}\quad b_{\upsilon _g^2} = \left( b^0_{\upsilon _{g}^2} + 0.5\omega _{[g]}'\omega _{[g]}\right) ^{-1}. \end{aligned}$$
  Note that values of hyperparameters $a^0_{\upsilon _g^2}$ and $b^0_{\upsilon _{g}^2}$ are chosen as given in Table 1.

Given this MCMC algorithm, parameter estimates and functions of interest thereof can be readily obtained from the MCMC output denoted as $\{\psi ^{(r)},\theta ^{(r)},\omega ^{(r)}\}_{r=1}^R$ with *R* denoting the number of iterations after burn-in. Deciding for an absolute loss function, the estimates are implied by the medians of the posterior sample. Their calculation does not involve the application of any combining rules as for other approaches to handle missing values. If relevant, also the MCMC output with regard to the augmented quantities $\{Y^{*,(r)}, X^{(r)}=(X_{\text {obs}},X_{\text {mis}}^{(r)})\}_{r=1}^R$ may be considered as well. To illustrate, given the hierarchical model structure, within group correlation may as well be of interest, i.e.

$$\begin{aligned} \text {Cor}(\theta _i,\theta _{i'}|\psi ,X,S_i=S_{i'}=g,i\ne i')=\frac{\nu _g^2}{\nu _g^2+\sigma _g^2} \end{aligned}$$

with the corresponding estimator given as

$$\begin{aligned} \widetilde{\text {Cor}}(\theta _i,\theta _{i'}|\psi ,X,S_i=S_{i'}=g,i\ne i')= \text {median} \left\{ \frac{\nu _g^{2(r)}}{\nu _g^{2(r)}+\sigma _g^{2(r)}}\right\} _{r=1}^R. \end{aligned}$$

Next, the effects of changes in *X* on the individual competence level conditional on school type *g* (CE) might be of interest. Additionally, also the relative effects to another school type $g'$ (RE) or the conditional effects in standardized form (CSE), see e.g. Nieminen et al. (2013), can be considered, i.e.

10 $$\begin{aligned} {\text {CE}}_{X,g}=\gamma _g,\quad {\text {RE}}_{X,g,g'}=\gamma _g-\gamma _{g'},\;{\text {and}} \; {\text {CSE}}_{X,g}=\frac{\text {sd}[X_{[g]}]}{\text {sd}[\theta _{[g]}]}\gamma _g, \end{aligned}$$

where $\text {sd}$ denotes the vector of standard deviations of the column vectors in $X_{[g]}$. Also context effects in the form of ceteris paribus effects can be considered, e.g. ${\text {CP}}=\text {E}[\theta _i|X_i,\psi ,L_i=g]-\text {E}[\theta _i|X_i,\psi ,L_i=g']=X_i(\gamma _g-\gamma _{g'})$ or ${\text {CPA}}=\text {E}[\theta _i|X_i,\psi ,L_i=g,S_i=c,y_i^*,y_i]-\text {E}[\theta _i|X_i,\psi ,L_i=g',y_i^*,y_i]=\frac{1}{C}\sum _{c=1}^C \mu _{\theta _i}(X_i,L_i=g,S_i=c,\psi ,\omega _c,y_i^*)-\mu _{\theta _i}(X_i,L_i=g',S_i=c,\psi ,\omega _c,y_i^*)$, where $\mu _{\theta _i}(\cdot )$ is given in Eq. (9).

Estimates of conditional, relative and conditional standardized effects are readily available as

$$\begin{aligned} {\widetilde{{\text {CE}}}}_{X,g}= & {} \text {median}\left\{ \gamma _g^{(r)}\right\} _{r=1}^R,\quad {\widetilde{{\text {RE}}}}_{X,g}=\text {median}\left\{ \gamma _g^{(r)}-\gamma _{g'}^{(r)}\right\} _{r=1}^R,\\ \text {and}\quad {\widetilde{{\text {CSE}}}}_{X,g}= & {} \text {median} \left\{ \frac{\text {sd}[X_{[g]}^{(r)}]}{\text {sd}[\theta _{[g]}^{(r)}]}\gamma _g^{(r)}\right\} _{r=1}^R, \end{aligned}$$

whereas for the context effects we have ${\widetilde{{\text {CP}}}}=\text {median}\left\{ X_i^{(r)}(\gamma _g^{(r)}-\gamma _{g'}^{(r)})\right\} _{r=1}^R$ and

$$\begin{aligned} {\widetilde{{\text {CPA}}}}= & {} \text {median}\left\{ \frac{1}{C}\sum _{c=1}^C\left( \mu _{\theta _i}\big (X_i^{(r)},L_i=g,S_i=c\psi ^{(r)},\omega _c^{(r)},y_i^{*,(r)}\big )\right. \right. \\{} & {} \quad \left. \left. - \mu _{\theta _i}\big (X_i^{(r)},L_i=g',S_i=c,\psi ^{(r)},\omega _c^{(r)},y_i^{*,(r)}\big )\right) \right\} _{r=1}^R. \end{aligned}$$

Note that measures of uncertainty, e.g. posterior standard deviation or highest posterior density intervals, are likewise directly accessible without use of combining rules.

Finally, note that computation of the marginal data likelihood, i.e.

$$\begin{aligned} f(Y|X_{\textrm{obs}},S,L)=\int f(Y|\psi ,X,S,L)f(X_{\text {mis}}|X_{\text {obs}},\psi )f(\psi )dX_{\text {mis}}d\psi , \end{aligned}$$

involved in Bayes factors to allow for non-nested model comparison is possible along the lines suggested by Chib (1995), Chib and Jeliazkov (2001) and Aßmann and Preising (2020) in the context of linear dynamic panel models.

## Simulation Study

We assess the proposed strategy via a simulation study. To illustrate the possible gains arising from the handling of missing values by means of DA, we consider as benchmarks estimation without missing values, i.e., before any values have been discarded from the data sets (BD), estimation of complete cases only (CC), and a third benchmark situation mimicking the situation of handling missing values without latent structures, i.e., handling of missing values in an imputation sense before estimating the model of interest (IBM). For the IBM benchmark, the full conditional distributions of missing values are also constructed via CART-BB by using information from observable variables only. For this, we consider

$$\begin{aligned} f_{\text {IBM}}\left( X_{\text {mis}}^{(p)}|X_{\text {com}}^{(\setminus p)},Y,S, L\right) ,\quad p=1,\ldots ,P. \end{aligned}$$

The IBM strategy conditions on all observables $(Y,X_{\text {obs}},S,L)$ but not on latent model structures like $\theta$ or $\omega$.^17^ These three benchmarks are contrasted with the suggested Bayesian estimation approach DART. Within the DART approach, we will add to the considered observable set of conditioning variables also the latent variables $\theta$ and $\omega$ to assess the full conditional distribution of $X_{\text {mis}}$, i.e.

$$\begin{aligned} f_{\text {DART}}\left( X_{\text {mis}}^{(p)}|X_{\text {com}}^{(\backslash p)},Y,\theta ,\omega ,S, L\right) ,\quad p=1,\ldots ,P. \end{aligned}$$

Next, we will consider also a modified version of the DART approach, labeled DART-m. We discard *Y* and *S* from the set of conditioning variables entering the CART-BB algorithm. This illustrates that the latent variables $\theta$ and $\omega$ serve as a kind of sufficient statistics of *Y* and *S*. When specifying the full conditional distributions of missing values $X_{\text {mis}}$ the sufficient statistics allow for incorporation of the relevant information but provide a more parsimonious representation of this information leading to a noticeable reduction in computation time.

The simulation study is based on the following data generating process (DGP), where the comparison is based on averaged estimation over ${\mathcal {S}}=1000$ replications referring to the same DGP. The considered DGP satisfies the following conditions. The response matrix *Y* is simulated assuming the model outlined in Eqs. (1), (2) and (3) with a sample setup of $N=4000$ students allocated equally to $C=20$ schools which belong to either one of $G=2$ school types. Thus, there are 200 students per school and 10 schools per school type corresponding to a nested hierarchical structure. The respondents face a test of altogether $J=20$ items of which the first 18 are binary and the last two are ordinal with $Q_{19}=Q_{20}=4$ categories. The *J* discrimination and difficulty parameters are fixed across replications and were obtained once via drawing from uniform distributions in the interval (0.7, 1.3) for discrimination and $(-\,0.7,0.7)$ for difficulty parameters respectively. To fulfill the identifying restrictions, the item difficulty and discrimination parameters are transformed in terms of the geometric and arithmetic mean respectively, see also Sect. 2.2 for details. Finally, the item category cutoff parameters for the two ordinal items are set to $\kappa _{19}=(0,0.5,1)'$ and $\kappa _{20} = (0,0.7,1.4)'$.

We consider three covariates with two covariates $X^{(p)}$, $p=2,3$, capturing individual differences in the latent trait $\theta _i$. Adding a constant, the regressor matrix can be stated as $X=(\iota _N, X^{(2)}, X^{(3)})$. Since participants in large-scale studies are often heterogeneous, we also map this circumstance in our simulation study. The chosen DGP leans towards the data situation in empirical surveys such as the NEPS, as we consider heterogeneity between groups of individuals. Therefore $X^{(2)}$ is sampled from a Bernoulli distribution with $\Pr (X_{i,g=1}^{(2)} = 1) =0.3$ for group 1 ($g=1$) and $\Pr (X_{i,g=2}^{(2)} = 1) =0.6$ for group 2 ($g=2$). $X^{(3)}$ is sampled from a normal distribution with school specific means and a variance set to one. The overall means in group 1 are chosen to be smaller compared to group 2. The corresponding parameters of the population model are set to $\gamma _1 = (-\,0.5,0.4,0.2)'$, $\gamma _2 = (1,0.2,-\,0.2)'$, $\sigma ^2_{1} = 0.64$, $\sigma ^2_{2} = 0.36$, $\upsilon ^2_{1} = 0.81$ and $\upsilon ^2_{2} = 0.49$. The simulation study consists out of four missing scenarios. For scenarios 1 and 2 the missing rates for $X^{(2)}$ and $X^{(3)}$ depend exclusively on the latent trait variable $\theta$. As suggested by a reviewer, dependence of the missing probability on the latent variable $\theta$ suggests to characterize the mechanism to be approximately at random, since the latent variable $\theta$ becomes estimable in the considered model framework via observable quantities. For scenario 3 missing probabilities are determined by weighted sum scores depending on the observed variables $X^{(2)}$, $X^{(3)}$, and the latent variable $\theta$. The scenario 4 is similar to scenario 3, but missing in $X^{(3)}$ depends itself on $X^{(3)}$ thus characterizing the mechanism to be not at random. For further details on the four described missing scenarios, see Table 2.

**Table 2:** Simulated missing data mechanisms.

Missing mechanisms	Average missing rates			Results
	$X^{(2)}$ (%)	$X^{(3)}$ (%)	Total (%)	
$$\begin{array}{l} \textrm{Scenario 1}\\ \Pr (X_i^{(2)} = \text {NA}) = \Phi (-1.2-\theta _i) \\ \Pr (X_i^{(3)} = \text {NA}) = \Phi (-0.8-\theta _i) \end{array}$$	20	25	33	Table 3
$$\begin{array}{l} \textrm{Scenario 2} \\ \Pr (X_i^{(2)} = \text {NA}) = \Phi (-0.15-\theta _i) \\ \Pr (X_i^{(3)} = \text {NA}) = \Phi (0.3-\theta _i) \end{array}$$	40	50	60	Table 4
$$\begin{array}{l} \textrm{Scenario 3} \\ X_i^{(2)}= \text {NA, if}\quad 1/\left( 1+e^{-w_{1i}}\right)> 0.75 \\ \text {with } w_{1i} = ( 0.2 X^{(3)}_i + 0.4 \theta _i + \tau _{1i}), \quad \tau _{1}\sim N(0,1) \\ X_i^{(3)}= \text {NA, if}\quad 1/\left( 1+e^{-w_{2i}}\right) > 0.65 \\ \text {with } w_{2i} = ( 0.1 X^{(2)}_i + 0.3 \theta _i + \tau _{2i}), \quad \tau _{2}\sim N(0,1) \\ \end{array}$$	20	36	46	Table 5
$$\begin{array}{l} \textrm{Scenario 4} \\ X_i^{(2)}= \text {NA, if}\quad 1/\left( 1+e^{-w_{1i}}\right)> 0.75 \\ \text {with } w_{1i} = ( 0.2 \tau _{1i} X^{(3)}_i + 0.4 \tau _{2i} \theta _i + \tau _{3i}), \quad \tau _{1,2,3}\sim N(0,1) \\ X_i^{(3)}= \text {NA, if}\quad 1/\left( 1+e^{-w_{2i}}\right) > 0.65 \\ \text {with } w_{2i} = ( 0.1 \tau _{4i} X^{(3)}_i + 0.3 \tau _{5i} \theta _i + \tau _{6i}), \quad \tau _{4,5,6}\sim N(0,1) \end{array}$$	17	28	40	Table 6

As the missing mechanisms depend on latent variables, the scenarios 1–3 can be characterized as approximately missing at random and scenario 4, where the missing probability depends on the variable itself as missing not at random. All simulation runs have been performed with 25,000 Gibbs iterations with the first 5000 iterations as burn-in.

**Table 3:** Simulation study (scenario 1, missing rates: $X_1=19\%$, $X_2=26\%$, overall = 33%)—True parameter values, mean posterior medians and standard deviations, RMSEs and coverage ratios of structural parameter (regression coefficients, variance parameters) over 1000 replications obtained from BD, CC, IBM and DART.

	True	Average					Averaged standard deviation				
		BD	CC	IBM	DART	DART-m	BD	CC	IBM	DART	DART-m
Runtimes (min)		92	65	340	366	229					
Regression coefficient											
$\gamma _{0,1}$	$-$ 0.500	$-$ 0.503	0.009	$-$ 0.516	$-$ 0.508	$-$ 0.511	0.260	0.206	0.261	0.263	0.262
$\gamma _{1,1}$	0.400	0.398	0.288	0.330	0.356	0.360	0.044	0.052	0.048	0.057	0.058
$\gamma _{2,1}$	0.200	0.199	0.141	0.154	0.174	0.173	0.046	0.056	0.047	0.054	0.056
$\gamma _{0,2}$	1.000	0.989	1.064	0.984	0.985	0.985	0.217	0.204	0.218	0.217	0.217
$\gamma _{1,2}$	0.200	0.201	0.181	0.196	0.199	0.199	0.032	0.034	0.033	0.034	0.034
$\gamma _{2,2}$	$-$ 0.200	$-$ 0.199	$-$ 0.178	$-$ 0.186	$-$ 0.189	$-$ 0.194	0.037	0.038	0.037	0.037	0.038
Conditional variances											
$\sigma _1^2$	0.640	0.638	0.459	0.649	0.644	0.644	0.028	0.029	0.029	0.029	0.029
$\sigma _2^2$	0.360	0.360	0.319	0.362	0.361	0.361	0.017	0.018	0.018	0.018	0.018
$\upsilon _1^2$	0.810	0.641	0.372	0.645	0.644	0.644	0.303	0.180	0.305	0.305	0.305
$\upsilon _2^2$	0.490	0.446	0.385	0.446	0.446	0.446	0.211	0.182	0.211	0.210	0.210

		RMSE					Coverage				
		BD	CC	IBM	DART	DART-m	BD	CC	IBM	DART	DART-m
Regression coefficient											
$\gamma _{0,1}$		0.302	0.550	0.304	0.303	0.301	0.888	0.330	0.890	0.891	0.890
$\gamma _{1,1}$		0.045	0.124	0.091	0.074	0.072	0.948	0.439	0.659	0.874	0.895
$\gamma _{2,1}$		0.045	0.080	0.079	0.077	0.076	0.953	0.830	0.753	0.828	0.851
$\gamma _{0,2}$		0.222	0.213	0.222	0.225	0.222	0.931	0.925	0.923	0.916	0.926
$\gamma _{1,2}$		0.034	0.039	0.036	0.035	0.036	0.944	0.910	0.936	0.942	0.941
$\gamma _{2,2}$		0.037	0.045	0.044	0.043	0.042	0.948	0.906	0.915	0.912	0.916
Conditional variances											
$\sigma _1^2$		0.029	0.184	0.031	0.030	0.029	0.945	0.000	0.936	0.944	0.945
$\sigma _2^2$		0.018	0.045	0.018	0.018	0.018	0.944	0.401	0.944	0.943	0.942
$\upsilon _1^2$		0.295	0.455	0.295	0.294	0.294	0.917	0.504	0.920	0.922	0.922
$\upsilon _2^2$		0.147	0.153	0.147	0.147	0.147	0.986	0.984	0.987	0.989	0.986

$G=2;\ C=20;\ N=4000;\ J=20;\ n_{\textrm{iter}}=20{,}000+5000$. RMSE = root mean square error; BD = before deletion; CC = complete cases; IBM = multiple imputation before modeling based on observed data; DART = data augmentation using sequential recursive partitioning based on all data and latent parameters. DART-m = data augmentation using sequential recursive partitioning based on the sufficient statistics $\theta$ and $\omega$. Runtimes = mean runtimes per data set in minutes (Leibniz Supercomputing Centre of the Bavarian Academy of Sciences and Humanities).

**Table 4:** Simulation study (scenario 2, missing rates: $X_1=40\%$, $X_2=50\%$, overall = 59%)—True parameter values, mean posterior medians and standard deviations, RMSEs and coverage ratios of structural parameter (regression coefficients, variance parameters) over 1000 replications obtained from BD, CC, IBM and DART.

	True	Average					Averaged standard deviation				
		BD	CC	IBM	DART	DART-m	BD	CC	IBM	DART	DART-m
Runtimes [min]		92	46	364	381	286					
Regression coefficient											
$\gamma _{0,1}$	$-$ 0.500	$-$ 0.505	0.453	$-$ 0.526	$-$ 0.523	$-$ 0.523	0.260	0.199	0.261	0.262	0.266
$\gamma _{1,1}$	0.400	0.398	0.252	0.285	0.309	0.323	0.044	0.076	0.053	0.077	0.085
$\gamma _{2,1}$	0.200	0.198	0.120	0.123	0.137	0.137	0.046	0.081	0.050	0.058	0.062
$\gamma _{0,2}$	1.000	0.988	1.217	0.975	0.977	0.986	0.217	0.193	0.218	0.218	0.218
$\gamma _{1,2}$	0.200	0.201	0.162	0.183	0.189	0.191	0.032	0.041	0.034	0.037	0.037
$\gamma _{2,2}$	$-$ 0.200	$-$ 0.198	$-$ 0.160	$-$ 0.164	$-$ 0.172	$-$ 0.187	0.037	0.045	0.037	0.039	0.040
Conditional variances											
$\sigma _1^2$	0.640	0.639	0.400	0.655	0.650	0.647	0.028	0.041	0.029	0.030	0.030
$\sigma _2^2$	0.360	0.360	0.287	0.364	0.363	0.362	0.017	0.021	0.018	0.018	0.018
$\upsilon _1^2$	0.810	0.643	0.291	0.648	0.646	0.647	0.304	0.147	0.307	0.305	0.307
$\upsilon _2^2$	0.490	0.444	0.331	0.445	0.445	0.445	0.210	0.158	0.211	0.210	0.210

		RMSE					Coverage				
		BD	CC	IBM	DART	DART-m	BD	CC	IBM	DART	DART-m
Regression coefficient											
$\gamma _{0,1}$		0.302	0.970	0.308	0.309	0.312	0.891	0.003	0.880	0.888	0.889
$\gamma _{1,1}$		0.044	0.168	0.140	0.127	0.121	0.950	0.501	0.448	0.742	0.833
$\gamma _{2,1}$		0.045	0.113	0.121	0.117	0.110	0.954	0.839	0.566	0.665	0.717
$\gamma _{0,2}$		0.222	0.283	0.225	0.226	0.224	0.932	0.819	0.928	0.918	0.926
$\gamma _{1,2}$		0.034	0.056	0.043	0.042	0.042	0.944	0.844	0.885	0.914	0.920
$\gamma _{2,2}$		0.037	0.060	0.059	0.056	0.051	0.948	0.855	0.787	0.839	0.872
Conditional variances											
$\sigma _1^2$		0.029	0.244	0.034	0.032	0.032	0.945	0.004	0.901	0.926	0.928
$\sigma _2^2$		0.017	0.076	0.018	0.018	0.018	0.946	0.116	0.942	0.938	0.938
$\upsilon _1^2$		0.295	0.525	0.293	0.294	0.293	0.917	0.222	0.923	0.922	0.923
$\upsilon _2^2$		0.146	0.183	0.146	0.146	0.146	0.986	0.951	0.987	0.987	0.988

$G=2;\ C=20;\ N=4000;\ J=20;\ n_{\textrm{iter}}=20{,}000+5000$. RMSE = root mean square error; BD = before deletion; CC = complete cases; IBM = multiple imputation before modeling based on observed data; DART = data augmentation using sequential recursive partitioning based on all data and latent parameters. DART-m = data augmentation using sequential recursive partitioning based on the sufficient statistics $\theta$ and $\omega$. Runtimes = mean runtimes per data set in minutes (Leibniz Supercomputing Centre of the Bavarian Academy of Sciences and Humanities).

**Table 5:** Simulation study (scenario 3, missing rates: $X_1=20\%$, $X_2=36\%$, overall = 46%)—True parameter values, mean posterior medians and standard deviations, RMSEs and coverage ratios of structural parameter (regression coefficients, variance parameters) over 1000 replications obtained from BD, CC, IBM and DART.

	True	Average					Averaged standard deviation				
		BD	CC	IBM	DART	DART-m	BD	CC	IBM	DART	DART-m
Runtimes [min]		94	55	344	366	239					
Regression coefficient											
$\gamma _{0,1}$	$-$ 0.500	$-$ 0.507	$-$ 0.642	$-$ 0.514	$-$ 0.513	$-$ 0.505	0.259	0.254	0.260	0.261	0.261
$\gamma _{1,1}$	0.400	0.398	0.370	0.385	0.392	0.393	0.044	0.054	0.045	0.046	0.046
$\gamma _{2,1}$	0.200	0.198	0.176	0.180	0.187	0.196	0.046	0.056	0.046	0.048	0.048
$\gamma _{0,2}$	1.000	0.989	0.856	0.980	0.987	0.991	0.218	0.217	0.218	0.219	0.217
$\gamma _{1,2}$	0.200	0.201	0.185	0.175	0.190	0.192	0.032	0.049	0.034	0.037	0.037
$\gamma _{2,2}$	$-$ 0.200	$-$ 0.198	$-$ 0.201	$-$ 0.160	$-$ 0.185	$-$ 0.194	0.037	0.055	0.036	0.040	0.041
Conditional variances											
$\sigma _1^2$	0.640	0.639	0.606	0.642	0.640	0.639	0.028	0.034	0.028	0.028	0.028
$\sigma _2^2$	0.360	0.360	0.346	0.365	0.362	0.361	0.017	0.025	0.018	0.018	0.018
$\upsilon _1^2$	0.810	0.642	0.594	0.643	0.644	0.643	0.303	0.282	0.304	0.304	0.305
$\upsilon _2^2$	0.490	0.444	0.418	0.445	0.445	0.445	0.209	0.199	0.210	0.210	0.209

		RMSE					Coverage				
		BD	CC	IBM	DART	DART-m	BD	CC	IBM	DART	DART-m
Regression coefficient											
$\gamma _{0,1}$		0.302	0.318	0.301	0.299	0.301	0.897	0.863	0.895	0.910	0.890
$\gamma _{1,1}$		0.044	0.062	0.049	0.047	0.047	0.950	0.915	0.936	0.950	0.951
$\gamma _{2,1}$		0.045	0.061	0.057	0.056	0.055	0.956	0.934	0.886	0.907	0.911
$\gamma _{0,2}$		0.223	0.256	0.223	0.224	0.222	0.932	0.891	0.937	0.928	0.931
$\gamma _{1,2}$		0.034	0.050	0.045	0.040	0.039	0.947	0.946	0.864	0.935	0.946
$\gamma _{2,2}$		0.037	0.056	0.060	0.051	0.051	0.950	0.948	0.750	0.870	0.884
Conditional variances											
$\sigma _1^2$		0.029	0.048	0.029	0.029	0.029	0.945	0.841	0.935	0.940	0.943
$\sigma _2^2$		0.018	0.029	0.018	0.018	0.018	0.945	0.903	0.937	0.945	0.941
$\upsilon _1^2$		0.294	0.309	0.295	0.295	0.295	0.918	0.898	0.919	0.920	0.921
$\upsilon _2^2$		0.145	0.146	0.146	0.146	0.146	0.988	0.985	0.989	0.989	0.989

$G=2;\ C=20;\ N=4000;\ J=20;\ n_{\textrm{iter}}=20{,}000+5000$. RMSE = root mean square error; BD = before deletion; CC = complete cases; IBM = multiple imputation before modeling based on observed data; DART = data augmentation using sequential recursive partitioning based on all data and latent parameters. DART-m = data augmentation using sequential recursive partitioning based on the sufficient statistics $\theta$ and $\omega$. Runtimes = mean runtimes per data set in minutes (Leibniz Supercomputing Centre of the Bavarian Academy of Sciences and Humanities).

**Table 6:** Simulation study (scenario 4, missing rates: $X_1=17\%$, $X_2=28\%$, overall $=\,40\%$)—True parameter values, mean posterior medians and standard deviations, RMSEs and coverage ratios of structural parameter (regression coefficients, variance parameters) over 1000 replications obtained from BD, CC, IBM and DART.

	True	Average					Averaged standard deviation				
		BD	CC	IBM	DART	DART-m	BD	CC	IBM	DART	DART-m
Runtimes [min]		93	60	340	363	227					
Regression coefficient											
$\gamma _{0,1}$	$-$ 0.500	$-$ 0.508	$-$ 0.491	$-$ 0.512	$-$ 0.507	$-$ 0.507	0.262	0.258	0.262	0.260	0.262
$\gamma _{1,1}$	0.400	0.398	0.388	0.378	0.390	0.392	0.044	0.055	0.045	0.047	0.047
$\gamma _{2,1}$	0.200	0.198	0.193	0.179	0.191	0.196	0.046	0.058	0.046	0.048	0.049
$\gamma _{0,2}$	1.000	0.988	0.966	0.980	0.984	0.988	0.217	0.218	0.218	0.219	0.218
$\gamma _{1,2}$	0.200	0.201	0.199	0.189	0.197	0.198	0.032	0.042	0.033	0.035	0.035
$\gamma _{2,2}$	$-$ 0.200	$-$ 0.198	$-$ 0.192	$-$ 0.174	$-$ 0.189	$-$ 0.195	0.037	0.047	0.036	0.038	0.039
Conditional variances											
$\sigma _1^2$	0.640	0.639	0.618	0.643	0.640	0.639	0.028	0.035	0.028	0.028	0.028
$\sigma _2^2$	0.360	0.360	0.355	0.363	0.361	0.361	0.017	0.022	0.018	0.018	0.018
$\upsilon _1^2$	0.810	0.643	0.612	0.643	0.643	0.643	0.304	0.291	0.305	0.303	0.304
$\upsilon _2^2$	0.490	0.444	0.435	0.445	0.445	0.445	0.210	0.206	0.210	0.210	0.210

		RMSE					Coverage				
		BD	CC	IBM	DART	DART-m	BD	CC	IBM	DART	DART-m
Regression coefficient											
$\gamma _{0,1}$		0.299	0.292	0.302	0.299	0.301	0.898	0.908	0.895	0.889	0.889
$\gamma _{1,1}$		0.044	0.058	0.052	0.049	0.048	0.951	0.943	0.915	0.942	0.944
$\gamma _{2,1}$		0.045	0.057	0.054	0.053	0.053	0.954	0.959	0.898	0.924	0.931
$\gamma _{0,2}$		0.221	0.223	0.223	0.223	0.224	0.929	0.934	0.931	0.929	0.930
$\gamma _{1,2}$		0.034	0.042	0.037	0.036	0.036	0.945	0.945	0.933	0.943	0.946
$\gamma _{2,2}$		0.037	0.047	0.048	0.043	0.043	0.953	0.956	0.858	0.909	0.926
Conditional variances											
$\sigma _1^2$		0.029	0.042	0.029	0.029	0.029	0.944	0.889	0.938	0.945	0.943
$\sigma _2^2$		0.018	0.023	0.018	0.018	0.018	0.945	0.947	0.942	0.946	0.946
$\upsilon _1^2$		0.295	0.304	0.295	0.295	0.295	0.918	0.899	0.920	0.917	0.920
$\upsilon _2^2$		0.146	0.146	0.146	0.146	0.146	0.988	0.988	0.985	0.989	0.989

$G=2;\ C=20;\ N=4000;\ J=20;\ n_{\textrm{iter}}=20{,}000+5000$. RMSE = root mean square error; BD = before deletion; CC = complete cases; IBM = multiple imputation before modeling based on observed data; DART = data augmentation using sequential recursive partitioning based on all data and latent parameters. DART-m = data augmentation using sequential recursive partitioning based on the sufficient statistics $\theta$ and $\omega$. Runtimes = mean runtimes per data set in minutes (Leibniz Supercomputing Centre of the Bavarian Academy of Sciences and Humanities).

Each of the repeated estimations is finally based on MCMC chains of length 25,000. After discarding the first 5000 iterations as burn-in, inference is based on the remaining 20,000 simulated draws from the joint posterior distribution. Convergence is monitored via the Geweke statistic, the Gelman–Rubin statistics, and the effective sample size, see Geweke (1992), Gelman et al. (2013), and Vehtari et al. (2021), and the supplementary material for further information. The convergence diagnostics indicate overall convergence.

Results for the four different missing scenarios are presented in Tables 3, 4, 5 and 6. They provide the true parameter values used in the DGP, mean posterior medians and averaged standard deviations over the 1, 000 replications obtained for the BD, CC, IBM, DART, and DART-m sample estimates with regard to the regression coefficients and conditional variance parameters. Beside the averaged estimates, simulation results are also evaluated in terms of the root mean square error (RMSE) and the coverage, i.e. the proportion of $95\%$ highest posterior density regions (HDRs) that contain the true DGP parameter values. For completeness, results on item characteristics (item discrimination, item difficulty and item category cutoff parameters) are available in the supplementary material. For the BD estimates we find overall unbiased results for all parameters. The results indicate a correct implementation of the algorithm and further serve as a benchmark to assess the relative performance of the different methods in the case of missing values. As expected, the CC results show a huge bias, where the bias becomes larger as the proportion of missing values increases. The results also show that the biases tend to be larger when the probability of missing values in $X^{(2)}$ and $X^{(3)}$ depends only on $\theta$, see Tables 3 and 4, and not additionally on the covariates themselves, see Tables 5 and 6. Not unexpectedly, coverage rates for CC are the lowest, see e.g. the parameters $\gamma _{0,1}$ and $\sigma _1^2$ in Table 3.

When comparing IBM to DART and DART-m, the differences are less pronounced. Nevertheless, it appears consistently across all four simulation studies that with using DART or DART-m we achieve smaller biases. Further inspection of the RMSE for IBM, DART and DART-m suggests no severe loss of statistical efficiency compared to BD, but with a small advantage for DART and DART-m. These results are supported by the coverage rates meeting the $95\%$ confidence level for most of the parameters using DART, especially DART-m, whereas this becomes especially clear with Scenario 2 in Table  4 showing the highest proportion of missing values. Here, we could only achieve a coverage rate of around $50\%$ for the parameters $\gamma _{1,1}$ and $\gamma _{2,1}$ using IBM, but obtain higher coverage rates using DART and even better using DART-m.

Taking a look at the averaged standard deviations, these tend to be smaller for IBM, since without the latent variables $\theta$ and $\omega$ drawn from the full conditional distributions in each iteration, we do not consider an important source of variability affecting the uncertainty of the missing values. Further, without consideration of $\theta$ and $\omega$, the bias increases as shown by our simulation results.

The advantages of the DART-m approach are particularly evident in the runtimes (mean runtimes per data set in minutes) given in Tables 3, 4, 5 and 6. DART-m efficiently uses the information from the latent variables $\theta$ and $\omega$, which serve as sufficient statistics and therefore can replace the item response *Y* and the school affiliation *S*. The resulting runtimes show that the suggested DART-m approach saves up to one third of the computation time compared to the IBM approach.

Similar effects can be seen when inspecting the properties of the sampled trajectories $\{X_{\text {mis}}^{(r)}\}_{r=1}^R$. The properties arising from the different approaches can be assessed via calculating for each missing value the absolute and squared distance to the true (before deletion) and estimated value. With the former providing bias and the latter the variance, we summarize the finding per variable and aggregate over the missing values per variable and over the simulated data sets. The same procedure is also done to obtain root mean square errors. Note that after averaging over missing values per variable and over data sets, root mean square errors are not exactly identical to variance plus squared bias. With regard to bias and variance we calculate bias as $\frac{1}{{\mathcal {S}}} \sum _{{\mathcal {s}}=1}^{{\mathcal {S}}} \frac{1}{\# X_{\text {mis}}^{(j)}}\sum _{k=1}^{\# X_{\text {mis}}^{(j)}}|X_{\text {mis},k,{\mathcal {s}}}^{(j)}-{\tilde{X}}_{\text {mis},k,{\mathcal {s}}}^{(j)}|$, variance as $\frac{1}{{\mathcal {S}}} \sum _{{\mathcal {s}}=1}^{{\mathcal {S}}} \frac{1}{\# X_{\text {mis}}^{(j)}}\sum _{k=1}^{\# X_{\text {mis}}^{(j)}}(X_{\text {mis},k,{\mathcal {s}}}^{(j)}-{\hat{X}}_{\text {mis},k,{\mathcal {s}}}^{(j)})^2$, and root mean square error as $\frac{1}{{\mathcal {S}}} \sum _{{\mathcal {s}}=1}^{{\mathcal {S}}} \frac{1}{\# X_{\text {mis}}^{(j)}}\sum _{k=1}^{\# X_{\text {mis}}^{(j)}}\sqrt{(X_{\text {mis},k,{\mathcal {s}}}^{(j)}-{\tilde{X}}_{\text {mis},k,{\mathcal {s}}}^{(j)})^2}$. Thereby, $\# X_{\text {mis}}^{(j)}$ denotes the number of missing values per variable, $X_{\text {mis},k,{\mathcal {s}}}^{(j)}$ the *k*th missing value in variable $j=2,3$, and ${\tilde{X}}_{\text {mis},k,{\mathcal {s}}}^{(j)}$ and ${\hat{X}}_{\text {mis},k,{\mathcal {s}}}^{(j)}$ denote true (before deletion) and estimated values within repeated estimation ${\mathcal {s}}$ respectively. The results are described in Table 7. As expected and in line with the other simulation results presented, the suggested augmentation approaches DART and DART-m show reduced bias although slightly increased variance compared to the IBM approach. This in turn then causes the improved inference regarding the regression coefficients both in terms of bias and coverage.

**Table 7:** Comparison of prediction accuracy of conditioning variables *X*.

	Scenario I		Scenario II		Scenario III		Scenario IV	
	$X^{(2)}$	$X^{(3)}$	$X^{(2)}$	$X^{(3)}$	$X^{(2)}$	$X^{(3)}$	$X^{(2)}$	$X^{(3)}$
*Root mean square error*								
IBM	0.5784	0.5689	0.5992	0.5793	0.6390	0.5728	0.6157	0.5728
DART	0.6014	0.5790	0.6240	0.5914	0.6660	0.5808	0.6436	0.5811
DART-m	0.6020	0.5837	0.6245	0.6012	0.6669	0.5825	0.6438	0.5826
*Bias*								
IBM	0.3951	0.3790	0.4207	0.3924	0.4613	0.3766	0.4356	0.3750
DART	0.3913	0.3606	0.4182	0.3769	0.4596	0.3573	0.4351	0.3563
DART-m	0.3905	0.3520	0.4162	0.3661	0.4595	0.3483	0.4347	0.3476
*Variance*								
IBM	0.1534	0.1459	0.1542	0.1466	0.1684	0.1512	0.1628	0.1523
DART	0.1903	0.1697	0.1975	0.1715	0.2203	0.1734	0.2112	0.1743
DART-m	0.1924	0.1814	0.2007	0.1921	0.2226	0.1820	0.2119	0.1824

Quantities are calculated as follows with $\text {bias}=\frac{1}{\mathcal {S}} \sum _{\mathcal {s}=1}^{\mathcal {S}} \frac{1}{\# X_{\textrm{mis}}^{(j)}}\sum _{k=1}^{\# X_{\textrm{mis}}^{(j)}}|X_{\textrm{mis},k,\mathcal {s}}^{(j)}-\tilde{X}_{\textrm{mis},k,\mathcal {s}}^{(j)}|$, $j=2,3$, $\text {variance}=\frac{1}{\mathcal {S}} \sum _{\mathcal {s}=1}^{\mathcal {S}} \frac{1}{\# X_{\textrm{mis}}^{(j)}}\sum _{k=1}^{\# X_{\textrm{mis}}^{(j)}}(X_{\textrm{mis},k,\mathcal {s}}^{(j)}-\hat{X}_{\textrm{mis},k,\mathcal {s}}^{(j)})^2$, $j=2,3$, and $\text {root mean square error}=\frac{1}{\mathcal {S}} \sum _{\mathcal {s}=1}^{\mathcal {S}} \frac{1}{\# X_{\textrm{mis}}^{(j)}}\sum _{k=1}^{\# X_{\textrm{mis}}^{(j)}}\sqrt{(X_{\textrm{mis},k,\mathcal {s}}^{(j)}-\tilde{X}_{\textrm{mis},k,\mathcal {s}}^{(j)})^2}$, $j=2,3$, with $\# X_{\textrm{mis}}^{(j)}$ denoting the number of missing values per variable, $X_{\textrm{mis},k,\mathcal {s}}^{(j)}$ the *k*th missing value in variable *j*, and $\tilde{X}_{\textrm{mis},k,\mathcal {s}}^{(j)}$ and $\hat{X}_{\textrm{mis},k,\mathcal {s}}^{(j)}$ denote true (before deletion) and estimated values in repeated estimation $\mathcal {s}$ respectively.

To summarize, the simulation illustrates that the combination of data augmentation and sequential recursive partitioning offers a suitable solution for the treatment of missing covariates in the context of LRMs, both with regard to estimation efficiency and computational burden.

## Empirical Illustration

In order to illustrate the usefulness of the suggested Bayesian data augmentation approach in empirical analysis, we provide exemplary applications using the scientific data use file of the German National Educational Panel Study: Starting Cohort Grade 9, doi: 10.5157/NEPS:SC4:10.0.0, see NEPS Network (2019), on mathematical competencies of ninth graders. Children of this cohort have been surveyed in an institutional context. Data collection has taken place in schools in Germany between fall 2010 and winter 2010/2011 based on a stratified sampling of schools according to school types, see Aßmann et al. (2011). Both factors, the institutional setting of schools in Germany as well as the stratified sampling approach, give reason to consider a differentiated hierarchical data structure.

We chose the mathematical competency domain as an example for latent variable modeling with person covariates. The relationship between mathematical competency and individual characteristics is thereby structured by the type of secondary schooling. Mathematical competency was assessed in the first survey wave. The corresponding test comprises four content areas: *quantity*, *change and relationships*, *space and shape*, and *data and chance* (Neumann et al., 2013), where a total of 15, 629 ninth graders have taken the considered test. For an overview and further results on the mathematics test data see Duchhardt and Gerdes (2013). As most of the items have low missing rates, the estimation within the empirical illustration is based on the likelihood involving observed values of *Y* only and only students with a valid response to at least three mathematics test items are consider.^18^ From the $J=22$ tasks that had to be solved in the test, 20 items have a binary format and two are treated as ordinal items with four categories. In addition to the test data, we consider two clustering variables (*schooltype* and *school*) and student covariates. Merging mathematics test data and all student information together results in a final data set with 14, 320 observations. The available school type variable (Bayer et al., 2014) was transformed to cover four tracks of the German secondary education system: Hauptschule (*HS*; lower track), Realschule (*RS*; intermediate track), Gymnasium (*GYM*; academic or upper track) and, for observations where a clear assignment to these tracks was not possible or unclear, we define a residual category (*OTHER*). With $37\%$ of students, *GYM* is the modal track. The school identifier *school* assigns a unique number to each school and serves as a further clustering variable with a total of 532 schools. Table 8 provides the descriptive statistics on the sample and considered variables. The illustration is provided in form of the following two model specifications.

**Table 8:** NEPS grade 9—descriptive statistics (complete case summary).

	ALL	HS	RS	GYM	OTHER
Students	14320	3755 (26.2%)	4301 (30.0%)	5380 (37.6%)	884 (6.2%)
Students$_{CC}$—model 1	6748	1320 (19.6%)	1901 (28.2%)	3126 (46.3%)	401 (5.9%)
Missing$_{CC}$—model 1	52.8%	64.7%	55.8%	41.9%	54.6%
Students$_{CC}$—model 2	7708	1617 (21.0%)	2198 (28.5%)	3496 (45.4%)	397 (5.2%)
Missing$_{CC}$—model 2	46.2%	56.9%	48.9%	35.0%	55.1%
Schools	532	187 (35.2%)	152 (28.6%)	159 (29.9%)	34 (6.4%)
*Person covariate: Gender*					
0: Male	7164 (50.1%)	2085 (55.6%)	2187 (51.0%)	2448 (45.6%)	444 (50.4%)
1: Female	7126 (49.9%)	1662 (44.4%)	2101 (49.0%)	2926 (54.4%)	437 (49.6%)
Missing	30 (0.2%)	8 (0.2%)	13 (0.3%)	6 (0.1%)	3 (0.3%)
*Person covariate: Generation status*					
0: No migrant background	10152 (70.9%)	2201 (58.6%)	3244 (75.5%)	4150 (77.2%)	557 (63.0%)
1: 1st generation	900 (6.3%)	431 (11.5%)	229 (5.3%)	177 (3.3%)	63 (7.1%)
2: 2nd generation	1891 (13.2%)	794 (21.2%)	451 (10.5%)	497 (9.2%)	149 (16.9%)
3: 3rd generation	1370 (9.6%)	327 (8.7%)	374 (8.7%)	554 (10.3%)	115 (13.0%)
Missing	7 (0.04%)	2 (0.05%)	3 (0.07%)	2 (0.04%)	0 (0.00%)
*Person covariate: Grade final report card - mathematics*					
1: Very good	905 (6.6%)	145 (4.1%)	196 (4.8%)	496 (9.4%)	68 (9.2%)
2: Good	3545 (26.0%)	814 (22.9%)	999 (24.4%)	1533 (29.1%)	199 (26.8%)
3: Satisfactory	4993 (36.6%)	1342 (37.7%)	1539 (37.7%)	1846 (35.0%)	266 (35.8%)
4: Passing	3328 (24.4%)	921 (25.9%)	1074 (26.3%)	1169 (22.2%)	164 (22.1%)
5: Poor	853 (6.2%)	320 (9.0%)	268 (6.6%)	221 (4.2%)	44 (5.9%)
6: Failing	36 (0.3%)	18 (0.5%)	10 (0.2%)	7 (0.1%)	1 (0.1%)
Missing	660 (4.6%)	195 (5.2%)	215 (5.0%)	108 (2.0%)	142 (16.1%)
*Person covariate: School year repeated*					
0: Yes	2674 (19.2%)	1224 (34.1%)	904 (21.6%)	444 (8.4%)	102 (12.2%)
1: No	11249 (80.8%)	2370 (65.9%)	3273 (78.4%)	4873 (91.6%)	733 (87.8%)
Missing	397 (2.8%)	161 (4.3%)	124 (2.9%)	63 (1.2%)	49 (5.5%)
*Person covariate: Is there a computer you can use in your house?*					
1: Yes, own	10246 (72.5%)	2549 (69.1%)	3113 (73.3%)	3987 (74.8%)	597 (69.1%)
2: Yes, shared	3778 (26.7%)	1093 (29.6%)	1105 (26.0%)	1322 (24.8%)	258 (29.9%)
3: No	107 (0.8%)	49 (1.3%)	30 (0.7%)	19 (0.4%)	9 (1.0%)
Missing	189 (1.3%)	64 (1.7%)	53 (1.2%)	52 (1.0%)	20 (2.3%)
*Person covariate: HOMEPOS Room*					
0: Not specified	1000 (7.0%)	434 (11.7%)	274 (6.4%)	212 (4.0%)	80 (9.2%)
1: Specified	13228 (93.0%)	3285 (88.3%)	3998 (93.6%)	5152 (96.0%)	793 (90.8%)
Missing	92 (0.6%)	36 (1.0%)	29 (0.7%)	16 (0.3%)	11 (1.2%)
*Person covariate: HCASMIN*					
0: no qualification	62 (0.8%)	41 (2.3%)	7 (0.3%)	8 (0.2%)	6 (1.2%)
1: general elementary educ.	259 (3.2%)	171 (9.8%)	36 (1.5%)	31 (0.9%)	21 (4.2%)
2: basic voc. training beyond comp. educ.	994 (12.1%)	517 (29.5%)	310 (13.2%)	117 (3.2%)	50 (10.1%)
3: inter. sec. educ. without voc. qual.	276 (3.4%)	110 (6.3%)	88 (3.7%)	58 (1.6%)	20 (4.0%)
4: inter. sec. educ. with voc. qual.	2749 (33.5%)	576 (32.9%)	1082 (46.1%)	926 (25.7%)	165 (33.3%)
5: higher educ. inst. without voc. qual.	341 (4.2%)	83 (4.7%)	86 (3.7%)	151 (4.2%)	21 (4.2%)
6: higher educ. inst. with voc. qual.	1288 (15.7%)	140 (8.0%)	373 (15.9%)	702 (19.4%)	73 (14.7%)
7: university of applied sciences degree	720 (8.8%)	58 (3.3%)	164 (7.0%)	456 (12.6%)	42 (8.5%)
8: higher tertiary educ.	1514 (18.5%)	54 (3.1%)	202 (8.6%)	1161 (32.2%)	97 (19.6%)
Missing	6117 (42.7%)	2005 (53.4%)	1953 (45.4%)	1770 (32.9%)	389 (44.0%)
*Person covariate: HISEI*					
Mean	5.12	4.02	4.76	6.06	5.09
Sd	2.07	1.78	1.88	1.94	2.09
Min/Max	1.16/8.89	1.17/8.90	1.16/8.90	1.17/8.90	1.42/8.90
Missing	3060 (21.4%)	1069 (28.5%)	948 (22.0%)	796 (14.8%)	247 (27.9%)
*Person covariate: Agetest*					
Mean	15.15	15.44	15.18	14.93	15.16
Sd	0.63	0.70	0.60	0.51	0.60
Min/Max	11.17/18.67	11.17/18.67	13.08/18.50	12.25/17.92	14.00/ 17.67
Missing	16 (0.0%)	15 (0.0%)	0(0.0%)	1 (0.0%)	0 (0.0%)
*Person covariate: Schoolexp*					
Mean	8.57	8.79	8.60	8.44	8.55
Sd	0.62	0.77	0.59	0.53	0.59
Min/Max	6.17/15.33	6.25/14.17	6.25/11.25	6.17/15.33	6.17/12.33
Missing	6184 (42.9%)	2045 (54.4%)	1924(44.7%)	1744 (32.4%)	471 (53.3%)

Absolute and relative counts (in parentheses) are reported.

*educ.* education, *comp.* compulsory, *sec.* secondary, *inter.* intermediate, *voc.* vocational, *qual.* qualification, *inst.* institution.

**Table 9:** NEPS grade 9, mathematical competencies—parameter estimates of model I.

	HS		RS		GYM		OTHER	
$\gamma _{g,\textrm{Intercept}}$	0.834*	(0.441)	1.887***	(0.525)	1.831**	(0.756)	0.973	(1.166)
$\gamma _{g,\textrm{Gender}:1}$	$-$ 0.215***	(0.015)	$-$ 0.313***	(0.015)	$-$0.313***	(0.016)	$-$ 0.268***	(0.033)
$\gamma _{g,\textrm{HISEI}}$	$-$ 0.018	(0.102)	$-$ 0.118	(0.104)	0.212*	(0.118)	0.134	(0.222)
$\gamma _{g,\textrm{Age}}$	$-$ 0.054	(0.038)	$-$ 0.112**	(0.045)	$-$ 0.070	(0.060)	$-$ 0.056	(0.095)
$\gamma _{g,\textrm{Experience}}$	$-$ 0.030	(0.035)	$-$ 0.010	(0.045)	$-$ 0.017	(0.060)	$-$ 0.023	(0.094)
$\gamma _{g,\textrm{HISEI} \times \textrm{Age}}$	$-$ 0.003	(0.009)	0.007	(0.009)	$-$ 0.010	(0.009)	$-$ 0.008	(0.018)
$\gamma _{g,\textrm{HISEI} \times \textrm{Experience}}$	0.008	(0.008)	0.004	(0.009)	$-$ 0.004	(0.010)	0.002	(0.018)
$\sigma _{g}^2$	0.104	(0.005)	0.138	(0.005)	0.226	(0.007)	0.153	(0.012)
$\upsilon _{g}^2$	0.048	(0.006)	0.06	(0.008)	0.091	(0.011)	0.093	(0.025)
Within group correlation	0.686	(0.028)	0.698	(0.028)	0.713	(0.026)	0.622	(0.060)

$C=532;\ N=14320;\ N_{CC}=6748;\ J=22$. Median and standard deviation (in parentheses) of the posterior distribution are reported.

*$90\%$ HDI; **$95\%$ HDI; ***$99\%$ HDI. Runtime: 35.6 h.

**Table 10:** NEPS grade 9, mathematical competencies—relative effects for structural parameter estimates of model I.

$\gamma _{g}-\gamma _{g'}$	HS–GYM		RS–GYM		OTHER–GYM	
$\gamma _{g}-\gamma _{g'},\textrm{Intercept}$	$-$ 0.988	(0.876)	0.069	(0.923)	$-$ 0.855	(1.398)
$\gamma _{g}-\gamma _{g'},\textrm{Gender}:1$	0.097***	(0.022)	$-$ 0.001	(0.022)	0.044	(0.037)
$\gamma _{g}-\gamma _{g'},\textrm{HISEI}$	$-$ 0.231	(0.156)	$-$0.332**	(0.158)	$-$ 0.078	(0.253)
$\gamma _{g}-\gamma _{g'},\textrm{Age}$	0.017	(0.071)	$-$ 0.042	(0.075)	0.012	(0.112)
$\gamma _{g}-\gamma _{g'},\textrm{Experience}$	$-$ 0.013	(0.070)	0.008	(0.075)	$-$0.006	(0.112)
$\gamma _{g}-\gamma _{g'},\textrm{HISEI} \times \textrm{Age}$	0.007	(0.013)	0.017	(0.013)	0.002	(0.020)
$\gamma _{g}-\gamma _{g'},\textrm{HISEI} \times \textrm{Experience}$	0.011	(0.012)	0.007	(0.013)	0.006	(0.020)

$C=532;\ N=14320;\ N_{CC}=6748;\ J=22$. Median and standard deviation (in parentheses) of the posterior distribution are reported.

*$90\%$ HDI; **$95\%$ HDI; ***$99\%$ HDI.

**Table 11:** NEPS grade 9, mathematical competencies—relative effects for structural parameter estimates of model II.

$\gamma _{g}-\gamma _{g'}$	HS–GYM		RS–GYM		OTHER–GYM	
$\gamma _{g}-\gamma _{g'},\textrm{Intercept}$	$-$ 1.452***	(0.145)	$-$ 0.868***	(0.184)	$-$ 0.860***	(0.217)
$\gamma _{g}-\gamma _{g'},\textrm{Gender}:1$	0.133***	(0.021)	0.033*	(0.020)	0.103***	(0.035)
$\gamma _{g}-\gamma _{g'},\textrm{GenerationStatus}:1$	0.185***	(0.050)	0.157***	(0.055)	0.071	(0.082)
$\gamma _{g}-\gamma _{g'},\textrm{GenerationStatus}:2$	0.068*	(0.038)	0.048	(0.041)	$-$ 0.036	(0.063)
$\gamma _{g}-\gamma _{g'},\textrm{GenerationStatus}:3$	0.072**	(0.034)	0.022	(0.034)	$-$ 0.037	(0.054)
$\gamma _{g}-\gamma _{g'},\textrm{GradeMathematics}:2$	0.339***	(0.047)	0.170***	(0.044)	0.195***	(0.069)
$\gamma _{g}-\gamma _{g'},\textrm{GradeMathematics}:3$	0.468***	(0.046)	0.238***	(0.044)	0.314***	(0.067)
$\gamma _{g}-\gamma _{g'},\textrm{GradeMathematics}:4$	0.529***	(0.048)	0.307***	(0.045)	0.375***	(0.072)
$\gamma _{g}-\gamma _{g'},\textrm{GradeMathematics}:5$	0.592***	(0.060)	0.342***	(0.060)	0.303***	(0.099)
$\gamma _{g}-\gamma _{g'},\textrm{GradeMathematics}:6$	0.412*	(0.221)	0.308	(0.237)	0.033	(0.491)
$\gamma _{g}-\gamma _{g'},\textrm{SchoolYearRepeated}:1$	$-$ 0.050*	(0.030)	$-$ 0.083***	(0.031)	$-$ 0.093	(0.058)
$\gamma _{g}-\gamma _{g'},\textrm{Computer}:2$	$-$ 0.020	(0.023)	$-$ 0.018	(0.023)	$-$ 0.074*	(0.040)
$\gamma _{g}-\gamma _{g'},\textrm{Computer}:3$	0.000	(0.134)	$-$ 0.065	(0.145)	0.117	(0.202)
$\gamma _{g}-\gamma _{g'},\textrm{Room}:1$	0.007	(0.044)	0.035	(0.048)	$-$ 0.068	(0.071)
$\gamma _{g}-\gamma _{g'},\textrm{HCASMIN}:1$	0.139	(0.147)	0.019	(0.189)	$-$ 0.017	(0.211)
$\gamma _{g}-\gamma _{g'},\textrm{HCASMIN}:2$	0.087	(0.137)	0.052	(0.176)	$-$ 0.040	(0.201)
$\gamma _{g}-\gamma _{g'},\textrm{HCASMIN}:3$	0.120	(0.142)	0.142	(0.180)	0.098	(0.208)
$\gamma _{g}-\gamma _{g'},\textrm{HCASMIN}:4$	0.126	(0.133)	0.097	(0.172)	0.010	(0.194)
$\gamma _{g}-\gamma _{g'},\textrm{HCASMIN}:5$	0.084	(0.137)	0.101	(0.176)	0.012	(0.202)
$\gamma _{g}-\gamma _{g'},\textrm{HCASMIN}:6$	0.108	(0.135)	0.034	(0.173)	$-$ 0.005	(0.198)
$\gamma _{g}-\gamma _{g'},\textrm{HCASMIN}:7$	0.180	(0.141)	0.156	(0.175)	0.051	(0.204)
$\gamma _{g}-\gamma _{g'},\textrm{HCASMIN}:8$	0.110	(0.140)	0.112	(0.175)	0.103	(0.198)

$C=532;\ N=14320;\ N_{CC}=7708;\ J=22$. Median and standard deviation (in parentheses) of the posterior distribution are reported.

*$90\%$ HDI; **$95\%$ HDI; ***$99\%$ HDI.

**Table 12:** NEPS grade 9, mathematical competencies—structural parameter estimates of model II.

	HS		RS		GYM		OTHER	
$\gamma _{g,\textrm{Intercept}}$	$-$ 0.108	(0.067)	0.476***	(0.130)	1.343***	(0.129)	0.483***	(0.178)
$\gamma _{g,\textrm{Gender}:1}$	$-$ 0.161***	(0.015)	$-$ 0.261***	(0.014)	$-$ 0.294***	(0.014)	$-$ 0.190***	(0.032)
$\gamma _{g,\textrm{GenerationStatus}:1}$	$-$ 0.066**	(0.028)	$-$ 0.094***	(0.036)	$-$ 0.251***	(0.041)	$-$ 0.180**	(0.071)
$\gamma _{g,\textrm{GenerationStatus}:2}$	$-$ 0.089***	(0.022)	$-$ 0.110***	(0.028)	$-$ 0.158***	(0.031)	$-$ 0.194***	(0.055)
$\gamma _{g,\textrm{GenerationStatus}:3}$	0.012	(0.026)	$-$ 0.038	(0.025)	$-$ 0.060***	(0.023)	$-$ 0.097**	(0.049)
$\gamma _{g,\textrm{GradeMathematics}:2}$	$-$ 0.107***	(0.038)	$-$ 0.276***	(0.035)	$-$ 0.446***	(0.028)	$-$ 0.251***	(0.062)
$\gamma _{g,\textrm{GradeMathematics}:3}$	$-$ 0.299***	(0.037)	$-$ 0.528***	(0.034)	$-$ 0.767***	(0.028)	$-$ 0.452***	(0.061)
$\gamma _{g,\textrm{GradeMathematics}:4}$	$-$ 0.420***	(0.038)	$-$ 0.643***	(0.035)	$-$ 0.950***	(0.029)	$-$ 0.576***	(0.066)
$\gamma _{g,\textrm{GradeMathematics}:5}$	$-$ 0.427***	(0.043)	$-$ 0.677***	(0.042)	$-$ 1.018***	(0.043)	$-$ 0.716***	(0.089)
$\gamma _{g,\textrm{GradeMathematics}:6}$	$-$ 0.458***	(0.106)	$-$ 0.562***	(0.141)	$-$ 0.870***	(0.193)	$-$ 0.837*	(0.451)
$\gamma _{g,\textrm{SchoolYearRepeated}:1}$	0.032**	(0.015)	0.000	(0.017)	0.082***	(0.026)	$-$ 0.011	(0.052)
$\gamma _{g,\textrm{Computer}:2}$	0.004	(0.016)	0.006	(0.016)	0.024	(0.017)	$-$ 0.049	(0.037)
$\gamma _{g,\textrm{Computer}:3}$	$-$ 0.028	(0.061)	$-$ 0.094	(0.083)	$-$ 0.028	(0.118)	0.089	(0.163)
$\gamma _{g,\textrm{Room}:1}$	0.025	(0.023)	0.053*	(0.029)	0.018	(0.038)	$-$ 0.050	(0.061)
$\gamma _{g,\textrm{HCASMIN}:1}$	0.117**	(0.054)	$-$ 0.004	(0.131)	$-$ 0.022	(0.136)	$-$ 0.037	(0.165)
$\gamma _{g,\textrm{HCASMIN}:2}$	0.113**	(0.052)	0.077	(0.122)	0.026	(0.126)	$-$ 0.010	(0.159)
$\gamma _{g,\textrm{HCASMIN}:3}$	0.114**	(0.056)	0.133	(0.125)	$-$ 0.007	(0.130)	0.092	(0.166)
$\gamma _{g,\textrm{HCASMIN}:4}$	0.133**	(0.052)	0.100	(0.122)	0.006	(0.122)	0.016	(0.155)
$\gamma _{g,\textrm{HCASMIN}:5}$	0.132**	(0.059)	0.149	(0.125)	0.048	(0.124)	0.062	(0.163)
$\gamma _{g,\textrm{HCASMIN}:6}$	0.199***	(0.057)	0.123	(0.122)	0.091	(0.122)	0.087	(0.159)
$\gamma _{g,\textrm{HCASMIN}:7}$	0.221***	(0.068)	0.196	(0.125)	0.041	(0.123)	0.093	(0.164)
$\gamma _{g,\textrm{HCASMIN}:8}$	0.182***	(0.069)	0.186	(0.124)	0.074	(0.122)	0.177	(0.160)
$\sigma _{g}^2$	0.087	(0.004)	0.108	(0.004)	0.151	(0.005)	0.122	(0.010)
$\upsilon _{g}^2$	0.044	(0.006)	0.059	(0.008)	0.081	(0.010)	0.090	(0.023)
Within group correlation	0.663	(0.029)	0.644	(0.030)	0.651	(0.029)	0.574	(0.062)

$C=532;\ N=14320;\ N_{CC}=7708;\ J=22$. Median and standard deviation (in parentheses) of the posterior distribution are reported.

*$90\%$ HDI; **$95\%$ HDI; ***$99\%$ HDI. Runtime: 62.8 h.

The first model specification considers a small set of background variables with different scales including cross terms, whereas the second model specification has an enlarged set of categorical background variables to illustrate that the suggested DART-m approach is feasible and efficient in terms of computational cost and statistical efficiency. For the first model specification (model I) we adapt a specification discussed by Passaretta and Skopek (2021) to assess the role of schools in socioeconomic inequality of learning. Following a differential exposure approach, the relationship of mathematical competency is analyzed with regard to the student variables *gender*, *parents’ socio-economic status (HISEI)*, *school exposure (schoolexp)*, and *age at time of assessment (agetest)*.^19^ In line with literature, we expect more school exposure and higher assessment age to be positively correlated with mathematical competence, whereas the (un)balancing effect of schools on competence is captured in terms of the cross terms between socioeconomic status and age of testing as well as school exposure. A positive effect for the considered cross terms would indicate that school experience accelerates competence more for students with higher socio economic status. The total amount of missing data for the variables within this model specification is to be considered as moderate to strong. Whereas the number of missing values in *gender* is negligible, about one fifth of the values are missing for *HISEI*. For *agetest* almost no missing values are present, whereas for school exposure the defining date of school entry was surveyed in the parental interview with a missing rate of 42.9%, see Table 8. The ratio of students having complete background information is $47.1\%$ which corresponds to 6, 748 observations. The second model specification (model II) considers an enlarged set of background variables and contains *gender* (binary), *generation status* (4 categories), *grade final report card mathematics* (6 categories), *school year repeated* (binary), *computer in your home* (3 categories), *homepos room* (binary), and *highest parental education qualification* (*HCASMIN*, 9 categories). We can see a substantial heterogeneity within the covariate *HCASMIN* between the school types. For example, we observe that $29.5\%$ of the students in *HS* have parents in category 2 (basic vocational training above and beyond compulsory schooling) but only $3.2\%$ of the students in *GYM*, or the other way round with category 8 (completed traditional, academically orientated university education) which have only $3.1\%$ of students in *HS*, but $32.2\%$ for *GYM*. Most of the variables have a negligible amount of missing values. However, we have over $40\%$ of missing values for the covariate *HCASMIN*, as this information has been surveyed within the parental interview. Therefore the ratio of students with complete background information drops to $57.3\%$, i.e. only 7708 complete case observations.

**Fig. 1:**
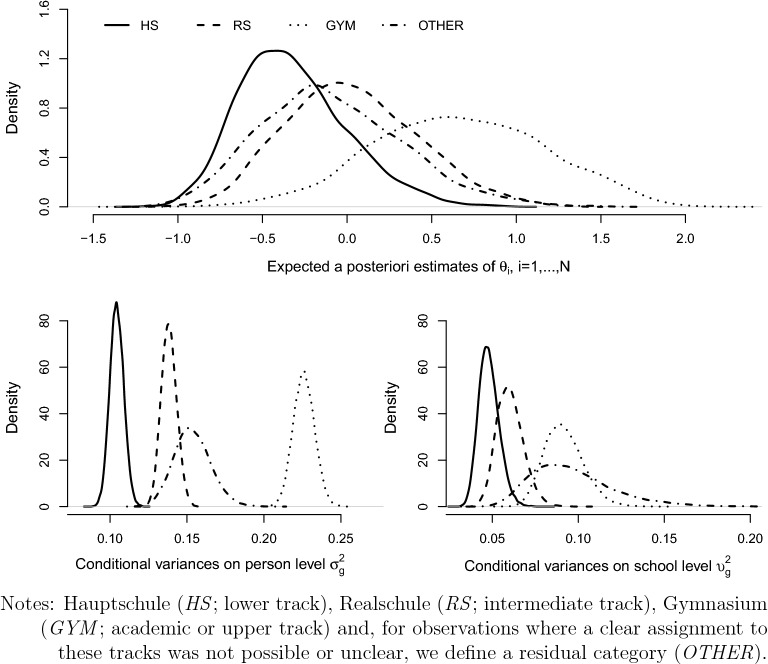
NEPS grade 9, Gaussian kernel density estimates for the set of conditional variances on person level $\sigma _g^2$ and school level $\upsilon _g^2$ and expected a posteriori estimates of scalar person parameter $\theta _i$ referring to mathematical competence in model I.

**Fig. 2:**
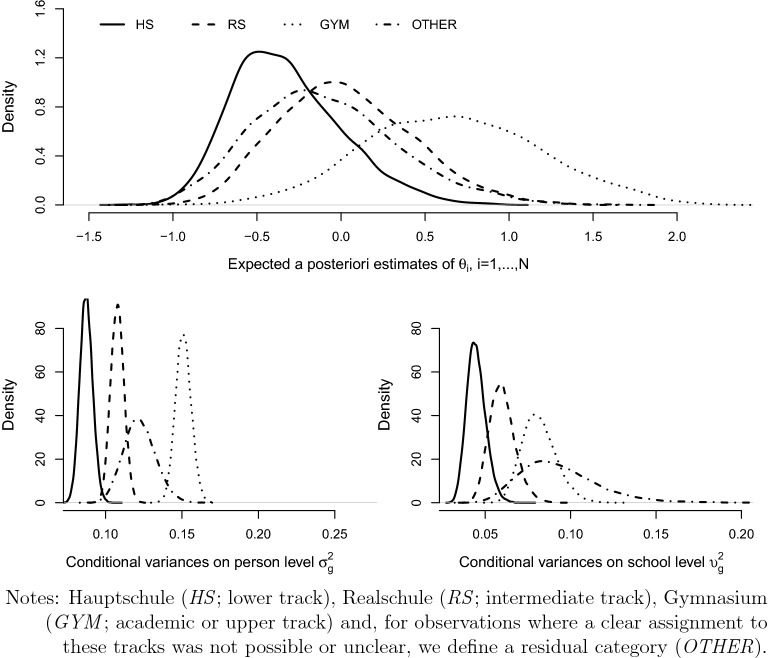
NEPS grade 9, Gaussian kernel density estimates for the set of conditional variances on person level $\sigma _g^2$ and school level $\upsilon _g^2$ and expected a posteriori estimates of scalar person parameter $\theta _i$ referring to mathematical competence in model II.

For each of the two models, estimates are based on 25,000 MCMC iterations, where a burn-in phase of 5000 has been found sufficient to mitigate the effects of initialization within the empirical analysis, see the supplementary material for corresponding results and further information concerning the convergence diagnostics and the assessment of the Monte Carlo error for the obtained point estimates.

Corresponding results for the two model specifications with regard to regression and conditional variance parameters are given in Table 9 for model I and Table 12 for model II respectively. Tables 10 and 11 provide corresponding estimates on relative effects between school types.^20^ These tables provide the resulting estimates in terms of medians, standard deviations, and highest posterior density coverage rates (HDI). The results regarding the structural relationships show clear school type specific differences in the distribution of competencies, see upper panels of Figs. 1 and 2. The highest mean scores are consistently found for *GYM*, followed by the other school types *RS*, *OTHER*, and *HS*. In the same way, the conditional variances on the person- and the school-level, $\sigma ^2_{g}$ and $\upsilon ^2_{g}$, differ across the different types of secondary schooling. However, student’s idiosyncratic error terms, i.e. inter-individual differences not captured by the covariates, constantly contribute more to the variability in mathematical competency than school belonging over the different educational tracks, see lower panels of Figs. 1 and 2.

Regarding covariate effects, the models indicate interactions with the grouping variable. For more details, let us first look at the effects of the additional personal covariates used in model I. The negative effect of being female on mathematical competency (*gender* : 1) is shown to be stable across all school types, but at varying degrees. The effects of school exposure and age at testing are completely subsumed with the school type, i.e. in ninth grade these variables have no effect beyond school type in contrast to gender. This completes the findings from the literature discussing effects in primary schools, see Passaretta and Skopek (2021).

Next, we consider the structural parameter estimates of model II. Again, we see the negative effect although slightly reduced of being female in all school types. Compared to students without a migration background, a first-generation migration background has a substantial negative (99% HDI not including zero) impact on mathematics competency across all school types. The negative effects also prevail for a migration background of the second generation, while for third generation migrants the negative effects are reduced (*GYM* and *OTHER*) or become not substantially different from zero (*HS* and *RS*). For the covariate *grade mathematics* in the previous year, where grade 1 (very good) is the reference category, we see that a good result from the previous year has a negative effect on mathematics competence compared to very good, where with worsening grades, the effect accelerates. This pattern can be observed throughout all school types, where the overall effect is strongest in the school type *GYM*. With regard to the covariate *school year repeated*, we also find differences across the school types, where this variable has no impact for school types *RS* and *OTHER*, but positively different from zero effect for school types *HS* and *GYM*. Not having your own computer, but sharing one with other family is found to have no impact on individual competence level across all school types, where we point at the possibility that this relationship may have changed since 2010 substantially. Also having an own room has no substantial effect given the considered set of covariate variables, except for school type *RS*. With regard to the variable *HCASMIN*, we find positive effects for higher *HCASMIN* levels for school type *HS*, while no effects substantially different from zero are found for all other school types. However, this variables further illustrates that the inspection of relative effects as defined in Eq. (10) with corresponding results for model specification II given in Table 11 is important to gauge differences across schools correctly. The relative effects between the different school types for the variable *HCASMIN* show no substantial differences between the school types. In this regard, the findings relate to the school specific distribution of *HCASMIN*, compare Table 8. For this model, we also calculated within group correlations, see bottom of Table 12. Although the groups show different conditional variances, estimates show no evidence for differing within group correlations.

While this effects are in line with the results from the literature, the suggested Bayesian estimation approach allows for effectively incorporating all available information, i.e. all information and model features with regard to the measurement model in terms of discrimination and difficulty parameters, intra-class correlation, and school type heterogeneity are reflected within the corresponding full conditional distributions. Given this, the results document a clear shift in means and covariate effects as well as unequal variances of the school type-specific density curves. The results of these two empirical applications extend the findings of our simulation studies from Chapter 3.

## Conclusion

To handle missing values this paper discusses a Bayesian estimation approach making use of the device of data augmentation. The missing values in conditioning variables are hence considered along with the underlying continuous outcomes, the model parameters and the latent traits or hierarchical structures in the MCMC sampling scheme involved in operationalizing the Bayesian estimation. The DA device enables to provide the estimation of all these quantities in a statistically efficient one-step procedure. The uncertainty stemming from partially missing covariate data is directly incorporated into parameter estimation. At every iteration of the algorithm an imputed version of the covariate data is used to sample from the set of full conditional posterior distributions. Vice versa, the iteratively updated parameter values resulting from posterior sampling can in turn be considered within the full conditional distribution of missing values. Thus, compared to existing methods the novel method carries out parameter estimation while handling missing values in background variables simultaneously. Taken together, there are several advantages resulting from such an approach. First, it is statistically efficient in the sense that values for the latent trait, item characteristics, and missing values of background variables are all provided at once, second, all possible sources of uncertainty are taken into account, and third, the approach is especially well suited to deal with latent variables corresponding to competencies or arising from hierarchical structures, where the mutual dependence can be directly handled in terms of the full conditional distributions inserted into the sampler.

The advantages show off in terms of statistical efficiency and the computational burden is possibly eased, when latent quantities in the sense of sufficient statistics can be used to specify the full conditional distributions of missing values. An empirical example using the NEPS further demonstrates the broad applicability of the approach to a wide range of social science topics. Besides permitting the estimation of competency scores and their correlations with the context variables purified from measurement error, any number of completed data sets arising from the MCMC output may also serve as multiple imputations of the missing background information. Future research may investigate in detail the possibilities to perform nested and non-nested model comparison via Bayes factors based on the marginal data likelihood. Also alternative models for the full conditional distributions of missing values or automated variable selection based on the spike-and-slab prior specification, see Ročková and George (2018), to determine which variables have group specific influence and which variables have homogeneous influence across the different groups, could be considered.

### Supplementary Information

Below is the link to the electronic supplementary material.

Supplementary file 1 (pdf 2994 KB)

## Footnotes

1 Thereby DA facilitates efficient sampling from the posterior distribution via augmenting the posterior distribution with quantities not necessarily being of primary interest, but possibly functioning as sufficient statistics and thus enabling and operationalizing Rao-Blackwellization. As a byproduct DA enables smoother sampling from the posterior distribution of the quantities of primary interest as either closed form sampling becomes available or the construction of an importance or enveloping density is considerably simplified, see Carlin and Louis (1998).

2 Note that also complete cases analysis, which excludes all observations having a missing value on any covariate from estimation, beside the inefficient use of the sample information in situations with high rates of missing values may result in biased estimation, especially when observations are missing at random (Little & Rubin, 2002, p. 41–44). Only in missing completely at random situations possibly related to multiple matrix designs estimation may stay unbiased.

3 When performing Maximum Likelihood based estimation typically implemented in terms of an Expectation Maximization algorithm, only point estimates are directly available but extra calculations are required to obtain corresponding uncertainty measures.

4 This may include information in terms of prevailing missing patterns, where Muthén et al. (1987) consider conditioning on missing data patterns for improved estimation.

5 The considered model framework incorporates an enriched multilevel structure compared to Fox and Glas (2001), whereas Aßmann et al. (2015) consider the case of Bayesian estimation for the homogeneous two-parameter normal ogive LRM for binary outcomes only.

6 In addition, the computational cost of the approach discussed by Neal and Kypraios (2015) grows exponentially with the total number of observations, whereas our MCMC based approach is linearly related to the number of observations.

7 The sequential imputations of Kong et al. (1994) resembles via use of predictive distributions an importance sampling approach, while our approach based on full conditional distributions resembles an efficient importance sampling approach as discussed by Richard and Zhang (2007).

8 The problem has been discussed extensively under the term *incidental parameter problem* in the statistics literature, see Lancaster (2000) for a survey.

9 Note that extensions in the form of random coefficients within groups or homogeneous coefficients across groups rendering the latent regression function as $\theta _i=\omega _{S_i}+X_i\gamma _{iL_i} +W_i\lambda +\epsilon _i$ with $\gamma _{iL_i}$ following a multivariate normal distribution with expectation $\mu _{\gamma _{L_i}}$ and covariance $\Omega _{L_i}$ and $W_i$ denoting a set of covariates with homogeneous influence are also possible as discussed in Aßmann et al. (2011).

10 This extends also towards missing-by-design values in item responses. Sampling of missing-by-design values $Y_{\text {mis}}$ are implied by Eq. (1) in the paper, where $y_{ij}^*$ follows then a normal distribution not subject to truncation as the truncation is implied by the observed values in *Y* only. The completed $Y^*$ and hence the completed *Y* might possibly be helpful for sampling values in $X_{\text {mis}}$, as pointed out by von Hippel (2007). However, as illustrated and implied by the considered model framework in the paper, $\theta$ serves takes a role of a sufficient statistic also for $Y^*$, and thus consideration of sampled $Y_{\text {mis}}$ values within the imputation of $X_{\text {mis}}$ does not necessarily result in further gains in terms of statistical efficiency. Given this, we point out that the suggested approach should be applied to data situations, where at least some elements of $y_i$ are observed for each individual $i=1\ldots ,N$. Situations, where several competence domains are investigated can be addressed via multivariate extensions of the suggested modeling framework.

11 Consideration of sufficient statistics may also serve as a guiding principle for model specification.

12 Note that sampling from $f(X_{\text {mis}}|\theta ,Y^*,\omega ,\psi ,Y,X_{\text {obs}},S,L)$ will be based on sequential iterative sampling from the set of univariate full conditional distributions for each variable $X_{\text {mis}}^{(p)}$, $p=1,\ldots ,P$, see also Sect. 2.2 for details.

13 In combination with sampling from the empirical cumulative distribution function, i.e. sampling from the range of observed values only, this ensures that the CART-BB approach towards full conditional distributions does involve only proper prior distributions thus ensuring the existence of the integrating constant of the joint posterior distribution. Furthermore, the existence of the joint posterior distribution and the corresponding integrating constant as implied by the Eq. (8) is directly ensured in case the missing values relate to variables with finite sample spaces. In case the missing values relate to variables with theoretically possible countable infinite or uncountable infinite sample spaces, the CART-BB algorithm constructs the empirical cumulative distribution function implied by the obtained partition based on measures of homogeneity, e.g. the variance, and incorporates the restriction to the range of observed values as a modeling assumption. Thus, the suggested approach may be most useful in situations with many categorical variables, as in our empirical applications. For applications where the restriction to the range of observed values raises concerns, the suggested CART-BB approach could be applied to the set of categorical values only and alternative modeling approaches for the missing values for variables within continuous infinite support may be considered as well.

14 The proposed Bayesian analysis and its MCMC implementation is further suited to incorporate information arising from weighting factors. In case of non-stochastic weights, e.g. design weights, the variables entering the modeling can be transformed accordingly, whereas in case of stochastic weights, e.g. non-response adjusted weights typically handled via replication weights, the variables can be transformed within each MCMC iteration.

15 In case that also interaction terms are considered, $(X_{\text{ com }}^{({\setminus } p)})$ also subsumes all columns referring to cross terms not involving variable *p*. Cross terms involving variable *p* are hence not subject to modeling but updated each time an underlying variable has been updated.

16 Sampling from the empirical distribution function via the Bayesian bootstrap corresponds to running the data generating process of a parametric imputation model, with involved parameters being sampled from the estimated distributions in order to fully account for the uncertainty of the data generating process, i.e. the uncertainty how the empirical cumulative distribution function would look like if the missing values would be observed.

17 The IBM benchmark is hence in line with a typical multiple imputation strategy, although no combining rules are required as sampling is performed within the MCMC sampler. This ensures further that the comparison of the different approaches is conditional on the same level of numerical precision as implied by the number of MCMC iterations after burn-in.

18 For ten items we have missing rates of less than $2\%$, less than $5\%$ for another eight items, for three items we have the range from $5\% - 10\%$ and only one item has a missing rate of $20\%$.

19 Regarding socio-economic status, there are many operationalizations implemented in the NEPS. In line with recent analyses of the PISA data (OECD, 2013a, p. 132), we took the highest occupational level of parents measured by the index ISEI-08 (Ganzeboom, 2010) and calculated a variable *HISEI* as the higher ISEI-08 score of either the students’ mother or the students’ father or the only available score. To calibrate the scale of the regression coefficient associated with *HISEI*, the original values are divided by 100. *HISEI* ranges from 1.16 to 8.90 with higher values indicating a higher level of occupational status. This variable in particular shows strong differences between the school types which can be seen in Table 8. Age at assessment and school exposure are defined as the difference between date of assessment and date of birth or date of school entry respectively.

20 Results on item characteristics (discrimination, difficulty, and cut-off parameters) are available within the supplementary material.
