# Supplementary material for: A Bayesian Approach Towards Missing Covariate Data in Multilevel Latent Regression Models
Source: Psychometrika. 2022 Nov 23;88(4):1495–528. doi: 10.1007/s11336-022-09888-0 (PMC10656345; doi:10.1007/s11336-022-09888-0)

SUPPLEMENTARY MATERIAL

ESTIMATION OF ITEM PARAMETERS WITHIN SIMULATION

Table 1: Simulation study (Scenario 1 with $X_1 = 19\%$, $X_2 = 26\%$, and overall = 33% missings) - True parameter values, mean posterior medians and standard deviations of item characteristics over 1000 replications obtained from BD, CC, IBM and DART

		Average					Averaged standard deviation				
	True	BD	CC	IBM	DART	DART-m	BD	CC	IBM	DART	DART-m
Item discrimination											
α_1	1.017	1.016	1.017	1.016	1.016	1.016	0.031	0.046	0.031	0.031	0.031
α_2	0.964	0.966	0.966	0.966	0.966	0.966	0.029	0.045	0.029	0.029	0.029
α_3	1.326	1.326	1.318	1.326	1.326	1.326	0.042	0.061	0.042	0.042	0.042
α_4	1.080	1.081	1.075	1.081	1.081	1.081	0.034	0.052	0.034	0.034	0.034
α_5	0.867	0.867	0.866	0.867	0.867	0.867	0.027	0.043	0.027	0.027	0.027
α_6	0.979	0.980	0.975	0.980	0.980	0.980	0.031	0.050	0.031	0.031	0.031
α_7	0.775	0.775	0.774	0.775	0.775	0.775	0.025	0.040	0.025	0.025	0.025
α_8	1.095	1.097	1.107	1.097	1.097	1.097	0.036	0.046	0.036	0.036	0.036
α_9	0.850	0.849	0.849	0.849	0.849	0.849	0.026	0.041	0.026	0.026	0.026
α_{10}	1.164	1.164	1.162	1.164	1.164	1.164	0.036	0.052	0.036	0.036	0.036
α_{11}	1.111	1.113	1.107	1.113	1.113	1.113	0.034	0.052	0.034	0.034	0.034
α_{12}	0.784	0.784	0.782	0.784	0.784	0.784	0.026	0.045	0.026	0.026	0.026
α_{13}	1.107	1.108	1.113	1.108	1.108	1.108	0.034	0.046	0.034	0.034	0.034
α_{14}	1.412	1.414	1.424	1.413	1.413	1.413	0.047	0.059	0.047	0.047	0.047
α_{15}	0.917	0.918	0.920	0.918	0.918	0.918	0.028	0.042	0.028	0.028	0.028
α_{16}	0.779	0.781	0.781	0.781	0.781	0.781	0.025	0.041	0.025	0.025	0.025
α_{17}	0.841	0.841	0.850	0.841	0.841	0.841	0.029	0.039	0.029	0.029	0.029
α_{18}	1.119	1.120	1.125	1.119	1.120	1.120	0.034	0.048	0.034	0.034	0.034
α_{19}	0.865	0.865	0.872	0.865	0.865	0.865	0.023	0.033	0.023	0.023	0.023
α_{20}	1.261	1.262	1.265	1.262	1.262	1.262	0.030	0.042	0.030	0.030	0.030
Item difficulty											
β_1	−0.070	−0.071	−0.071	−0.071	−0.071	−0.071	0.025	0.037	0.025	0.025	0.025
β_2	−0.082	−0.084	−0.085	−0.084	−0.084	−0.084	0.025	0.036	0.025	0.025	0.025
β_3	−0.196	−0.198	−0.198	−0.198	−0.198	−0.198	0.028	0.038	0.028	0.028	0.028
β_4	−0.375	−0.376	−0.378	−0.376	−0.376	−0.376	0.026	0.037	0.026	0.026	0.026
β_5	−0.237	−0.237	−0.239	−0.237	−0.237	−0.237	0.024	0.036	0.024	0.024	0.024
β_6	−0.466	−0.467	−0.470	−0.467	−0.467	−0.467	0.026	0.036	0.026	0.026	0.026
β_7	−0.327	−0.328	−0.331	−0.328	−0.328	−0.328	0.024	0.035	0.024	0.024	0.024
β_8	0.867	0.869	0.875	0.869	0.869	0.869	0.034	0.046	0.034	0.034	0.034
β_9	−0.166	−0.167	−0.170	−0.167	−0.167	−0.167	0.024	0.036	0.024	0.024	0.024
β_{10}	0.008	0.006	0.004	0.006	0.006	0.006	0.026	0.038	0.026	0.026	0.026
β_{11}	−0.252	−0.251	−0.255	−0.251	−0.251	−0.251	0.026	0.037	0.026	0.026	0.026
β_{12}	−0.644	−0.645	−0.648	−0.645	−0.645	−0.645	0.025	0.036	0.025	0.025	0.025
β_{13}	0.522	0.522	0.524	0.522	0.522	0.522	0.029	0.042	0.029	0.029	0.029
β_{14}	0.858	0.858	0.866	0.857	0.857	0.857	0.037	0.050	0.037	0.037	0.037
β_{15}	0.032	0.031	0.031	0.031	0.031	0.031	0.025	0.037	0.025	0.025	0.025
β_{16}	−0.340	−0.340	−0.343	−0.340	−0.340	−0.340	0.024	0.035	0.024	0.024	0.024
β_{17}	0.887	0.887	0.892	0.887	0.887	0.887	0.031	0.044	0.031	0.031	0.031
β_{18}	0.301	0.301	0.304	0.301	0.301	0.301	0.027	0.040	0.027	0.027	0.027
β_{19}	0.101	0.101	0.103	0.101	0.101	0.101	0.024	0.035	0.024	0.024	0.024
β_{20}	−0.412	−0.412	−0.413	−0.412	−0.412	−0.412	0.027	0.037	0.027	0.027	0.027
Item category cutoff											
$\kappa_{19,1}$	0.500	0.500	0.501	0.500	0.500	0.500	0.020	0.024	0.020	0.020	0.020
$\kappa_{19,2}$	1.000	0.999	1.000	0.999	0.999	0.999	0.028	0.031	0.028	0.028	0.028
$\kappa_{20,1}$	0.700	0.699	0.702	0.699	0.699	0.699	0.028	0.034	0.028	0.028	0.028
$\kappa_{20,2}$	1.400	1.399	1.401	1.399	1.399	1.399	0.037	0.044	0.037	0.037	0.037

Notes: $G = 2$; $C = 20$; $N = 4000$; $J = 20$; $n_{iter} = 20000 + 5000$. BD = before deletion; CC = complete cases; IBM = multiple imputation before modeling based on observed data; DART = data augmentation using sequential recursive partitioning based on all data and latent parameters. DART-m = data augmentation using sequential recursive partitioning based on the sufficient statistics θ and ω.

Table 2: Simulation study (Scenario 1 with X_1 = 19%, X_2 = 26%, and overall = 33% missings) - RMSEs and coverage ratios of item characteristics over 1000 replications obtained from BD, CC, IBM and DART

	RMSE					Coverage				
	BD	CC	IBM	DART	DART-m	BD	CC	IBM	DART	DART-m
Item discrimination										
α_1	0.031	0.047	0.031	0.031	0.031	0.949	0.947	0.951	0.950	0.951
α_2	0.030	0.047	0.030	0.030	0.030	0.938	0.940	0.938	0.942	0.936
α_3	0.042	0.060	0.042	0.042	0.042	0.946	0.956	0.954	0.946	0.951
α_4	0.034	0.052	0.034	0.034	0.034	0.948	0.950	0.946	0.947	0.946
α_5	0.028	0.043	0.028	0.028	0.028	0.945	0.942	0.939	0.945	0.944
α_6	0.030	0.049	0.030	0.030	0.030	0.956	0.952	0.955	0.952	0.954
α_7	0.026	0.041	0.026	0.026	0.026	0.937	0.953	0.938	0.937	0.936
α_8	0.035	0.047	0.035	0.035	0.035	0.948	0.945	0.952	0.952	0.953
α_9	0.026	0.041	0.026	0.026	0.026	0.955	0.944	0.952	0.954	0.954
α_{10}	0.035	0.051	0.035	0.035	0.035	0.958	0.952	0.956	0.959	0.960
α_{11}	0.035	0.052	0.035	0.035	0.035	0.941	0.957	0.940	0.942	0.944
α_{12}	0.027	0.045	0.027	0.027	0.027	0.940	0.955	0.940	0.939	0.941
α_{13}	0.034	0.044	0.034	0.034	0.034	0.954	0.958	0.951	0.955	0.954
α_{14}	0.047	0.059	0.047	0.047	0.047	0.942	0.945	0.942	0.942	0.945
α_{15}	0.028	0.043	0.028	0.028	0.028	0.946	0.947	0.947	0.948	0.947
α_{16}	0.025	0.040	0.025	0.025	0.025	0.956	0.955	0.955	0.958	0.957
α_{17}	0.028	0.040	0.028	0.028	0.028	0.953	0.943	0.954	0.957	0.955
α_{18}	0.035	0.049	0.035	0.035	0.035	0.944	0.950	0.939	0.944	0.943
α_{19}	0.023	0.033	0.023	0.023	0.023	0.953	0.941	0.953	0.953	0.953
α_{20}	0.029	0.041	0.029	0.029	0.029	0.959	0.961	0.961	0.960	0.959
Item difficulty										
β_1	0.025	0.036	0.025	0.025	0.025	0.957	0.959	0.955	0.957	0.955
β_2	0.024	0.037	0.024	0.024	0.024	0.951	0.943	0.956	0.951	0.954
β_3	0.029	0.039	0.029	0.029	0.029	0.938	0.948	0.938	0.939	0.939
β_4	0.025	0.037	0.025	0.025	0.025	0.957	0.946	0.958	0.959	0.959
β_5	0.023	0.035	0.023	0.023	0.023	0.961	0.959	0.960	0.960	0.958
β_6	0.025	0.037	0.026	0.025	0.025	0.962	0.946	0.963	0.965	0.963
β_7	0.024	0.035	0.024	0.024	0.024	0.952	0.938	0.954	0.949	0.950
β_8	0.033	0.046	0.033	0.033	0.033	0.956	0.947	0.951	0.955	0.957
β_9	0.024	0.036	0.024	0.024	0.024	0.952	0.953	0.948	0.948	0.947
β_{10}	0.026	0.038	0.026	0.026	0.026	0.959	0.950	0.959	0.960	0.961
β_{11}	0.026	0.038	0.026	0.026	0.026	0.956	0.946	0.950	0.953	0.955
β_{12}	0.025	0.037	0.026	0.025	0.026	0.957	0.949	0.952	0.957	0.956
β_{13}	0.029	0.040	0.029	0.029	0.029	0.951	0.956	0.954	0.953	0.951
β_{14}	0.036	0.049	0.036	0.036	0.036	0.959	0.955	0.957	0.957	0.957
β_{15}	0.024	0.037	0.024	0.024	0.024	0.951	0.943	0.948	0.949	0.950
β_{16}	0.025	0.036	0.025	0.025	0.025	0.932	0.943	0.930	0.934	0.932
β_{17}	0.030	0.043	0.030	0.030	0.030	0.957	0.964	0.958	0.959	0.960
β_{18}	0.028	0.040	0.028	0.028	0.028	0.954	0.946	0.946	0.949	0.950
β_{19}	0.023	0.035	0.023	0.023	0.023	0.962	0.954	0.962	0.962	0.962
β_{20}	0.026	0.037	0.026	0.026	0.026	0.960	0.954	0.959	0.959	0.957
Item category cutoff										
$\kappa_{19,1}$	0.020	0.023	0.020	0.020	0.020	0.957	0.953	0.958	0.957	0.957
$\kappa_{19,2}$	0.029	0.032	0.029	0.029	0.029	0.944	0.939	0.946	0.948	0.948
$\kappa_{20,1}$	0.028	0.034	0.028	0.028	0.028	0.950	0.951	0.948	0.948	0.949
$\kappa_{20,2}$	0.036	0.043	0.036	0.036	0.036	0.952	0.956	0.952	0.954	0.952

Notes: $G = 2$; $C = 20$; $N = 4000$; $J = 20$; $n_{iter} = 20000 + 5000$. RMSE = root mean square error; BD = before deletion; CC = complete cases; IBM = multiple imputation before modeling based on observed data; DART = data augmentation using sequential recursive partitioning based on all data and latent parameters. DART-m = data augmentation using sequential recursive partitioning based on the sufficient statistics θ and ω.

Table 3: Simulation study (Scenario 2 with $X_1 = 40\%$, $X_2 = 50\%$, and overall = 59% missings) - True parameter values, mean posterior medians and standard deviations of item characteristics over 1000 replications obtained from BD, CC, IBM and DART

		Average					Averaged standard deviation				
	True	BD	CC	IBM	DART	DART-m	BD	CC	IBM	DART	DART-m
Item discrimination											
α_1	1.017	1.017	1.019	1.017	1.017	1.017	0.031	0.076	0.031	0.031	0.031
α_2	0.964	0.966	0.975	0.966	0.966	0.966	0.029	0.074	0.029	0.029	0.029
α_3	1.326	1.326	1.315	1.325	1.325	1.325	0.042	0.105	0.042	0.042	0.042
α_4	1.080	1.081	1.073	1.081	1.081	1.081	0.034	0.090	0.034	0.034	0.034
α_5	0.867	0.867	0.870	0.867	0.868	0.867	0.027	0.071	0.027	0.027	0.027
α_6	0.979	0.980	0.979	0.980	0.980	0.980	0.031	0.086	0.031	0.031	0.031
α_7	0.775	0.775	0.780	0.775	0.776	0.776	0.025	0.068	0.025	0.025	0.025
α_8	1.095	1.097	1.109	1.097	1.097	1.097	0.036	0.068	0.036	0.036	0.036
α_9	0.850	0.849	0.854	0.849	0.849	0.849	0.026	0.068	0.026	0.026	0.026
α_{10}	1.164	1.164	1.163	1.164	1.164	1.164	0.036	0.085	0.036	0.036	0.036
α_{11}	1.111	1.113	1.111	1.113	1.113	1.113	0.034	0.089	0.034	0.034	0.034
α_{12}	0.784	0.784	0.787	0.784	0.785	0.785	0.026	0.078	0.026	0.026	0.026
α_{13}	1.107	1.109	1.113	1.109	1.108	1.108	0.034	0.072	0.034	0.034	0.034
α_{14}	1.412	1.414	1.421	1.413	1.412	1.412	0.047	0.088	0.047	0.047	0.047
α_{15}	0.917	0.918	0.923	0.918	0.918	0.918	0.028	0.068	0.028	0.028	0.028
α_{16}	0.779	0.781	0.787	0.781	0.781	0.781	0.025	0.068	0.025	0.025	0.025
α_{17}	0.841	0.840	0.851	0.840	0.840	0.840	0.029	0.057	0.029	0.029	0.029
α_{18}	1.119	1.120	1.125	1.119	1.119	1.119	0.034	0.076	0.034	0.034	0.034
α_{19}	0.865	0.865	0.876	0.865	0.865	0.865	0.023	0.051	0.023	0.023	0.023
α_{20}	1.261	1.262	1.266	1.262	1.262	1.262	0.030	0.067	0.030	0.030	0.030
Item difficulty											
β_1	−0.070	−0.071	−0.073	−0.071	−0.071	−0.071	0.025	0.074	0.025	0.025	0.025
β_2	−0.082	−0.084	−0.080	−0.084	−0.084	−0.084	0.025	0.073	0.025	0.025	0.025
β_3	−0.196	−0.198	−0.210	−0.198	−0.198	−0.198	0.028	0.079	0.028	0.028	0.028
β_4	−0.375	−0.376	−0.386	−0.376	−0.376	−0.376	0.026	0.077	0.026	0.026	0.026
β_5	−0.237	−0.237	−0.242	−0.237	−0.237	−0.237	0.024	0.073	0.024	0.024	0.024
β_6	−0.466	−0.466	−0.470	−0.466	−0.466	−0.466	0.026	0.076	0.026	0.026	0.026
β_7	−0.327	−0.328	−0.328	−0.328	−0.328	−0.328	0.024	0.072	0.024	0.024	0.024
β_8	0.867	0.869	0.877	0.869	0.869	0.869	0.034	0.080	0.034	0.034	0.034
β_9	−0.166	−0.167	−0.167	−0.167	−0.167	−0.167	0.024	0.072	0.024	0.024	0.024
β_{10}	0.008	0.006	0.003	0.006	0.006	0.006	0.026	0.076	0.026	0.026	0.026
β_{11}	−0.252	−0.251	−0.255	−0.251	−0.251	−0.251	0.026	0.076	0.026	0.026	0.026
β_{12}	−0.644	−0.645	−0.648	−0.645	−0.644	−0.645	0.025	0.077	0.025	0.025	0.025
β_{13}	0.522	0.522	0.523	0.522	0.522	0.522	0.029	0.077	0.029	0.029	0.029
β_{14}	0.858	0.858	0.864	0.857	0.857	0.857	0.037	0.088	0.037	0.037	0.037
β_{15}	0.032	0.031	0.032	0.031	0.031	0.031	0.025	0.072	0.025	0.025	0.025
β_{16}	−0.340	−0.340	−0.340	−0.340	−0.340	−0.340	0.024	0.072	0.024	0.024	0.024
β_{17}	0.887	0.887	0.893	0.887	0.887	0.887	0.031	0.075	0.031	0.031	0.031
β_{18}	0.301	0.301	0.304	0.301	0.301	0.301	0.027	0.076	0.027	0.027	0.027
β_{19}	0.101	0.101	0.108	0.101	0.101	0.101	0.024	0.062	0.024	0.024	0.024
β_{20}	−0.412	−0.412	−0.412	−0.412	−0.412	−0.412	0.027	0.069	0.027	0.027	0.027
Item category cutoff											
$\kappa_{19,1}$	0.500	0.500	0.500	0.500	0.500	0.500	0.020	0.032	0.020	0.020	0.020
$\kappa_{19,2}$	1.000	0.999	1.000	1.000	1.000	0.999	0.028	0.041	0.028	0.028	0.028
$\kappa_{20,1}$	0.700	0.699	0.700	0.699	0.699	0.699	0.028	0.052	0.028	0.028	0.028
$\kappa_{20,2}$	1.400	1.399	1.400	1.399	1.399	1.399	0.037	0.063	0.037	0.037	0.037

Notes: $G = 2$; $C = 20$; $N = 4000$; $J = 20$; $n_{iter} = 20000 + 5000$. BD = before deletion; CC = complete cases; IBM = multiple imputation before modeling based on observed data; DART = data augmentation using sequential recursive partitioning based on all data and latent parameters. DART-m = data augmentation using sequential recursive partitioning based on the sufficient statistics θ and ω.

Table 4: Simulation study (Scenario 2 with X_1 = 40%, X_2 = 50%, and overall = 59% missings) - RMSEs and coverage ratios of item characteristics over 1000 replications obtained from BD, CC, IBM and DART

	RMSE					Coverage				
	BD	CC	IBM	DART	DART-m	BD	CC	IBM	DART	DART-m
Item discrimination										
α_1	0.030	0.077	0.030	0.030	0.030	0.951	0.942	0.949	0.955	0.955
α_2	0.030	0.075	0.030	0.030	0.030	0.938	0.949	0.936	0.935	0.939
α_3	0.042	0.102	0.042	0.042	0.042	0.946	0.964	0.950	0.953	0.950
α_4	0.034	0.090	0.034	0.034	0.034	0.946	0.941	0.942	0.945	0.944
α_5	0.028	0.073	0.028	0.028	0.028	0.946	0.945	0.948	0.944	0.945
α_6	0.030	0.086	0.030	0.030	0.030	0.956	0.946	0.951	0.955	0.957
α_7	0.026	0.066	0.026	0.026	0.026	0.936	0.950	0.940	0.936	0.940
α_8	0.035	0.070	0.035	0.035	0.035	0.950	0.951	0.956	0.955	0.954
α_9	0.026	0.068	0.026	0.026	0.026	0.956	0.954	0.958	0.955	0.953
α_{10}	0.035	0.083	0.035	0.035	0.035	0.956	0.952	0.957	0.956	0.958
α_{11}	0.035	0.089	0.035	0.035	0.035	0.940	0.953	0.943	0.941	0.944
α_{12}	0.027	0.078	0.027	0.027	0.027	0.939	0.945	0.939	0.940	0.941
α_{13}	0.034	0.071	0.034	0.034	0.034	0.954	0.946	0.951	0.953	0.950
α_{14}	0.047	0.088	0.047	0.047	0.047	0.941	0.947	0.942	0.946	0.943
α_{15}	0.028	0.068	0.028	0.028	0.028	0.945	0.951	0.946	0.946	0.947
α_{16}	0.025	0.072	0.025	0.025	0.025	0.955	0.941	0.958	0.961	0.962
α_{17}	0.028	0.059	0.028	0.028	0.028	0.952	0.938	0.953	0.953	0.953
α_{18}	0.035	0.076	0.035	0.035	0.035	0.943	0.951	0.943	0.942	0.942
α_{19}	0.023	0.052	0.023	0.023	0.023	0.952	0.953	0.952	0.952	0.952
α_{20}	0.030	0.065	0.030	0.030	0.030	0.957	0.953	0.956	0.956	0.956
Item difficulty										
β_1	0.024	0.075	0.024	0.024	0.024	0.957	0.949	0.956	0.955	0.954
β_2	0.024	0.073	0.024	0.024	0.024	0.952	0.950	0.955	0.953	0.955
β_3	0.029	0.081	0.029	0.029	0.029	0.937	0.953	0.937	0.941	0.937
β_4	0.025	0.076	0.025	0.025	0.025	0.957	0.951	0.956	0.956	0.957
β_5	0.023	0.073	0.023	0.023	0.023	0.959	0.950	0.958	0.960	0.960
β_6	0.025	0.077	0.025	0.025	0.025	0.963	0.951	0.965	0.965	0.965
β_7	0.024	0.070	0.024	0.024	0.024	0.952	0.953	0.950	0.953	0.951
β_8	0.033	0.083	0.033	0.033	0.033	0.956	0.948	0.957	0.956	0.956
β_9	0.024	0.072	0.024	0.024	0.024	0.952	0.957	0.948	0.948	0.949
β_{10}	0.026	0.074	0.026	0.026	0.026	0.957	0.955	0.957	0.957	0.956
β_{11}	0.026	0.077	0.026	0.026	0.026	0.957	0.946	0.955	0.954	0.952
β_{12}	0.026	0.079	0.026	0.026	0.026	0.956	0.940	0.954	0.956	0.955
β_{13}	0.029	0.077	0.029	0.029	0.029	0.952	0.957	0.952	0.951	0.955
β_{14}	0.036	0.088	0.036	0.036	0.036	0.958	0.953	0.956	0.958	0.955
β_{15}	0.024	0.072	0.024	0.024	0.024	0.951	0.958	0.950	0.949	0.949
β_{16}	0.025	0.077	0.025	0.025	0.025	0.930	0.938	0.931	0.928	0.929
β_{17}	0.030	0.076	0.030	0.030	0.030	0.957	0.948	0.959	0.959	0.959
β_{18}	0.028	0.076	0.028	0.028	0.028	0.952	0.947	0.950	0.950	0.950
β_{19}	0.023	0.062	0.023	0.023	0.023	0.962	0.952	0.961	0.962	0.961
β_{20}	0.026	0.067	0.026	0.026	0.026	0.959	0.957	0.959	0.958	0.957
Item category cutoff										
$\kappa_{19,1}$	0.020	0.032	0.020	0.020	0.020	0.955	0.953	0.956	0.956	0.957
$\kappa_{19,2}$	0.029	0.042	0.029	0.029	0.029	0.944	0.945	0.947	0.946	0.945
$\kappa_{20,1}$	0.028	0.051	0.028	0.028	0.028	0.949	0.950	0.946	0.949	0.947
$\kappa_{20,2}$	0.037	0.064	0.037	0.037	0.037	0.950	0.945	0.949	0.949	0.951

Notes: $G = 2$; $C = 20$; $N = 4000$; $J = 20$; $n_{iter} = 20000 + 5000$. RMSE = root mean square error; BD = before deletion; CC = complete cases; IBM = multiple imputation before modeling based on observed data; DART = data augmentation using sequential recursive partitioning based on all data and latent parameters. DART-m = data augmentation using sequential recursive partitioning based on the sufficient statistics θ and ω.

Table 5: Simulation study (Scenario 3 with $X_1 = 20\%$, $X_2 = 36\%$, and overall = 46% missings) - True parameter values, mean posterior medians and standard deviations of item characteristics over 1000 replications obtained from BD, CC, IBM and DART

		Average					Averaged standard deviation				
	True	BD	CC	IBM	DART	DART-m	BD	CC	IBM	DART	DART-m
Item discrimination											
α_1	1.017	1.017	1.015	1.017	1.017	1.017	0.031	0.042	0.031	0.031	0.031
α_2	0.964	0.966	0.965	0.966	0.966	0.966	0.029	0.040	0.029	0.029	0.029
α_3	1.326	1.326	1.327	1.325	1.326	1.326	0.042	0.057	0.042	0.042	0.042
α_4	1.080	1.081	1.083	1.081	1.081	1.081	0.034	0.045	0.034	0.034	0.034
α_5	0.867	0.867	0.868	0.867	0.867	0.867	0.027	0.037	0.027	0.027	0.027
α_6	0.979	0.980	0.982	0.980	0.980	0.980	0.031	0.041	0.031	0.031	0.031
α_7	0.775	0.775	0.777	0.775	0.775	0.775	0.025	0.034	0.025	0.025	0.025
α_8	1.095	1.097	1.099	1.097	1.097	1.097	0.036	0.052	0.036	0.036	0.036
α_9	0.850	0.849	0.850	0.849	0.849	0.849	0.026	0.036	0.026	0.026	0.026
α_{10}	1.164	1.164	1.166	1.164	1.164	1.164	0.036	0.049	0.036	0.036	0.036
α_{11}	1.111	1.113	1.113	1.113	1.113	1.113	0.034	0.047	0.034	0.034	0.034
α_{12}	0.784	0.784	0.785	0.784	0.784	0.784	0.026	0.035	0.026	0.026	0.026
α_{13}	1.107	1.109	1.109	1.109	1.109	1.109	0.034	0.049	0.034	0.034	0.034
α_{14}	1.412	1.414	1.412	1.414	1.413	1.413	0.047	0.068	0.047	0.047	0.047
α_{15}	0.917	0.918	0.919	0.918	0.918	0.918	0.028	0.039	0.028	0.028	0.028
α_{16}	0.779	0.781	0.781	0.781	0.781	0.781	0.025	0.034	0.025	0.025	0.025
α_{17}	0.841	0.840	0.841	0.841	0.840	0.840	0.029	0.042	0.029	0.029	0.029
α_{18}	1.119	1.120	1.118	1.119	1.120	1.120	0.034	0.048	0.034	0.034	0.034
α_{19}	0.865	0.865	0.865	0.865	0.865	0.865	0.023	0.033	0.023	0.023	0.023
α_{20}	1.261	1.262	1.262	1.262	1.262	1.262	0.030	0.042	0.030	0.030	0.030
Item difficulty											
β_1	−0.070	−0.071	−0.069	−0.071	−0.071	−0.071	0.025	0.033	0.025	0.025	0.025
β_2	−0.082	−0.084	−0.084	−0.084	−0.084	−0.084	0.025	0.033	0.025	0.025	0.025
β_3	−0.196	−0.198	−0.199	−0.198	−0.198	−0.198	0.028	0.037	0.028	0.028	0.028
β_4	−0.375	−0.376	−0.377	−0.376	−0.376	−0.376	0.026	0.036	0.026	0.026	0.026
β_5	−0.237	−0.237	−0.238	−0.237	−0.237	−0.237	0.024	0.033	0.024	0.024	0.024
β_6	−0.466	−0.466	−0.468	−0.466	−0.466	−0.466	0.026	0.036	0.026	0.026	0.026
β_7	−0.327	−0.328	−0.329	−0.328	−0.328	−0.328	0.024	0.032	0.024	0.024	0.024
β_8	0.867	0.869	0.871	0.869	0.869	0.869	0.034	0.043	0.034	0.034	0.034
β_9	−0.166	−0.167	−0.167	−0.167	−0.167	−0.167	0.024	0.032	0.024	0.024	0.024
β_{10}	0.008	0.006	0.005	0.006	0.006	0.006	0.026	0.035	0.026	0.026	0.026
β_{11}	−0.252	−0.251	−0.250	−0.251	−0.251	−0.251	0.026	0.035	0.026	0.026	0.026
β_{12}	−0.644	−0.645	−0.645	−0.645	−0.645	−0.645	0.025	0.036	0.025	0.025	0.025
β_{13}	0.522	0.522	0.522	0.522	0.522	0.522	0.029	0.037	0.029	0.029	0.029
β_{14}	0.858	0.858	0.858	0.858	0.857	0.857	0.037	0.047	0.037	0.037	0.037
β_{15}	0.032	0.031	0.032	0.031	0.031	0.031	0.025	0.032	0.025	0.025	0.025
β_{16}	−0.340	−0.340	−0.340	−0.340	−0.340	−0.340	0.024	0.033	0.024	0.024	0.024
β_{17}	0.887	0.887	0.889	0.887	0.887	0.887	0.031	0.039	0.031	0.031	0.031
β_{18}	0.301	0.301	0.301	0.301	0.301	0.301	0.027	0.035	0.027	0.027	0.027
β_{19}	0.101	0.101	0.101	0.101	0.101	0.101	0.024	0.032	0.024	0.024	0.024
β_{20}	−0.412	−0.412	−0.412	−0.412	−0.412	−0.412	0.027	0.036	0.027	0.027	0.027
Item category cutoff											
$\kappa_{19,1}$	0.500	0.500	0.501	0.500	0.500	0.500	0.020	0.029	0.020	0.020	0.020
$\kappa_{19,2}$	1.000	0.999	1.000	1.000	0.999	0.999	0.028	0.040	0.028	0.028	0.028
$\kappa_{20,1}$	0.700	0.699	0.697	0.699	0.699	0.699	0.028	0.037	0.028	0.028	0.028
$\kappa_{20,2}$	1.400	1.399	1.397	1.399	1.399	1.399	0.037	0.051	0.037	0.037	0.037

Notes: $G = 2$; $C = 20$; $N = 4000$; $J = 20$; $n_{iter} = 20000 + 5000$. BD = before deletion; CC = complete cases; IBM = multiple imputation before modeling based on observed data; DART = data augmentation using sequential recursive partitioning based on all data and latent parameters. DART-m = data augmentation using sequential recursive partitioning based on the sufficient statistics θ and ω.

Table 6: Simulation study (Scenario 3 with $X_1 = 20\%$, $X_2 = 36\%$, and overall $= 46\%$ missings) - RMSEs and coverage ratios of item characteristics over 1000 replications obtained from BD, CC, IBM and DART

	RMSE					Coverage				
	BD	CC	IBM	DART	DART-m	BD	CC	IBM	DART	DART-m
Item discrimination										
α_1	0.030	0.043	0.030	0.030	0.030	0.953	0.956	0.954	0.952	0.951
α_2	0.030	0.040	0.030	0.030	0.030	0.939	0.954	0.942	0.942	0.942
α_3	0.042	0.058	0.042	0.042	0.042	0.947	0.951	0.954	0.951	0.949
α_4	0.034	0.046	0.034	0.034	0.034	0.946	0.944	0.947	0.945	0.948
α_5	0.028	0.038	0.028	0.028	0.028	0.945	0.944	0.946	0.945	0.945
α_6	0.030	0.041	0.030	0.030	0.030	0.960	0.954	0.956	0.957	0.957
α_7	0.026	0.034	0.026	0.026	0.026	0.936	0.944	0.936	0.935	0.938
α_8	0.035	0.052	0.035	0.035	0.035	0.952	0.939	0.953	0.951	0.953
α_9	0.026	0.036	0.026	0.026	0.026	0.957	0.957	0.956	0.953	0.954
α_{10}	0.035	0.048	0.035	0.035	0.035	0.957	0.953	0.957	0.959	0.956
α_{11}	0.035	0.048	0.035	0.035	0.035	0.943	0.944	0.943	0.941	0.942
α_{12}	0.027	0.036	0.027	0.027	0.027	0.938	0.940	0.937	0.940	0.940
α_{13}	0.034	0.049	0.034	0.034	0.034	0.952	0.951	0.953	0.951	0.948
α_{14}	0.047	0.066	0.047	0.047	0.047	0.946	0.956	0.944	0.942	0.940
α_{15}	0.028	0.039	0.028	0.028	0.028	0.947	0.949	0.942	0.942	0.945
α_{16}	0.025	0.034	0.025	0.025	0.025	0.957	0.957	0.952	0.957	0.956
α_{17}	0.028	0.042	0.029	0.028	0.029	0.951	0.947	0.956	0.954	0.957
α_{18}	0.035	0.048	0.035	0.035	0.036	0.942	0.955	0.938	0.938	0.938
α_{19}	0.023	0.032	0.023	0.023	0.023	0.950	0.960	0.953	0.952	0.952
α_{20}	0.030	0.043	0.030	0.030	0.030	0.958	0.941	0.954	0.955	0.958
Item difficulty										
β_1	0.024	0.033	0.024	0.024	0.024	0.955	0.947	0.954	0.955	0.957
β_2	0.024	0.033	0.024	0.024	0.024	0.954	0.958	0.954	0.953	0.955
β_3	0.029	0.038	0.029	0.029	0.029	0.938	0.947	0.938	0.938	0.939
β_4	0.025	0.036	0.025	0.025	0.025	0.955	0.949	0.958	0.958	0.959
β_5	0.023	0.032	0.023	0.023	0.023	0.957	0.944	0.955	0.958	0.958
β_6	0.025	0.035	0.025	0.025	0.025	0.964	0.942	0.965	0.965	0.963
β_7	0.024	0.032	0.024	0.024	0.024	0.952	0.947	0.953	0.954	0.952
β_8	0.033	0.043	0.033	0.033	0.033	0.954	0.955	0.954	0.952	0.955
β_9	0.024	0.031	0.024	0.024	0.024	0.950	0.955	0.951	0.948	0.948
β_{10}	0.026	0.034	0.026	0.026	0.026	0.957	0.955	0.956	0.959	0.957
β_{11}	0.026	0.035	0.026	0.026	0.026	0.954	0.950	0.955	0.954	0.956
β_{12}	0.026	0.034	0.026	0.026	0.026	0.955	0.960	0.955	0.954	0.953
β_{13}	0.029	0.037	0.029	0.029	0.029	0.952	0.944	0.957	0.953	0.952
β_{14}	0.036	0.048	0.036	0.036	0.036	0.956	0.955	0.953	0.956	0.958
β_{15}	0.024	0.032	0.024	0.024	0.024	0.948	0.953	0.948	0.949	0.949
β_{16}	0.025	0.033	0.025	0.025	0.025	0.927	0.949	0.929	0.929	0.932
β_{17}	0.030	0.039	0.030	0.030	0.030	0.961	0.946	0.959	0.959	0.960
β_{18}	0.028	0.036	0.028	0.028	0.028	0.945	0.946	0.949	0.945	0.946
β_{19}	0.023	0.031	0.023	0.023	0.023	0.961	0.951	0.963	0.960	0.960
β_{20}	0.026	0.035	0.026	0.026	0.026	0.956	0.959	0.958	0.961	0.958
Item category cutoff										
$\kappa_{19,1}$	0.020	0.028	0.020	0.020	0.020	0.957	0.958	0.956	0.958	0.956
$\kappa_{19,2}$	0.029	0.040	0.029	0.029	0.029	0.945	0.944	0.947	0.945	0.946
$\kappa_{20,1}$	0.028	0.038	0.028	0.028	0.028	0.947	0.946	0.949	0.949	0.950
$\kappa_{20,2}$	0.037	0.052	0.037	0.037	0.037	0.948	0.949	0.949	0.949	0.949

Notes: $G = 2$; $C = 20$; $N = 4000$; $J = 20$; $n_{iter} = 20000 + 5000$. RMSE = root mean square error; BD = before deletion; CC = complete cases; IBM = multiple imputation before modeling based on observed data; DART = data augmentation using sequential recursive partitioning based on all data and latent parameters. DART-m = data augmentation using sequential recursive partitioning based on the sufficient statistics θ and ω.

Table 7: Simulation study (Scenario 4 with $X_1 = 17\%$, $X_2 = 28\%$, and overall = 40% missings) - True parameter values, mean posterior medians and standard deviations of item characteristics over 1000 replications obtained from BD, CC, IBM and DART

		Average					Averaged standard deviation				
	True	BD	CC	IBM	DART	DART-m	BD	CC	IBM	DART	DART-m
Item discrimination											
α_1	1.017	1.017	1.017	1.017	1.017	1.017	0.031	0.040	0.031	0.031	0.031
α_2	0.964	0.966	0.966	0.966	0.966	0.966	0.029	0.038	0.029	0.029	0.029
α_3	1.326	1.326	1.325	1.325	1.326	1.326	0.042	0.054	0.042	0.042	0.042
α_4	1.080	1.081	1.082	1.081	1.081	1.081	0.034	0.044	0.034	0.034	0.034
α_5	0.867	0.867	0.867	0.867	0.867	0.867	0.027	0.035	0.027	0.027	0.027
α_6	0.979	0.980	0.980	0.980	0.980	0.980	0.031	0.040	0.031	0.031	0.031
α_7	0.775	0.775	0.776	0.775	0.775	0.775	0.025	0.032	0.025	0.025	0.025
α_8	1.095	1.097	1.098	1.097	1.097	1.097	0.036	0.046	0.036	0.036	0.036
α_9	0.850	0.849	0.849	0.849	0.849	0.849	0.026	0.034	0.026	0.026	0.026
α_{10}	1.164	1.164	1.168	1.164	1.164	1.164	0.036	0.046	0.036	0.036	0.036
α_{11}	1.111	1.113	1.112	1.113	1.113	1.113	0.034	0.045	0.034	0.034	0.034
α_{12}	0.784	0.784	0.787	0.784	0.784	0.784	0.026	0.034	0.026	0.026	0.026
α_{13}	1.107	1.109	1.109	1.109	1.109	1.109	0.034	0.045	0.034	0.034	0.034
α_{14}	1.412	1.414	1.415	1.414	1.414	1.413	0.047	0.061	0.047	0.047	0.047
α_{15}	0.917	0.918	0.917	0.918	0.918	0.918	0.028	0.037	0.028	0.028	0.028
α_{16}	0.779	0.781	0.781	0.781	0.781	0.781	0.025	0.033	0.025	0.025	0.025
α_{17}	0.841	0.840	0.841	0.841	0.840	0.840	0.029	0.037	0.029	0.029	0.029
α_{18}	1.119	1.120	1.118	1.119	1.120	1.120	0.034	0.044	0.034	0.034	0.034
α_{19}	0.865	0.865	0.865	0.865	0.865	0.865	0.023	0.030	0.023	0.023	0.023
α_{20}	1.261	1.262	1.263	1.262	1.262	1.262	0.030	0.039	0.030	0.030	0.030
Item difficulty											
β_1	−0.070	−0.071	−0.070	−0.071	−0.071	−0.071	0.025	0.032	0.025	0.025	0.025
β_2	−0.082	−0.084	−0.083	−0.084	−0.084	−0.084	0.025	0.032	0.025	0.025	0.025
β_3	−0.196	−0.198	−0.198	−0.198	−0.198	−0.198	0.028	0.035	0.028	0.028	0.028
β_4	−0.375	−0.376	−0.375	−0.376	−0.376	−0.376	0.026	0.033	0.026	0.026	0.026
β_5	−0.237	−0.237	−0.238	−0.237	−0.237	−0.237	0.024	0.031	0.024	0.024	0.024
β_6	−0.466	−0.466	−0.467	−0.466	−0.466	−0.466	0.026	0.033	0.026	0.026	0.026
β_7	−0.327	−0.328	−0.328	−0.328	−0.328	−0.328	0.024	0.030	0.024	0.024	0.024
β_8	0.867	0.869	0.870	0.869	0.869	0.869	0.034	0.043	0.034	0.034	0.034
β_9	−0.166	−0.167	−0.167	−0.167	−0.167	−0.167	0.024	0.031	0.024	0.024	0.024
β_{10}	0.008	0.006	0.006	0.006	0.006	0.006	0.026	0.034	0.026	0.026	0.026
β_{11}	−0.252	−0.251	−0.253	−0.251	−0.251	−0.251	0.026	0.033	0.026	0.026	0.026
β_{12}	−0.644	−0.645	−0.646	−0.645	−0.645	−0.645	0.025	0.033	0.025	0.025	0.025
β_{13}	0.522	0.522	0.521	0.522	0.522	0.522	0.029	0.037	0.029	0.029	0.029
β_{14}	0.858	0.858	0.859	0.858	0.858	0.857	0.037	0.047	0.037	0.037	0.037
β_{15}	0.032	0.031	0.030	0.031	0.031	0.031	0.025	0.031	0.025	0.025	0.025
β_{16}	−0.340	−0.340	−0.340	−0.340	−0.340	−0.340	0.024	0.030	0.024	0.024	0.024
β_{17}	0.887	0.887	0.889	0.887	0.887	0.887	0.031	0.039	0.031	0.031	0.031
β_{18}	0.301	0.301	0.300	0.301	0.301	0.301	0.027	0.035	0.027	0.027	0.027
β_{19}	0.101	0.101	0.101	0.101	0.101	0.101	0.024	0.031	0.024	0.024	0.024
β_{20}	−0.412	−0.412	−0.412	−0.412	−0.412	−0.412	0.027	0.035	0.027	0.027	0.027
Item category cutoff											
$\kappa_{19,1}$	0.500	0.500	0.500	0.500	0.500	0.500	0.020	0.026	0.020	0.020	0.020
$\kappa_{19,2}$	1.000	0.999	1.001	1.000	0.999	0.999	0.028	0.036	0.028	0.028	0.028
$\kappa_{20,1}$	0.700	0.699	0.700	0.699	0.699	0.699	0.028	0.036	0.028	0.028	0.028
$\kappa_{20,2}$	1.400	1.399	1.399	1.399	1.399	1.399	0.037	0.048	0.037	0.037	0.037

Notes: $G = 2$; $C = 20$; $N = 4000$; $J = 20$; $n_{iter} = 20000 + 5000$. BD = before deletion; CC = complete cases; IBM = multiple imputation before modeling based on observed data; DART = data augmentation using sequential recursive partitioning based on all data and latent parameters. DART-m = data augmentation using sequential recursive partitioning based on the sufficient statistics θ and ω.

Table 8: Simulation study (Scenario 4 with $X_1 = 17\%$, $X_2 = 28\%$, and overall = 40% missings) - RMSEs and coverage ratios of item characteristics over 1000 replications obtained from BD, CC, IBM and DART

	RMSE					Coverage				
	BD	CC	IBM	DART	DART-m	BD	CC	IBM	DART	DART-m
Item discrimination										
α_1	0.030	0.039	0.030	0.030	0.030	0.954	0.962	0.951	0.951	0.954
α_2	0.030	0.039	0.030	0.030	0.030	0.941	0.951	0.940	0.938	0.940
α_3	0.042	0.056	0.042	0.042	0.042	0.951	0.947	0.949	0.950	0.950
α_4	0.034	0.043	0.034	0.034	0.034	0.946	0.954	0.947	0.948	0.944
α_5	0.028	0.036	0.028	0.028	0.028	0.946	0.942	0.942	0.947	0.946
α_6	0.030	0.039	0.030	0.030	0.030	0.953	0.963	0.954	0.954	0.952
α_7	0.026	0.033	0.026	0.026	0.026	0.937	0.944	0.936	0.937	0.938
α_8	0.035	0.045	0.035	0.035	0.035	0.957	0.953	0.953	0.951	0.954
α_9	0.026	0.034	0.026	0.026	0.026	0.955	0.962	0.956	0.954	0.954
α_{10}	0.035	0.046	0.035	0.035	0.035	0.958	0.951	0.957	0.955	0.956
α_{11}	0.035	0.045	0.035	0.035	0.035	0.941	0.943	0.942	0.942	0.940
α_{12}	0.027	0.035	0.027	0.027	0.027	0.937	0.951	0.937	0.940	0.938
α_{13}	0.034	0.045	0.034	0.034	0.034	0.955	0.948	0.953	0.956	0.954
α_{14}	0.047	0.063	0.047	0.047	0.047	0.941	0.942	0.943	0.942	0.944
α_{15}	0.028	0.037	0.028	0.028	0.028	0.947	0.947	0.945	0.947	0.946
α_{16}	0.025	0.033	0.025	0.025	0.025	0.958	0.936	0.955	0.955	0.957
α_{17}	0.028	0.037	0.029	0.029	0.029	0.954	0.951	0.954	0.954	0.953
α_{18}	0.035	0.046	0.035	0.036	0.035	0.942	0.943	0.938	0.936	0.941
α_{19}	0.023	0.030	0.023	0.023	0.023	0.952	0.951	0.954	0.952	0.952
α_{20}	0.030	0.040	0.030	0.030	0.030	0.958	0.948	0.955	0.958	0.957
Item difficulty										
β_1	0.024	0.031	0.024	0.024	0.024	0.956	0.966	0.957	0.956	0.955
β_2	0.024	0.031	0.024	0.024	0.024	0.955	0.957	0.956	0.957	0.956
β_3	0.029	0.037	0.029	0.029	0.029	0.936	0.933	0.937	0.939	0.939
β_4	0.025	0.033	0.025	0.025	0.025	0.957	0.954	0.959	0.955	0.958
β_5	0.023	0.030	0.023	0.023	0.023	0.958	0.952	0.957	0.958	0.959
β_6	0.025	0.032	0.025	0.025	0.025	0.964	0.956	0.964	0.963	0.963
β_7	0.024	0.030	0.024	0.024	0.024	0.951	0.947	0.951	0.952	0.952
β_8	0.033	0.043	0.033	0.033	0.033	0.957	0.947	0.956	0.955	0.955
β_9	0.024	0.030	0.024	0.024	0.024	0.946	0.944	0.947	0.946	0.948
β_{10}	0.026	0.033	0.026	0.026	0.026	0.957	0.957	0.958	0.957	0.957
β_{11}	0.026	0.033	0.026	0.026	0.026	0.957	0.951	0.952	0.954	0.956
β_{12}	0.026	0.032	0.026	0.026	0.026	0.956	0.951	0.956	0.953	0.956
β_{13}	0.029	0.037	0.029	0.029	0.029	0.953	0.952	0.954	0.956	0.956
β_{14}	0.036	0.048	0.037	0.036	0.036	0.958	0.947	0.958	0.958	0.960
β_{15}	0.024	0.030	0.024	0.024	0.024	0.948	0.954	0.949	0.948	0.953
β_{16}	0.025	0.031	0.025	0.025	0.025	0.931	0.948	0.931	0.932	0.932
β_{17}	0.030	0.037	0.030	0.030	0.030	0.959	0.959	0.961	0.959	0.958
β_{18}	0.028	0.035	0.028	0.028	0.028	0.950	0.952	0.946	0.947	0.947
β_{19}	0.023	0.030	0.023	0.023	0.023	0.960	0.958	0.961	0.959	0.962
β_{20}	0.026	0.033	0.026	0.026	0.026	0.958	0.959	0.957	0.959	0.956
Item category cutoff										
$\kappa_{19,1}$	0.020	0.026	0.020	0.020	0.020	0.956	0.952	0.955	0.957	0.958
$\kappa_{19,2}$	0.029	0.036	0.029	0.029	0.029	0.946	0.949	0.947	0.943	0.947
$\kappa_{20,1}$	0.028	0.035	0.028	0.028	0.028	0.948	0.955	0.949	0.950	0.951
$\kappa_{20,2}$	0.037	0.047	0.037	0.037	0.037	0.952	0.952	0.949	0.954	0.953

Notes: $G = 2$; $C = 20$; $N = 4000$; $J = 20$; $n_{iter} = 20000 + 5000$. RMSE = root mean square error; BD = before deletion; CC = complete cases; IBM = multiple imputation before modeling based on observed data; DART = data augmentation using sequential recursive partitioning based on all data and latent parameters. DART-m = data augmentation using sequential recursive partitioning based on the sufficient statistics θ and ω.

ESTIMATION OF ITEM PARAMETERS WITHIN EMPIRICAL ILLUSTRATION

Table 9: NEPS grade 9, mathematical competencies – Item parameter estimates of model I

Item discrimination			Item difficulty			Item category cutoff		
α_1	0.933	(0.023)	β_1	−0.210	(0.011)	$\kappa_{3,2}$	0.631	(0.013)
α_2	0.971	(0.022)	β_2	0.149	(0.011)	$\kappa_{3,3}$	1.816	(0.018)
α_3	0.939	(0.019)	β_3	−1.239	(0.015)	$\kappa_{16,2}$	1.244	(0.017)
α_4	1.048	(0.029)	β_4	1.694	(0.023)	$\kappa_{16,3}$	1.853	(0.019)
α_5	1.033	(0.025)	β_5	−0.297	(0.012)			
α_6	0.698	(0.020)	β_6	0.133	(0.011)			
α_7	1.351	(0.032)	β_7	−0.711	(0.013)			
α_8	0.830	(0.020)	β_8	0.383	(0.012)			
α_9	1.245	(0.027)	β_9	−0.385	(0.012)			
α_{10}	1.163	(0.024)	β_{10}	0.078	(0.012)			
α_{11}	0.649	(0.019)	β_{11}	0.638	(0.012)			
α_{12}	1.011	(0.022)	β_{12}	0.290	(0.012)			
α_{13}	0.786	(0.022)	β_{13}	−0.346	(0.011)			
α_{14}	0.826	(0.021)	β_{14}	0.863	(0.013)			
α_{15}	1.020	(0.031)	β_{15}	−1.103	(0.015)			
α_{16}	0.735	(0.018)	β_{16}	−1.403	(0.016)			
α_{17}	1.209	(0.027)	β_{17}	0.878	(0.017)			
α_{18}	0.937	(0.022)	β_{18}	0.332	(0.012)			
α_{19}	1.501	(0.032)	β_{19}	−0.280	(0.013)			
α_{20}	1.147	(0.024)	β_{20}	0.318	(0.012)			
α_{21}	1.383	(0.029)	β_{21}	−0.002	(0.012)			
α_{22}	1.115	(0.024)	β_{22}	0.220	(0.012)			

Notes: $C = 532$; $N = 14320$; $N_{CC} = 6748$; $J = 22$. Median and standard deviation of the posterior distribution are reported.

Table 10: NEPS grade 9, mathematical competencies – Item parameter estimates of model II

Item discrimination			Item difficulty			Item category cutoff		
α_1	0.929	(0.022)	β_1	−0.211	(0.011)	$\kappa_{3,2}$	0.632	(0.013)
α_2	0.963	(0.021)	β_2	0.147	(0.011)	$\kappa_{3,3}$	1.821	(0.019)
α_3	0.950	(0.019)	β_3	−1.241	(0.015)	$\kappa_{16,2}$	1.246	(0.017)
α_4	1.053	(0.029)	β_4	1.700	(0.023)	$\kappa_{16,3}$	1.858	(0.019)
α_5	1.042	(0.024)	β_5	−0.298	(0.012)			
α_6	0.706	(0.019)	β_6	0.133	(0.011)			
α_7	1.334	(0.032)	β_7	−0.710	(0.013)			
α_8	0.829	(0.020)	β_8	0.382	(0.012)			
α_9	1.237	(0.027)	β_9	−0.385	(0.012)			
α_{10}	1.151	(0.024)	β_{10}	0.076	(0.012)			
α_{11}	0.651	(0.019)	β_{11}	0.639	(0.012)			
α_{12}	1.004	(0.022)	β_{12}	0.288	(0.012)			
α_{13}	0.789	(0.021)	β_{13}	−0.346	(0.011)			
α_{14}	0.835	(0.021)	β_{14}	0.866	(0.013)			
α_{15}	1.020	(0.032)	β_{15}	−1.103	(0.015)			
α_{16}	0.744	(0.018)	β_{16}	−1.405	(0.016)			
α_{17}	1.216	(0.027)	β_{17}	0.886	(0.017)			
α_{18}	0.934	(0.021)	β_{18}	0.331	(0.012)			
α_{19}	1.491	(0.031)	β_{19}	−0.281	(0.012)			
α_{20}	1.146	(0.024)	β_{20}	0.317	(0.012)			
α_{21}	1.382	(0.028)	β_{21}	−0.003	(0.012)			
α_{22}	1.105	(0.024)	β_{22}	0.218	(0.012)			

Notes: $C = 532$; $N = 14320$; $N_{CC} = 7708$; $J = 22$. Median and standard deviation of the posterior distribution are reported.

MONITORING CONVERGENCE

Convergence of the MCMC output has been monitored via the statistics suggested by Geweke (1992) and Gelman et al. (2013) given as follows.

The Geweke statistic tests for the equality of the sample mean at the begin and the end of the sampled sequence (single chain) with

$$CD = \frac{\overline{\psi}_A - \overline{\psi}_B}{\sqrt{\frac{S_A^2}{R_A} + \frac{S_B^2}{R_B}}}.$$

A refers to the first 20% (i.e. R_A) and B to the last 50% (i.e. R_B) iterations of the Gibbs sequence after burn-in. Further, $\bar{\psi}_A$, $\bar{\psi}_B$, S_A, and S_B correspond to the arithmetic means and autocorrelation robust variance estimates of the corresponding subsamples.

The Gelman-Rubin statistic, also referred to as potential scale reduction factor, is based on several chains. It assesses for each single parameter the ratio between total variation and variation within the set of Q drawn MCMC trajectories, i.e.

$$\widehat{R} = \sqrt{\frac{\widehat{\text{var}}^+}{W}},$$

where $\widehat{\text{var}}^+ = \frac{R-1}{R}W + \frac{1}{R}B$, $B = \frac{R}{Q-1}\sum_{q=1}^{Q}(\overline{\psi}_q - \overline{\psi})(\overline{\psi}_q - \overline{\psi})'$, $W = \frac{1}{Q}\sum_{q=1}^{Q}\frac{1}{R-1}\sum_{r=1}^{R}(\psi_q^{(r)} - \overline{\psi}_q)(\psi_q^{(r)} - \overline{\psi}_q)'$ and $\psi_q^{(r)}$ denoting the r-th sampled value of a parameter or a parameter vector in chain q, $\overline{\psi}_q$ the corresponding mean, and $\overline{\psi} = \frac{1}{Q}\sum_{q=1}^{Q}\overline{\psi}_q$ the overarching mean.

The multivariate version allowing for jointly monitoring the convergence of all parameters within a model is given as

$$\widehat{R}_M = \sqrt{\frac{R-1}{R} + \frac{\lambda_{\max}(W^{-1}B)}{R}},$$

where $\lambda_{\max}(W^{-1}B)$ refers to the largest eigenvalue of the matrix $W^{-1}B$.

As typical in hierarchical contexts and when accept-reject sampling is used, quite high autocorrelation is present within the sequences of single parameters. Following Gelman et al. (2013), we thus also report the effective MCMC sample sizes

$$ESS = \frac{QR}{1 + 2\sum_{t=1}^{T}\hat{\rho}_t}.$$

Here $\hat{\rho}_t = 1 - \frac{V_t}{2\widehat{\text{var}}^+}$ with $V_t = \frac{1}{Q(R-t)}\sum_{q=1}^{Q}\sum_{r=t+1}^{R}(\psi_q^{(r)} - \psi_q^{(r-t)})(\psi_q^{(r)} - \psi_q^{(r-t)})'$ denotes the estimated autocorrelation at lag t. T is the first odd positive integer for which $\hat{\rho}_{T+1} + \hat{\rho}_{T+2}$ is negative. As suggested by Gelman et al. (2013) the potential scale reduction factor $\widehat{R}$ and the effective sample size ESS are computed on split chains, therefor each chain was split in half, which leads to twice the number of chains. For both, simulation study and empirical applications we have sampled five chains.[21] Gelman et al. (2013) suggest as a rule of thumb that the ESS should be at least five times the number of split chains, which

[21] The reported convergence diagnostics for simulated data are based on the first of the 1000 simulated data sets.

in our case would be $ESS > 50$. This threshold is reached for all simulation studies and empirical applications. To account for the model complexity, as in our case, Vehtari, Gelman, Simpson, Carpenter, and Bürkner (2021) suggest to set a higher threshold at a minimum of 50 times the number of split chains, corresponding to $ESS > 500$ in our setup. Within the empirical illustrations the effective samples size per parameter are all well beyond this threshold. The empirical illustrations and the data generating process of the simulation studies differ with regard to the degree of heterogeneity related to the hierarchical structure captured in terms of the variances. Whereas in the empirical illustrations only modest heterogeneity prevails with corresponding sufficient effective sample sizes, the simulation study has incorporated more heterogeneity, i.e. higher variances σ_g^2 and ν_g^2, $g = 1, \ldots, G$. These higher levels of heterogeneity correspond to high autocorrelation in the intercepts $\gamma_{0,g}$ $g = 1, \ldots, G$, resulting in only small effective sample sizes. Please note that the sampling of the intercepts is closely related to the sampling of the random effects. In fact the draws of the intercepts and the draws of the random effects are strongly negatively correlated. This resembles the close interdependence of the fix intercepts $\gamma_{0,g}$, $g = 1, \ldots, G$ and the random intercepts ω_c, $c = 1, \ldots, C$ implied by the identifying assumption that the random intercepts have a priori expectations of zero. Both kinds of intercepts are sampled conditional on each other and thus contribute both towards the exploration of the parameter space. However, the conditional sampling process shows high state dependence and thus high autocorrelation, as a movement in the intercepts $\gamma_{0,g}$, $g = 1, \ldots, G$ affect all random intercepts and vice versa. However, when looking at the reparameterization of the overall conditional mean values, i.e. $\gamma_{0,L_i} + \omega_{S_i}$, the effective samples sizes are again well above the recommended threshold values, see Table (13), as the high negative correlation of the draws compensates the high autocorrelation. We also report the Monte Carlo error of parameter estimates, see Tables (14) to (18) for simulated data and Tables (25) and (26) for the empirical illustrations. The reported results document that the obtained inference is not plagued with simulation error.

Table 11: Simulation study - Convergence diagnostic (Geweke)

	BD	DART-m			
Scenario		1	2	3	4
$\gamma_{0,1}$	0.850	0.702	1.320	-1.218	0.375
$\gamma_{1,1}$	0.716	-0.822	1.124	0.139	1.022
$\gamma_{2,1}$	0.160	0.921	-0.743	-0.162	-0.724
$\gamma_{0,2}$	-0.942	1.680	-1.661	1.268	0.460
$\gamma_{1,2}$	0.524	1.173	2.069	-0.820	-0.926
$\gamma_{2,2}$	-0.209	0.550	-1.024	-1.138	1.170
σ_1^2	-1.110	-1.351	0.006	-0.356	-0.340
σ_2^2	-1.714	-1.025	-0.159	-0.414	-0.392
υ_1^2	-1.907	-0.264	-1.220	1.275	0.522
υ_2^2	-0.183	-1.845	2.774	-1.783	-2.854
α_1	-1.710	-0.915	-0.710	0.131	-0.079
α_2	0.850	-0.595	-1.285	0.661	-1.264
α_3	-1.703	-0.211	1.014	0.781	0.072
α_4	0.867	0.084	1.110	-0.293	1.066
α_5	1.563	0.628	-0.006	-0.575	-0.548
α_6	-1.436	0.400	0.186	-0.435	-0.025
α_7	0.140	0.221	-0.273	-1.133	-2.322
α_8	0.807	-0.482	-1.294	-0.353	0.279
α_9	-0.328	0.090	0.914	1.130	-0.645
α_{10}	-0.556	-2.086	0.764	1.113	-1.366
α_{11}	0.291	1.043	-1.277	3.063	0.373
α_{12}	0.873	0.737	0.523	-1.031	-0.726
α_{13}	0.798	1.100	0.343	0.684	1.441
α_{14}	0.181	-0.422	0.295	-0.664	-0.103
α_{15}	-1.022	0.248	0.404	-1.160	-0.408
α_{16}	0.369	0.684	-0.302	-0.147	0.186
α_{17}	-1.772	-1.031	-0.242	-0.234	0.999
α_{18}	1.439	0.616	-0.360	-1.806	-0.510
α_{19}	1.291	0.105	-0.802	-0.901	1.743
α_{20}	0.028	0.114	0.022	0.097	1.064
β_1	-1.847	-1.145	0.052	0.747	0.544
β_2	-1.040	-0.257	0.920	-0.008	-0.669
β_3	-0.533	1.563	-1.248	0.427	-0.377
β_4	1.700	-0.765	-1.038	0.489	-0.549
β_5	0.000	-1.155	0.106	-1.345	1.172
β_6	1.006	-1.883	0.522	1.324	1.586
β_7	0.891	0.798	-0.563	1.213	-2.043
β_8	0.698	-0.375	-0.636	-0.293	-0.053
β_9	0.176	-0.186	1.109	-0.413	-0.698
β_{10}	0.236	-0.757	1.202	-0.314	-1.583
β_{11}	0.180	-0.512	1.763	2.030	-1.335
β_{12}	-0.618	0.664	0.848	1.169	0.493
β_{13}	0.597	1.585	0.184	0.038	0.582
β_{14}	0.311	-0.375	0.497	-0.580	-0.524
β_{15}	-1.350	1.116	-1.472	0.274	1.698
β_{16}	-1.639	1.210	0.410	0.065	-1.035
β_{17}	-1.735	-0.529	0.345	-0.208	0.690
β_{18}	2.119	0.536	-0.524	-1.885	0.034
β_{19}	-0.184	0.354	-1.211	-1.191	1.781
β_{20}	-0.967	0.329	-1.470	0.375	-0.032
$\kappa_{19,1}$	0.541	-0.572	1.524	0.348	0.117
$\kappa_{19,2}$	0.885	0.262	0.414	0.218	0.354
$\kappa_{20,1}$	0.313	0.782	1.201	0.569	0.755
$\kappa_{20,2}$	0.463	0.103	0.861	0.043	0.732

Notes: Geweke statistic is calculated using the first MCMC trajectory with 20000 iterations after a burn in of 5000, assessing equality of means for the first 20% and last 50% of the MCMC trajectory. For all scenarios less then 5% of the statistics are above the 95% critical value of $|\pm 1.96|$.

Table 12: Simulation study - Convergence diagnostic (Gelman-Rubin $\hat{R}$ and ESS)

	BD		DART-m							
			Scenario 1		Scenario 2		Scenario 3		Scenario 4	
ψ	$\widehat{R}$ (UCB)	ESS	$\widehat{R}$ (UCB)	ESS	$\widehat{R}$ (UCB)	ESS	$\widehat{R}$ (UCB)	ESS	$\widehat{R}$ (UCB)	ESS
$\gamma_{0,1}$	1.02 (1.06)	274	1.01 (1.04)	239	1.03 (1.07)	239	1.03 (1.07)	177	1.01 (1.03)	232
$\gamma_{1,1}$	1.00 (1.00)	25673	1.01 (1.01)	3398	1.01 (1.01)	10890	1.00 (1.00)	24906	1.00 (1.00)	23354
$\gamma_{2,1}$	1.00 (1.00)	11423	1.00 (1.00)	9589	1.00 (1.00)	11871	1.00 (1.00)	10696	1.00 (1.00)	10193
$\gamma_{0,2}$	1.06 (1.18)	67	1.02 (1.06)	186	1.02 (1.06)	198	1.04 (1.11)	131	1.06 (1.14)	142
$\gamma_{1,2}$	1.00 (1.00)	26712	1.00 (1.00)	24525	1.01 (1.02)	8259	1.01 (1.01)	1948	1.00 (1.00)	24242
$\gamma_{2,2}$	1.00 (1.00)	8979	1.00 (1.00)	9694	1.00 (1.00)	8896	1.00 (1.00)	8361	1.00 (1.00)	8841
σ_1^2	1.00 (1.00)	5785	1.00 (1.00)	6637	1.00 (1.00)	7177	1.00 (1.00)	6636	1.00 (1.00)	6074
σ_2^2	1.00 (1.00)	6857	1.00 (1.00)	7397	1.00 (1.00)	7494	1.00 (1.00)	7599	1.00 (1.00)	7278
υ_1^2	1.00 (1.00)	6630	1.00 (1.00)	6082	1.00 (1.00)	5902	1.00 (1.00)	3336	1.00 (1.00)	4609
υ_2^2	1.00 (1.01)	1591	1.00 (1.00)	8248	1.00 (1.00)	7812	1.00 (1.00)	7335	1.00 (1.00)	2500
α_1	1.00 (1.00)	6399	1.00 (1.00)	6733	1.00 (1.00)	6171	1.00 (1.00)	6331	1.00 (1.00)	6208
α_2	1.00 (1.00)	6775	1.00 (1.00)	6650	1.00 (1.00)	6338	1.00 (1.00)	6690	1.00 (1.00)	6990
α_3	1.00 (1.00)	2927	1.00 (1.00)	3342	1.00 (1.00)	2898	1.00 (1.01)	3305	1.00 (1.00)	3147
α_4	1.00 (1.00)	4909	1.00 (1.00)	5448	1.00 (1.00)	5116	1.00 (1.00)	5175	1.00 (1.00)	5433
α_5	1.00 (1.00)	8986	1.00 (1.00)	8776	1.00 (1.00)	8446	1.00 (1.00)	8751	1.00 (1.00)	8858
α_6	1.00 (1.00)	5509	1.00 (1.00)	6557	1.00 (1.00)	6085	1.00 (1.00)	5924	1.00 (1.00)	5581
α_7	1.00 (1.00)	11028	1.00 (1.00)	10098	1.00 (1.00)	10868	1.00 (1.00)	11025	1.00 (1.00)	10471
α_8	1.00 (1.00)	4599	1.00 (1.00)	4863	1.00 (1.00)	4389	1.00 (1.00)	4463	1.00 (1.01)	4039
α_9	1.00 (1.00)	10254	1.00 (1.00)	9309	1.00 (1.00)	10104	1.00 (1.00)	10355	1.00 (1.00)	9617
α_{10}	1.00 (1.00)	5090	1.00 (1.00)	4698	1.00 (1.00)	5269	1.00 (1.00)	5167	1.00 (1.00)	5005
α_{11}	1.00 (1.00)	4768	1.00 (1.00)	4444	1.00 (1.01)	4694	1.00 (1.00)	4842	1.00 (1.00)	4610
α_{12}	1.00 (1.00)	8435	1.00 (1.00)	7978	1.00 (1.00)	8546	1.00 (1.00)	8496	1.00 (1.00)	8102
α_{13}	1.00 (1.00)	4256	1.00 (1.00)	4122	1.00 (1.00)	4520	1.00 (1.00)	4569	1.00 (1.00)	4794
α_{14}	1.00 (1.00)	2545	1.00 (1.00)	2671	1.00 (1.00)	2822	1.00 (1.01)	2742	1.00 (1.01)	2619
α_{15}	1.00 (1.00)	7451	1.00 (1.00)	7449	1.00 (1.00)	7311	1.00 (1.00)	7244	1.00 (1.00)	7205
α_{16}	1.00 (1.00)	9824	1.00 (1.00)	9595	1.00 (1.00)	9489	1.00 (1.00)	9627	1.00 (1.00)	9050
α_{17}	1.00 (1.00)	6549	1.00 (1.00)	7215	1.00 (1.00)	6581	1.00 (1.00)	6093	1.00 (1.00)	6758
α_{18}	1.00 (1.00)	5357	1.00 (1.00)	5495	1.00 (1.00)	5027	1.00 (1.00)	5301	1.00 (1.00)	5372
α_{19}	1.00 (1.00)	10229	1.00 (1.00)	10496	1.00 (1.00)	11325	1.00 (1.00)	11363	1.00 (1.00)	10440
α_{20}	1.00 (1.00)	5881	1.00 (1.00)	6686	1.00 (1.00)	6173	1.00 (1.00)	6230	1.00 (1.00)	6493
β_1	1.00 (1.00)	20153	1.00 (1.00)	21307	1.00 (1.00)	21366	1.00 (1.00)	21228	1.00 (1.00)	19608
β_2	1.00 (1.00)	21465	1.00 (1.00)	21885	1.00 (1.00)	22100	1.00 (1.00)	21844	1.00 (1.00)	21279
β_3	1.00 (1.00)	17364	1.00 (1.00)	17837	1.00 (1.00)	17655	1.00 (1.00)	17643	1.00 (1.00)	17706
β_4	1.00 (1.00)	18233	1.00 (1.00)	17676	1.00 (1.00)	17478	1.00 (1.00)	17856	1.00 (1.00)	17212
β_5	1.00 (1.00)	22489	1.00 (1.00)	23946	1.00 (1.00)	23484	1.00 (1.00)	22821	1.00 (1.00)	23177
β_6	1.00 (1.00)	16426	1.00 (1.00)	16886	1.00 (1.00)	16926	1.00 (1.00)	17484	1.00 (1.00)	17162
β_7	1.00 (1.00)	24979	1.00 (1.00)	24621	1.00 (1.00)	26103	1.00 (1.00)	25562	1.00 (1.00)	24900
β_8	1.00 (1.00)	6701	1.00 (1.00)	6703	1.00 (1.00)	6504	1.00 (1.00)	6337	1.00 (1.00)	6012
β_9	1.00 (1.00)	26313	1.00 (1.00)	26115	1.00 (1.00)	26041	1.00 (1.00)	25710	1.00 (1.00)	24840
β_{10}	1.00 (1.00)	16651	1.00 (1.00)	17260	1.00 (1.00)	17413	1.00 (1.00)	17522	1.00 (1.00)	17644
β_{11}	1.00 (1.00)	18133	1.00 (1.00)	18486	1.00 (1.00)	19604	1.00 (1.00)	18359	1.00 (1.00)	20206
β_{12}	1.00 (1.00)	17511	1.00 (1.00)	17398	1.00 (1.00)	17722	1.00 (1.00)	18418	1.00 (1.00)	17923
β_{13}	1.00 (1.00)	7777	1.00 (1.00)	7845	1.00 (1.00)	8173	1.00 (1.00)	8476	1.00 (1.00)	8352
β_{14}	1.00 (1.00)	4093	1.00 (1.00)	4186	1.00 (1.00)	4221	1.00 (1.01)	4037	1.00 (1.00)	3811
β_{15}	1.00 (1.00)	21098	1.00 (1.00)	19635	1.00 (1.00)	20034	1.00 (1.00)	20236	1.00 (1.00)	20325
β_{16}	1.00 (1.00)	21794	1.00 (1.00)	24695	1.00 (1.00)	24004	1.00 (1.00)	24099	1.00 (1.00)	24012
β_{17}	1.00 (1.00)	8830	1.00 (1.00)	9365	1.00 (1.00)	8863	1.00 (1.00)	8676	1.00 (1.00)	9332
β_{18}	1.00 (1.00)	13257	1.00 (1.00)	12908	1.00 (1.00)	12518	1.00 (1.00)	12438	1.00 (1.00)	11582
β_{19}	1.00 (1.00)	21739	1.00 (1.00)	20985	1.00 (1.00)	20943	1.00 (1.00)	21725	1.00 (1.00)	21137
β_{20}	1.00 (1.00)	17199	1.00 (1.00)	17479	1.00 (1.00)	16325	1.00 (1.00)	17240	1.00 (1.00)	16768
$\kappa_{19,1}$	1.00 (1.00)	43113	1.00 (1.00)	40760	1.00 (1.00)	42974	1.00 (1.00)	42406	1.00 (1.00)	40905
$\kappa_{19,2}$	1.00 (1.00)	31164	1.00 (1.00)	30806	1.00 (1.00)	32048	1.00 (1.00)	31614	1.00 (1.00)	31384
$\kappa_{20,1}$	1.00 (1.00)	16823	1.00 (1.00)	19018	1.00 (1.00)	17807	1.00 (1.00)	17059	1.00 (1.00)	18212
$\kappa_{20,2}$	1.00 (1.00)	10019	1.00 (1.00)	12118	1.00 (1.00)	11494	1.00 (1.00)	11330	1.00 (1.00)	11985
$\widehat{R}_M$	1.08		1.04		1.05		1.01		1.03	

Notes: Gelman-Rubin statistic ($\hat{R}$) and effective sample size (ESS) are calculated using the first 20000 iterations after a burn in of 5000 for each of the five MCMC trajectories. The upper confidence bound is provided in parentheses (UCB).

Table 13: Simulation study - Convergence diagnostic (Gelman-Rubin $\hat{R}$ and ESS)

	BD		DART-m							
			Scenario 1		Scenario 2		Scenario 3		Scenario 4	
ψ	$\widehat{R}$ (UCB)	ESS	$\widehat{R}$ (UCB)	ESS	$\widehat{R}$ (UCB)	ESS	$\widehat{R}$ (UCB)	ESS	$\widehat{R}$ (UCB)	ESS
ω_1	1.02 (1.05)	293	1.01 (1.04)	256	1.03 (1.07)	254	1.03 (1.07)	189	1.01 (1.03)	251
ω_2	1.02 (1.06)	290	1.01 (1.04)	250	1.03 (1.07)	251	1.03 (1.07)	185	1.01 (1.03)	248
ω_3	1.02 (1.05)	294	1.01 (1.03)	253	1.03 (1.07)	257	1.03 (1.06)	193	1.01 (1.03)	251
ω_4	1.02 (1.06)	285	1.01 (1.04)	244	1.03 (1.07)	238	1.03 (1.07)	183	1.01 (1.03)	241
ω_5	1.02 (1.06)	284	1.01 (1.04)	246	1.03 (1.08)	237	1.03 (1.07)	184	1.01 (1.03)	241
ω_6	1.02 (1.06)	283	1.01 (1.04)	243	1.03 (1.07)	242	1.03 (1.07)	182	1.01 (1.03)	241
ω_7	1.02 (1.06)	285	1.01 (1.04)	246	1.03 (1.07)	244	1.03 (1.07)	182	1.01 (1.03)	241
ω_8	1.02 (1.06)	287	1.01 (1.04)	249	1.03 (1.07)	243	1.03 (1.07)	184	1.01 (1.03)	242
ω_9	1.02 (1.06)	287	1.01 (1.04)	250	1.03 (1.07)	248	1.03 (1.07)	183	1.01 (1.03)	244
ω_{10}	1.02 (1.05)	290	1.01 (1.04)	255	1.03 (1.07)	254	1.03 (1.07)	186	1.01 (1.03)	245
ω_{11}	1.06 (1.15)	69	1.02 (1.05)	194	1.02 (1.05)	208	1.04 (1.09)	136	1.05 (1.13)	147
ω_{12}	1.06 (1.16)	68	1.02 (1.05)	193	1.02 (1.06)	204	1.04 (1.09)	133	1.06 (1.14)	141
ω_{13}	1.06 (1.16)	68	1.02 (1.05)	191	1.02 (1.06)	204	1.04 (1.09)	133	1.06 (1.14)	143
ω_{14}	1.06 (1.16)	67	1.02 (1.05)	190	1.02 (1.06)	199	1.04 (1.09)	132	1.06 (1.14)	142
ω_{15}	1.06 (1.16)	68	1.02 (1.05)	192	1.02 (1.06)	201	1.04 (1.09)	134	1.06 (1.14)	143
ω_{16}	1.06 (1.15)	69	1.02 (1.05)	193	1.02 (1.06)	201	1.04 (1.08)	133	1.06 (1.13)	146
ω_{17}	1.06 (1.16)	68	1.02 (1.05)	191	1.02 (1.06)	199	1.04 (1.09)	132	1.06 (1.13)	141
ω_{18}	1.06 (1.15)	69	1.02 (1.05)	196	1.02 (1.06)	202	1.04 (1.08)	137	1.06 (1.13)	143
ω_{19}	1.06 (1.15)	69	1.02 (1.05)	199	1.02 (1.06)	207	1.04 (1.08)	137	1.05 (1.15)	148
ω_{20}	1.06 (1.15)	69	1.02 (1.05)	198	1.02 (1.06)	206	1.04 (1.09)	137	1.05 (1.13)	142
$\gamma_{0,1} + \omega_1$	1.00 (1.00)	12341	1.00 (1.00)	10055	1.00 (1.01)	13196	1.00 (1.00)	12736	1.00 (1.00)	11503
$\gamma_{0,1} + \omega_2$	1.00 (1.00)	11574	1.00 (1.00)	9793	1.00 (1.00)	11488	1.00 (1.00)	10729	1.00 (1.00)	10108
$\gamma_{0,1} + \omega_3$	1.00 (1.00)	4499	1.00 (1.01)	4887	1.00 (1.00)	7292	1.00 (1.00)	5224	1.00 (1.00)	4677
$\gamma_{0,1} + \omega_4$	1.00 (1.00)	20762	1.00 (1.00)	17265	1.00 (1.00)	19670	1.00 (1.00)	20632	1.00 (1.00)	19543
$\gamma_{0,1} + \omega_5$	1.00 (1.00)	24184	1.00 (1.00)	17703	1.00 (1.01)	21387	1.00 (1.00)	22555	1.00 (1.00)	21430
$\gamma_{0,1} + \omega_6$	1.00 (1.00)	42976	1.00 (1.00)	29625	1.00 (1.00)	32332	1.00 (1.00)	42546	1.00 (1.00)	35163
$\gamma_{0,1} + \omega_7$	1.00 (1.00)	29422	1.00 (1.00)	25254	1.00 (1.00)	24869	1.00 (1.00)	30622	1.00 (1.00)	28817
$\gamma_{0,1} + \omega_8$	1.00 (1.00)	31980	1.00 (1.00)	31793	1.00 (1.00)	28318	1.00 (1.00)	34936	1.00 (1.00)	33415
$\gamma_{0,1} + \omega_9$	1.00 (1.00)	56949	1.00 (1.00)	53168	1.00 (1.00)	50074	1.00 (1.00)	53919	1.00 (1.00)	55416
$\gamma_{0,1} + \omega_{10}$	1.00 (1.00)	38543	1.00 (1.00)	35508	1.00 (1.00)	32251	1.00 (1.00)	37595	1.00 (1.00)	38600
$\gamma_{0,2} + \omega_{11}$	1.00 (1.00)	31856	1.00 (1.00)	27947	1.00 (1.00)	30592	1.00 (1.00)	22886	1.00 (1.00)	30324
$\gamma_{0,2} + \omega_{12}$	1.00 (1.00)	33986	1.00 (1.00)	33482	1.00 (1.00)	33448	1.00 (1.00)	29801	1.00 (1.00)	32965
$\gamma_{0,2} + \omega_{13}$	1.00 (1.00)	50423	1.00 (1.00)	49507	1.00 (1.00)	43909	1.00 (1.00)	42336	1.00 (1.00)	47651
$\gamma_{0,2} + \omega_{14}$	1.00 (1.00)	35291	1.00 (1.00)	33586	1.00 (1.00)	31468	1.00 (1.00)	28215	1.00 (1.00)	32897
$\gamma_{0,2} + \omega_{15}$	1.00 (1.00)	21265	1.00 (1.00)	22192	1.00 (1.00)	20566	1.00 (1.00)	20396	1.00 (1.00)	21994
$\gamma_{0,2} + \omega_{16}$	1.00 (1.00)	10376	1.00 (1.00)	11021	1.00 (1.00)	10333	1.00 (1.00)	10107	1.00 (1.00)	11601
$\gamma_{0,2} + \omega_{17}$	1.00 (1.00)	17703	1.00 (1.00)	18002	1.00 (1.00)	15320	1.00 (1.00)	15650	1.00 (1.00)	16426
$\gamma_{0,2} + \omega_{18}$	1.00 (1.00)	9688	1.00 (1.00)	9525	1.00 (1.00)	8729	1.00 (1.00)	8427	1.00 (1.00)	8505
$\gamma_{0,2} + \omega_{19}$	1.00 (1.00)	9173	1.00 (1.00)	9261	1.00 (1.00)	8097	1.00 (1.00)	8351	1.00 (1.00)	8051
$\gamma_{0,2} + \omega_{20}$	1.00 (1.00)	9942	1.00 (1.00)	10558	1.00 (1.00)	9788	1.00 (1.00)	9446	1.00 (1.00)	10093

Notes: Gelman-Rubin statistic ($\hat{R}$) and effective sample size (ESS) are calculated using the first 20000 iterations after a burn in of 5000 for each of the five MCMC trajectories. The upper confidence bound is provided in parentheses (UCB).

Table 14: Simulation study (BD) - Monte Carlo error of estimates

	$m(\hat{\psi})$	$sd(\hat{\psi})$	$m(\hat{\sigma}_{\psi})$	$sd(\hat{\sigma}_{\psi})$		$m(\hat{\psi})$	$sd(\hat{\psi})$	$m(\hat{\sigma}_{\psi})$	$sd(\hat{\sigma}_{\psi})$
$\gamma_{0,1}$	-0.6702	0.0446	0.2677	0.0254	ω_1	-0.3950	0.0414	0.2765	0.0270
$\gamma_{1,1}$	0.3851	0.0004	0.0455	0.0004	ω_2	-0.8628	0.0432	0.2753	0.0271
$\gamma_{2,1}$	0.2013	0.0009	0.0470	0.0004	ω_3	-1.7043	0.0397	0.2767	0.0291
$\gamma_{0,2}$	1.1565	0.0751	0.2476	0.0297	ω_4	0.1823	0.0427	0.2715	0.0264
$\gamma_{1,2}$	0.1540	0.0004	0.0322	0.0001	ω_5	-0.1081	0.0423	0.2709	0.0252
$\gamma_{2,2}$	-0.1968	0.0002	0.0369	0.0002	ω_6	0.5805	0.0432	0.2714	0.0250
σ_1^2	0.6853	0.0007	0.0308	0.0003	ω_7	-0.3086	0.0429	0.2719	0.0260
σ_2^2	0.3372	0.0004	0.0170	0.0003	ω_8	1.6640	0.0438	0.2733	0.0236
υ_1^2	0.6748	0.0069	0.3192	0.0133	ω_9	0.7005	0.0444	0.2733	0.0248
υ_2^2	0.5059	0.0085	0.2479	0.0162	ω_{10}	0.3379	0.0440	0.2748	0.0247
α_1	1.0345	0.0009	0.0309	0.0004	ω_{11}	-0.8599	0.0737	0.2520	0.0290
α_2	0.9931	0.0010	0.0302	0.0004	ω_{12}	-0.4256	0.0737	0.2505	0.0292
α_3	1.3325	0.0021	0.0429	0.0008	ω_{13}	-1.1480	0.0752	0.2504	0.0293
α_4	1.0708	0.0011	0.0329	0.0005	ω_{14}	-0.5275	0.0744	0.2493	0.0298
α_5	0.8644	0.0004	0.0259	0.0003	ω_{15}	0.0961	0.0748	0.2490	0.0296
α_6	1.0069	0.0013	0.0309	0.0003	ω_{16}	0.7464	0.0741	0.2504	0.0306
α_7	0.7449	0.0005	0.0227	0.0004	ω_{17}	-0.2471	0.0732	0.2488	0.0303
α_8	1.0660	0.0011	0.0335	0.0006	ω_{18}	0.8908	0.0731	0.2504	0.0307
α_9	0.7895	0.0004	0.0238	0.0001	ω_{19}	0.9609	0.0732	0.2515	0.0312
α_{10}	1.1307	0.0004	0.0348	0.0005	ω_{20}	0.5480	0.0749	0.2513	0.0304
α_{11}	1.1498	0.0012	0.0356	0.0006	$\gamma_{0,1}+\omega_1$	-1.0661	0.0021	0.1035	0.0011
α_{12}	0.7865	0.0005	0.0249	0.0002	$\gamma_{0,1}+\omega_2$	-1.5334	0.0022	0.0970	0.0010
α_{13}	1.1612	0.0015	0.0359	0.0005	$\gamma_{0,1}+\omega_3$	-2.3741	0.0022	0.1045	0.0018
α_{14}	1.3968	0.0022	0.0458	0.0009	$\gamma_{0,1}+\omega_4$	-0.4880	0.0011	0.0788	0.0006
α_{15}	0.9525	0.0006	0.0285	0.0002	$\gamma_{0,1}+\omega_5$	-0.7789	0.0009	0.0741	0.0002
α_{16}	0.7829	0.0007	0.0240	0.0003	$\gamma_{0,1}+\omega_6$	-0.0889	0.0005	0.0675	0.0002
α_{17}	0.8291	0.0008	0.0277	0.0002	$\gamma_{0,1}+\omega_7$	-0.9783	0.0011	0.0679	0.0003
α_{18}	1.0993	0.0005	0.0336	0.0004	$\gamma_{0,1}+\omega_8$	0.9942	0.0013	0.0670	0.0006
α_{19}	0.8663	0.0006	0.0224	0.0003	$\gamma_{0,1}+\omega_9$	0.0315	0.0007	0.0638	0.0002
α_{20}	1.2838	0.0011	0.0307	0.0003	$\gamma_{0,1}+\omega_{10}$	-0.3310	0.0006	0.0661	0.0003
β_1	-0.0506	0.0002	0.0259	0.0003	$\gamma_{0,2}+\omega_{11}$	0.2977	0.0005	0.0520	0.0002
β_2	-0.0600	0.0002	0.0255	0.0001	$\gamma_{0,2}+\omega_{12}$	0.7318	0.0006	0.0516	0.0002
β_3	-0.2183	0.0008	0.0283	0.0002	$\gamma_{0,2}+\omega_{13}$	0.0096	0.0005	0.0492	0.0002
β_4	-0.4096	0.0005	0.0269	0.0002	$\gamma_{0,2}+\omega_{14}$	0.6310	0.0007	0.0532	0.0002
β_5	-0.2298	0.0002	0.0245	0.0002	$\gamma_{0,2}+\omega_{15}$	1.2541	0.0008	0.0559	0.0002
β_6	-0.4865	0.0003	0.0267	0.0003	$\gamma_{0,2}+\omega_{16}$	1.9063	0.0009	0.0653	0.0008
β_7	-0.3282	0.0003	0.0238	0.0002	$\gamma_{0,2}+\omega_{17}$	0.9111	0.0007	0.0620	0.0004
β_8	0.8444	0.0008	0.0339	0.0003	$\gamma_{0,2}+\omega_{18}$	2.0495	0.0011	0.0734	0.0005
β_9	-0.1618	0.0003	0.0239	0.0002	$\gamma_{0,2}+\omega_{19}$	2.1195	0.0008	0.0805	0.0005
β_{10}	0.0254	0.0003	0.0272	0.0002	$\gamma_{0,2}+\omega_{20}$	1.7082	0.0007	0.0820	0.0004
β_{11}	-0.2466	0.0005	0.0269	0.0002					
β_{12}	-0.6359	0.0004	0.0259	0.0002					
β_{13}	0.5832	0.0016	0.0315	0.0003					
β_{14}	0.8544	0.0010	0.0387	0.0007					
β_{15}	0.0605	0.0003	0.0255	0.0001					
β_{16}	-0.3770	0.0004	0.0244	0.0002					
β_{17}	0.8712	0.0007	0.0315	0.0003					
β_{18}	0.2929	0.0004	0.0280	0.0002					
β_{19}	0.0992	0.0004	0.0250	0.0002					
β_{20}	-0.4271	0.0007	0.0282	0.0002					
$\kappa_{19,1}$	0.5074	0.0003	0.0209	0.0001					
$\kappa_{19,2}$	0.9921	0.0006	0.0279	0.0001					
$\kappa_{20,1}$	0.7477	0.0010	0.0299	0.0002					
$\kappa_{20,2}$	1.4479	0.0018	0.0394	0.0003					

Notes: $m(\hat{\psi})$ reports the arithmetic mean of the parameter estimates from the five different chains, $sd(\hat{\psi})$ denotes the corresponding standard deviation, $m(\hat{\sigma}_{\psi})$ denotes the arithmetic mean of the parameter specific standard deviation from the different chains, $sd(\hat{\sigma}_{\psi})$ the corresponding standard deviation.

Table 15: Simulation study (DART-m, Scenario 1) - Monte Carlo error of estimates

	$m(\hat{\psi})$	$sd(\hat{\psi})$	$m(\hat{\sigma}_{\psi})$	$sd(\hat{\sigma}_{\psi})$		$m(\hat{\psi})$	$sd(\hat{\psi})$	$m(\hat{\sigma}_{\psi})$	$sd(\hat{\sigma}_{\psi})$
$\gamma_{0,1}$	-0.7097	0.0164	0.2830	0.0245	ω_1	-0.4789	0.0150	0.2909	0.0225
$\gamma_{1,1}$	0.2993	0.0071	0.0634	0.0019	ω_2	-0.9435	0.0160	0.2891	0.0222
$\gamma_{2,1}$	0.1060	0.0023	0.0599	0.0006	ω_3	-1.8250	0.0150	0.2922	0.0223
$\gamma_{0,2}$	1.1407	0.0367	0.2185	0.0302	ω_4	0.1503	0.0152	0.2854	0.0234
$\gamma_{1,2}$	0.1448	0.0021	0.0327	0.0001	ω_5	-0.1099	0.0153	0.2855	0.0238
$\gamma_{2,2}$	-0.2002	0.0009	0.0376	0.0004	ω_6	0.5926	0.0151	0.2857	0.0243
σ_1^2	0.6990	0.0012	0.0318	0.0003	ω_7	-0.2761	0.0165	0.2865	0.0240
σ_2^2	0.3371	0.0009	0.0170	0.0001	ω_8	1.7174	0.0157	0.2878	0.0234
υ_1^2	0.7437	0.0069	0.3550	0.0104	ω_9	0.7703	0.0156	0.2887	0.0238
υ_2^2	0.5000	0.0087	0.2341	0.0063	ω_{10}	0.4274	0.0150	0.2915	0.0238
α_1	1.0344	0.0013	0.0313	0.0006	ω_{11}	-0.8390	0.0365	0.2233	0.0297
α_2	0.9935	0.0008	0.0295	0.0006	ω_{12}	-0.4064	0.0366	0.2225	0.0301
α_3	1.3312	0.0014	0.0423	0.0007	ω_{13}	-1.1193	0.0372	0.2213	0.0299
α_4	1.0717	0.0011	0.0331	0.0005	ω_{14}	-0.5016	0.0371	0.2207	0.0296
α_5	0.8654	0.0003	0.0261	0.0002	ω_{15}	0.1209	0.0368	0.2212	0.0296
α_6	1.0077	0.0006	0.0308	0.0005	ω_{16}	0.7743	0.0360	0.2221	0.0291
α_7	0.7439	0.0004	0.0230	0.0002	ω_{17}	-0.2220	0.0369	0.2211	0.0293
α_8	1.0658	0.0013	0.0337	0.0003	ω_{18}	0.9203	0.0375	0.2240	0.0293
α_9	0.7896	0.0004	0.0238	0.0002	ω_{19}	0.9889	0.0358	0.2254	0.0288
α_{10}	1.1311	0.0008	0.0344	0.0005	ω_{20}	0.5815	0.0381	0.2251	0.0288
α_{11}	1.1480	0.0020	0.0353	0.0008	$\gamma_{0,1}+\omega_1$	-1.1892	0.0049	0.1203	0.0012
α_{12}	0.7862	0.0006	0.0248	0.0002	$\gamma_{0,1}+\omega_2$	-1.6526	0.0046	0.1081	0.0011
α_{13}	1.1609	0.0016	0.0356	0.0005	$\gamma_{0,1}+\omega_3$	-2.5366	0.0116	0.1209	0.0034
α_{14}	1.3986	0.0007	0.0449	0.0007	$\gamma_{0,1}+\omega_4$	-0.5600	0.0038	0.0858	0.0003
α_{15}	0.9531	0.0011	0.0284	0.0003	$\gamma_{0,1}+\omega_5$	-0.8200	0.0044	0.0815	0.0004
α_{16}	0.7836	0.0006	0.0238	0.0003	$\gamma_{0,1}+\omega_6$	-0.1178	0.0039	0.0717	0.0004
α_{17}	0.8290	0.0006	0.0275	0.0002	$\gamma_{0,1}+\omega_7$	-0.9857	0.0036	0.0712	0.0003
α_{18}	1.0981	0.0008	0.0335	0.0003	$\gamma_{0,1}+\omega_8$	1.0088	0.0034	0.0692	0.0003
α_{19}	0.8662	0.0005	0.0223	0.0002	$\gamma_{0,1}+\omega_9$	0.0616	0.0022	0.0658	0.0005
α_{20}	1.2831	0.0006	0.0303	0.0006	$\gamma_{0,1}+\omega_{10}$	-0.2821	0.0024	0.0692	0.0005
β_1	-0.0507	0.0005	0.0257	0.0003	$\gamma_{0,2}+\omega_{11}$	0.3010	0.0011	0.0525	0.0001
β_2	-0.0604	0.0003	0.0256	0.0003	$\gamma_{0,2}+\omega_{12}$	0.7334	0.0012	0.0518	0.0003
β_3	-0.2186	0.0008	0.0282	0.0003	$\gamma_{0,2}+\omega_{13}$	0.0204	0.0011	0.0495	0.0002
β_4	-0.4093	0.0003	0.0270	0.0003	$\gamma_{0,2}+\omega_{14}$	0.6387	0.0013	0.0538	0.0002
β_5	-0.2299	0.0002	0.0246	0.0001	$\gamma_{0,2}+\omega_{15}$	1.2607	0.0007	0.0564	0.0003
β_6	-0.4864	0.0002	0.0268	0.0003	$\gamma_{0,2}+\omega_{16}$	1.9158	0.0007	0.0652	0.0004
β_7	-0.3280	0.0002	0.0239	0.0002	$\gamma_{0,2}+\omega_{17}$	0.9178	0.0016	0.0629	0.0002
β_8	0.8441	0.0010	0.0337	0.0003	$\gamma_{0,2}+\omega_{18}$	2.0603	0.0011	0.0749	0.0007
β_9	-0.1618	0.0003	0.0239	0.0000	$\gamma_{0,2}+\omega_{19}$	2.1300	0.0021	0.0812	0.0010
β_{10}	0.0255	0.0003	0.0271	0.0003	$\gamma_{0,2}+\omega_{20}$	1.7220	0.0028	0.0831	0.0006
β_{11}	-0.2467	0.0007	0.0270	0.0003					
β_{12}	-0.6360	0.0004	0.0258	0.0002					
β_{13}	0.5826	0.0010	0.0314	0.0002					
β_{14}	0.8557	0.0010	0.0378	0.0005					
β_{15}	0.0610	0.0003	0.0255	0.0002					
β_{16}	-0.3768	0.0002	0.0242	0.0001					
β_{17}	0.8710	0.0012	0.0316	0.0003					
β_{18}	0.2924	0.0007	0.0282	0.0002					
β_{19}	0.0987	0.0005	0.0249	0.0001					
β_{20}	-0.4279	0.0006	0.0281	0.0002					
$\kappa_{19,1}$	0.5075	0.0004	0.0209	0.0002					
$\kappa_{19,2}$	0.9924	0.0006	0.0281	0.0002					
$\kappa_{20,1}$	0.7479	0.0004	0.0300	0.0002					
$\kappa_{20,2}$	1.4478	0.0005	0.0392	0.0002					

Notes: $m(\hat{\psi})$ reports the arithmetic mean of the parameter estimates from the five different chains, $sd(\hat{\psi})$ denotes the corresponding standard deviation, $m(\hat{\sigma}_{\psi})$ denotes the arithmetic mean of the parameter specific standard deviation from the different chains, $sd(\hat{\sigma}_{\psi})$ the corresponding standard deviation.

Table 16: Simulation study (DART-m, Scenario 2) - Monte Carlo error of estimates

	$m(\hat{\psi})$	$sd(\hat{\psi})$	$m(\hat{\sigma}_{\psi})$	$sd(\hat{\sigma}_{\psi})$		$m(\hat{\psi})$	$sd(\hat{\psi})$	$m(\hat{\sigma}_{\psi})$	$sd(\hat{\sigma}_{\psi})$
$\gamma_{0,1}$	-0.6264	0.0302	0.2773	0.0251	ω_1	-0.4622	0.0368	0.2893	0.0269
$\gamma_{1,1}$	0.2370	0.0104	0.0957	0.0024	ω_2	-0.8860	0.0352	0.2852	0.0244
$\gamma_{2,1}$	0.1766	0.0033	0.0633	0.0012	ω_3	-1.6489	0.0338	0.2904	0.0267
$\gamma_{0,2}$	1.1252	0.0337	0.2120	0.0199	ω_4	0.1408	0.0351	0.2789	0.0255
$\gamma_{1,2}$	0.1575	0.0047	0.0351	0.0002	ω_5	-0.1426	0.0353	0.2794	0.0253
$\gamma_{2,2}$	-0.1812	0.0011	0.0396	0.0006	ω_6	0.5564	0.0322	0.2792	0.0257
σ_1^2	0.7017	0.0015	0.0326	0.0002	ω_7	-0.3694	0.0316	0.2805	0.0247
σ_2^2	0.3373	0.0006	0.0170	0.0003	ω_8	1.6673	0.0326	0.2806	0.0248
υ_1^2	0.6766	0.0092	0.3233	0.0059	ω_9	0.7070	0.0310	0.2820	0.0247
υ_2^2	0.4901	0.0061	0.2284	0.0057	ω_{10}	0.3358	0.0309	0.2851	0.0243
α_1	1.0336	0.0017	0.0311	0.0005	ω_{11}	-0.8207	0.0348	0.2170	0.0194
α_2	0.9934	0.0008	0.0299	0.0004	ω_{12}	-0.3924	0.0367	0.2155	0.0199
α_3	1.3320	0.0014	0.0427	0.0005	ω_{13}	-1.1275	0.0345	0.2147	0.0197
α_4	1.0702	0.0004	0.0328	0.0005	ω_{14}	-0.5042	0.0380	0.2138	0.0194
α_5	0.8653	0.0005	0.0259	0.0003	ω_{15}	0.1198	0.0361	0.2142	0.0194
α_6	1.0072	0.0005	0.0309	0.0008	ω_{16}	0.7669	0.0372	0.2153	0.0191
α_7	0.7441	0.0004	0.0229	0.0002	ω_{17}	-0.2306	0.0374	0.2142	0.0191
α_8	1.0659	0.0017	0.0340	0.0002	ω_{18}	0.8993	0.0391	0.2164	0.0188
α_9	0.7901	0.0004	0.0238	0.0002	ω_{19}	0.9688	0.0373	0.2182	0.0187
α_{10}	1.1317	0.0008	0.0346	0.0006	ω_{20}	0.5534	0.0379	0.2183	0.0189
α_{11}	1.1482	0.0023	0.0354	0.0004	$\gamma_{0,1}+\omega_1$	-1.0926	0.0091	0.1337	0.0028
α_{12}	0.7869	0.0003	0.0247	0.0002	$\gamma_{0,1}+\omega_2$	-1.5155	0.0070	0.1169	0.0012
α_{13}	1.1623	0.0011	0.0358	0.0008	$\gamma_{0,1}+\omega_3$	-2.2784	0.0041	0.1271	0.0018
α_{14}	1.3969	0.0023	0.0452	0.0012	$\gamma_{0,1}+\omega_4$	-0.4878	0.0057	0.0907	0.0010
α_{15}	0.9521	0.0010	0.0285	0.0004	$\gamma_{0,1}+\omega_5$	-0.7704	0.0073	0.0886	0.0010
α_{16}	0.7838	0.0006	0.0240	0.0002	$\gamma_{0,1}+\omega_6$	-0.0708	0.0038	0.0777	0.0012
α_{17}	0.8292	0.0010	0.0278	0.0003	$\gamma_{0,1}+\omega_7$	-0.9973	0.0050	0.0772	0.0008
α_{18}	1.0984	0.0008	0.0330	0.0006	$\gamma_{0,1}+\omega_8$	1.0408	0.0033	0.0739	0.0007
α_{19}	0.8664	0.0006	0.0223	0.0003	$\gamma_{0,1}+\omega_9$	0.0813	0.0017	0.0681	0.0005
α_{20}	1.2819	0.0011	0.0301	0.0004	$\gamma_{0,1}+\omega_{10}$	-0.2904	0.0014	0.0734	0.0004
β_1	-0.0505	0.0003	0.0258	0.0002	$\gamma_{0,2}+\omega_{11}$	0.3047	0.0022	0.0529	0.0002
β_2	-0.0605	0.0002	0.0256	0.0001	$\gamma_{0,2}+\omega_{12}$	0.7318	0.0032	0.0525	0.0002
β_3	-0.2183	0.0003	0.0284	0.0002	$\gamma_{0,2}+\omega_{13}$	-0.0018	0.0020	0.0507	0.0004
β_4	-0.4091	0.0003	0.0270	0.0002	$\gamma_{0,2}+\omega_{14}$	0.6212	0.0038	0.0541	0.0004
β_5	-0.2304	0.0004	0.0246	0.0002	$\gamma_{0,2}+\omega_{15}$	1.2452	0.0029	0.0580	0.0006
β_6	-0.4861	0.0003	0.0268	0.0002	$\gamma_{0,2}+\omega_{16}$	1.8919	0.0030	0.0664	0.0005
β_7	-0.3282	0.0003	0.0237	0.0001	$\gamma_{0,2}+\omega_{17}$	0.8953	0.0027	0.0649	0.0003
β_8	0.8445	0.0014	0.0340	0.0003	$\gamma_{0,2}+\omega_{18}$	2.0252	0.0040	0.0766	0.0007
β_9	-0.1615	0.0004	0.0238	0.0001	$\gamma_{0,2}+\omega_{19}$	2.0936	0.0035	0.0839	0.0013
β_{10}	0.0255	0.0007	0.0269	0.0003	$\gamma_{0,2}+\omega_{20}$	1.6785	0.0037	0.0855	0.0011
β_{11}	-0.2468	0.0005	0.0270	0.0002					
β_{12}	-0.6357	0.0002	0.0258	0.0002					
β_{13}	0.5836	0.0006	0.0314	0.0002					
β_{14}	0.8539	0.0012	0.0379	0.0009					
β_{15}	0.0608	0.0003	0.0253	0.0002					
β_{16}	-0.3766	0.0004	0.0244	0.0001					
β_{17}	0.8714	0.0009	0.0315	0.0005					
β_{18}	0.2920	0.0005	0.0280	0.0001					
β_{19}	0.0988	0.0005	0.0248	0.0003					
β_{20}	-0.4277	0.0004	0.0282	0.0002					
$\kappa_{19,1}$	0.5076	0.0001	0.0209	0.0000					
$\kappa_{19,2}$	0.9926	0.0005	0.0279	0.0001					
$\kappa_{20,1}$	0.7472	0.0002	0.0299	0.0002					
$\kappa_{20,2}$	1.4469	0.0005	0.0390	0.0003					

Notes: $m(\hat{\psi})$ reports the arithmetic mean of the parameter estimates from the five different chains, $sd(\hat{\psi})$ denotes the corresponding standard deviation, $m(\hat{\sigma}_{\psi})$ denotes the arithmetic mean of the parameter specific standard deviation from the different chains, $sd(\hat{\sigma}_{\psi})$ the corresponding standard deviation.

Table 17: Simulation study (DART-m, Scenario 3) - Monte Carlo error of estimates

	$m(\hat{\psi})$	$sd(\hat{\psi})$	$m(\hat{\sigma}_{\psi})$	$sd(\hat{\sigma}_{\psi})$		$m(\hat{\psi})$	$sd(\hat{\psi})$	$m(\hat{\sigma}_{\psi})$	$sd(\hat{\sigma}_{\psi})$
$\gamma_{0,1}$	-0.6622	0.0531	0.2979	0.0117	ω_1	-0.4117	0.0502	0.3064	0.0125
$\gamma_{1,1}$	0.3835	0.0023	0.0471	0.0003	ω_2	-0.8786	0.0500	0.3057	0.0115
$\gamma_{2,1}$	0.1960	0.0015	0.0487	0.0006	ω_3	-1.7159	0.0476	0.3069	0.0117
$\gamma_{0,2}$	1.1591	0.0387	0.2180	0.0220	ω_4	0.1658	0.0503	0.3022	0.0121
$\gamma_{1,2}$	0.1398	0.0045	0.0379	0.0001	ω_5	-0.1135	0.0501	0.3021	0.0119
$\gamma_{2,2}$	-0.2186	0.0014	0.0408	0.0007	ω_6	0.5716	0.0523	0.3017	0.0116
σ_1^2	0.6855	0.0008	0.0307	0.0002	ω_7	-0.3152	0.0531	0.3023	0.0121
σ_2^2	0.3368	0.0007	0.0169	0.0001	ω_8	1.6511	0.0507	0.3027	0.0116
υ_1^2	0.6838	0.0039	0.3338	0.0116	ω_9	0.6883	0.0501	0.3033	0.0111
υ_2^2	0.5107	0.0096	0.2366	0.0113	ω_{10}	0.3274	0.0518	0.3043	0.0113
α_1	1.0332	0.0008	0.0312	0.0003	ω_{11}	-0.8565	0.0398	0.2224	0.0217
α_2	0.9935	0.0006	0.0299	0.0003	ω_{12}	-0.4167	0.0403	0.2214	0.0210
α_3	1.3328	0.0031	0.0425	0.0007	ω_{13}	-1.1427	0.0395	0.2202	0.0218
α_4	1.0711	0.0010	0.0332	0.0006	ω_{14}	-0.5063	0.0397	0.2197	0.0212
α_5	0.8649	0.0007	0.0259	0.0001	ω_{15}	0.1237	0.0390	0.2191	0.0216
α_6	1.0070	0.0010	0.0305	0.0007	ω_{16}	0.7786	0.0404	0.2202	0.0207
α_7	0.7445	0.0003	0.0228	0.0002	ω_{17}	-0.2174	0.0402	0.2198	0.0211
α_8	1.0676	0.0008	0.0341	0.0006	ω_{18}	0.9330	0.0395	0.2222	0.0201
α_9	0.7887	0.0003	0.0239	0.0003	ω_{19}	0.9934	0.0397	0.2242	0.0220
α_{10}	1.1317	0.0005	0.0342	0.0002	ω_{20}	0.6004	0.0416	0.2238	0.0214
α_{11}	1.1496	0.0009	0.0349	0.0007	$\gamma_{0,1}+\omega_1$	-1.0733	0.0025	0.1060	0.0008
α_{12}	0.7863	0.0008	0.0248	0.0003	$\gamma_{0,1}+\omega_2$	-1.5421	0.0037	0.0985	0.0012
α_{13}	1.1616	0.0009	0.0363	0.0006	$\gamma_{0,1}+\omega_3$	-2.3777	0.0045	0.1048	0.0015
α_{14}	1.3956	0.0027	0.0466	0.0015	$\gamma_{0,1}+\omega_4$	-0.4969	0.0016	0.0797	0.0009
α_{15}	0.9532	0.0012	0.0282	0.0003	$\gamma_{0,1}+\omega_5$	-0.7764	0.0019	0.0760	0.0004
α_{16}	0.7828	0.0003	0.0240	0.0002	$\gamma_{0,1}+\omega_6$	-0.0886	0.0021	0.0686	0.0003
α_{17}	0.8288	0.0007	0.0275	0.0005	$\gamma_{0,1}+\omega_7$	-0.9775	0.0013	0.0684	0.0003
α_{18}	1.0975	0.0009	0.0332	0.0003	$\gamma_{0,1}+\omega_8$	0.9905	0.0004	0.0676	0.0003
α_{19}	0.8674	0.0006	0.0223	0.0002	$\gamma_{0,1}+\omega_9$	0.0277	0.0010	0.0646	0.0006
α_{20}	1.2838	0.0004	0.0305	0.0003	$\gamma_{0,1}+\omega_{10}$	-0.3334	0.0007	0.0668	0.0003
β_1	-0.0509	0.0002	0.0259	0.0003	$\gamma_{0,2}+\omega_{11}$	0.3023	0.0031	0.0543	0.0002
β_2	-0.0604	0.0002	0.0255	0.0001	$\gamma_{0,2}+\omega_{12}$	0.7415	0.0036	0.0541	0.0003
β_3	-0.2181	0.0005	0.0283	0.0003	$\gamma_{0,2}+\omega_{13}$	0.0162	0.0019	0.0508	0.0003
β_4	-0.4088	0.0007	0.0268	0.0001	$\gamma_{0,2}+\omega_{14}$	0.6523	0.0031	0.0556	0.0005
β_5	-0.2302	0.0003	0.0246	0.0002	$\gamma_{0,2}+\omega_{15}$	1.2817	0.0029	0.0582	0.0002
β_6	-0.4864	0.0004	0.0267	0.0002	$\gamma_{0,2}+\omega_{16}$	1.9383	0.0035	0.0672	0.0005
β_7	-0.3282	0.0004	0.0238	0.0001	$\gamma_{0,2}+\omega_{17}$	0.9416	0.0033	0.0650	0.0008
β_8	0.8458	0.0006	0.0341	0.0003	$\gamma_{0,2}+\omega_{18}$	2.0920	0.0043	0.0776	0.0013
β_9	-0.1617	0.0002	0.0238	0.0002	$\gamma_{0,2}+\omega_{19}$	2.1525	0.0038	0.0844	0.0007
β_{10}	0.0257	0.0003	0.0270	0.0002	$\gamma_{0,2}+\omega_{20}$	1.7590	0.0047	0.0871	0.0013
β_{11}	-0.2466	0.0004	0.0271	0.0002					
β_{12}	-0.6358	0.0002	0.0258	0.0001					
β_{13}	0.5825	0.0006	0.0315	0.0005					
β_{14}	0.8539	0.0017	0.0387	0.0010					
β_{15}	0.0604	0.0004	0.0255	0.0002					
β_{16}	-0.3771	0.0002	0.0243	0.0003					
β_{17}	0.8711	0.0006	0.0312	0.0003					
β_{18}	0.2921	0.0003	0.0280	0.0004					
β_{19}	0.0990	0.0006	0.0250	0.0001					
β_{20}	-0.4275	0.0004	0.0282	0.0003					
$\kappa_{19,1}$	0.5080	0.0003	0.0209	0.0002					
$\kappa_{19,2}$	0.9932	0.0005	0.0279	0.0002					
$\kappa_{20,1}$	0.7477	0.0005	0.0300	0.0002					
$\kappa_{20,2}$	1.4478	0.0003	0.0393	0.0003					

Notes: $m(\hat{\psi})$ reports the arithmetic mean of the parameter estimates from the five different chains, $sd(\hat{\psi})$ denotes the corresponding standard deviation, $m(\hat{\sigma}_{\psi})$ denotes the arithmetic mean of the parameter specific standard deviation from the different chains, $sd(\hat{\sigma}_{\psi})$ the corresponding standard deviation.

Table 18: Simulation study (DART-m, Scenario 4) - Monte Carlo error of estimates

	$m(\hat{\psi})$	$sd(\hat{\psi})$	$m(\hat{\sigma}_{\psi})$	$sd(\hat{\sigma}_{\psi})$		$m(\hat{\psi})$	$sd(\hat{\psi})$	$m(\hat{\sigma}_{\psi})$	$sd(\hat{\sigma}_{\psi})$
$\gamma_{0,1}$	-0.6781	0.0076	0.2730	0.0167	ω_1	-0.3972	0.0070	0.2802	0.0173
$\gamma_{1,1}$	0.3525	0.0025	0.0488	0.0002	ω_2	-0.8635	0.0088	0.2793	0.0165
$\gamma_{2,1}$	0.1852	0.0016	0.0496	0.0006	ω_3	-1.7013	0.0092	0.2803	0.0182
$\gamma_{0,2}$	1.1741	0.0537	0.2355	0.0160	ω_4	0.1907	0.0081	0.2763	0.0176
$\gamma_{1,2}$	0.1400	0.0019	0.0347	0.0003	ω_5	-0.1035	0.0074	0.2762	0.0173
$\gamma_{2,2}$	-0.1952	0.0010	0.0389	0.0001	ω_6	0.6024	0.0070	0.2761	0.0165
σ_1^2	0.6927	0.0006	0.0313	0.0002	ω_7	-0.2906	0.0077	0.2770	0.0171
σ_2^2	0.3375	0.0005	0.0171	0.0002	ω_8	1.6868	0.0063	0.2779	0.0174
υ_1^2	0.6854	0.0044	0.3254	0.0123	ω_9	0.7155	0.0076	0.2783	0.0163
υ_2^2	0.4985	0.0070	0.2387	0.0095	ω_{10}	0.3668	0.0078	0.2811	0.0161
α_1	1.0341	0.0005	0.0308	0.0003	ω_{11}	-0.8672	0.0535	0.2406	0.0158
α_2	0.9934	0.0009	0.0300	0.0003	ω_{12}	-0.4395	0.0552	0.2388	0.0153
α_3	1.3336	0.0006	0.0431	0.0011	ω_{13}	-1.1513	0.0542	0.2378	0.0160
α_4	1.0709	0.0005	0.0325	0.0004	ω_{14}	-0.5328	0.0541	0.2372	0.0155
α_5	0.8642	0.0007	0.0260	0.0002	ω_{15}	0.0864	0.0554	0.2373	0.0151
α_6	1.0067	0.0011	0.0305	0.0003	ω_{16}	0.7277	0.0562	0.2374	0.0157
α_7	0.7438	0.0006	0.0230	0.0003	ω_{17}	-0.2583	0.0562	0.2366	0.0148
α_8	1.0661	0.0012	0.0338	0.0006	ω_{18}	0.8712	0.0562	0.2379	0.0146
α_9	0.7903	0.0005	0.0238	0.0003	ω_{19}	0.9414	0.0560	0.2388	0.0141
α_{10}	1.1330	0.0011	0.0343	0.0003	ω_{20}	0.5307	0.0570	0.2388	0.0149
α_{11}	1.1483	0.0008	0.0350	0.0007	$\gamma_{0,1}+\omega_1$	-1.0788	0.0035	0.1069	0.0016
α_{12}	0.7861	0.0009	0.0247	0.0002	$\gamma_{0,1}+\omega_2$	-1.5451	0.0032	0.1001	0.0007
α_{13}	1.1610	0.0023	0.0358	0.0003	$\gamma_{0,1}+\omega_3$	-2.3844	0.0018	0.1063	0.0009
α_{14}	1.3989	0.0022	0.0462	0.0012	$\gamma_{0,1}+\omega_4$	-0.4901	0.0032	0.0799	0.0004
α_{15}	0.9528	0.0005	0.0285	0.0004	$\gamma_{0,1}+\omega_5$	-0.7839	0.0020	0.0766	0.0005
α_{16}	0.7825	0.0006	0.0241	0.0002	$\gamma_{0,1}+\omega_6$	-0.0769	0.0011	0.0692	0.0006
α_{17}	0.8284	0.0005	0.0275	0.0004	$\gamma_{0,1}+\omega_7$	-0.9702	0.0008	0.0687	0.0005
α_{18}	1.0998	0.0013	0.0331	0.0007	$\gamma_{0,1}+\omega_8$	1.0080	0.0017	0.0681	0.0005
α_{19}	0.8664	0.0007	0.0223	0.0002	$\gamma_{0,1}+\omega_9$	0.0375	0.0012	0.0646	0.0003
α_{20}	1.2836	0.0005	0.0305	0.0005	$\gamma_{0,1}+\omega_{10}$	-0.3114	0.0012	0.0665	0.0002
β_1	-0.0505	0.0005	0.0258	0.0002	$\gamma_{0,2}+\omega_{11}$	0.3076	0.0013	0.0530	0.0003
β_2	-0.0602	0.0007	0.0255	0.0001	$\gamma_{0,2}+\omega_{12}$	0.7361	0.0014	0.0525	0.0004
β_3	-0.2182	0.0003	0.0283	0.0001	$\gamma_{0,2}+\omega_{13}$	0.0231	0.0011	0.0498	0.0002
β_4	-0.4094	0.0005	0.0268	0.0002	$\gamma_{0,2}+\omega_{14}$	0.6416	0.0013	0.0539	0.0001
β_5	-0.2305	0.0002	0.0245	0.0001	$\gamma_{0,2}+\omega_{15}$	1.2618	0.0008	0.0568	0.0002
β_6	-0.4865	0.0005	0.0267	0.0002	$\gamma_{0,2}+\omega_{16}$	1.9015	0.0019	0.0653	0.0003
β_7	-0.3283	0.0002	0.0237	0.0003	$\gamma_{0,2}+\omega_{17}$	0.9168	0.0016	0.0641	0.0004
β_8	0.8446	0.0014	0.0339	0.0007	$\gamma_{0,2}+\omega_{18}$	2.0473	0.0030	0.0757	0.0006
β_9	-0.1614	0.0006	0.0239	0.0001	$\gamma_{0,2}+\omega_{19}$	2.1182	0.0020	0.0833	0.0005
β_{10}	0.0256	0.0003	0.0270	0.0002	$\gamma_{0,2}+\omega_{20}$	1.7070	0.0011	0.0848	0.0004
β_{11}	-0.2468	0.0005	0.0270	0.0002					
β_{12}	-0.6362	0.0004	0.0257	0.0002					
β_{13}	0.5828	0.0013	0.0314	0.0003					
β_{14}	0.8555	0.0015	0.0386	0.0007					
β_{15}	0.0606	0.0002	0.0254	0.0002					
β_{16}	-0.3772	0.0002	0.0242	0.0001					
β_{17}	0.8703	0.0007	0.0314	0.0004					
β_{18}	0.2929	0.0004	0.0282	0.0002					
β_{19}	0.0990	0.0004	0.0250	0.0002					
β_{20}	-0.4275	0.0005	0.0284	0.0002					
$\kappa_{19,1}$	0.5075	0.0002	0.0209	0.0001					
$\kappa_{19,2}$	0.9924	0.0003	0.0280	0.0001					
$\kappa_{20,1}$	0.7480	0.0003	0.0300	0.0002					
$\kappa_{20,2}$	1.4480	0.0006	0.0394	0.0003					

Notes: $m(\hat{\psi})$ reports the arithmetic mean of the parameter estimates from the five different chains, $sd(\hat{\psi})$ denotes the corresponding standard deviation, $m(\hat{\sigma}_{\psi})$ denotes the arithmetic mean of the parameter specific standard deviation from the different chains, $sd(\hat{\sigma}_{\psi})$ the corresponding standard deviation.

Table 19: NEPS GRADE 9, MATHEMATICAL COMPETENCIES (model I) – Convergence diagnostic (Geweke) of regression parameters

	HS	RS	GYM	$OTHER$
$\gamma_{g,Intercept}$	-1.455	-0.591	-0.231	-1.161
$\gamma_{g,Gender:1}$	-2.416	0.356	0.048	1.046
$\gamma_{g,HISEI}$	1.501	0.831	0.676	1.942
$\gamma_{g,Age}$	1.146	0.511	0.686	0.707
$\gamma_{g,Experience}$	0.219	-0.267	-0.450	0.749
$\gamma_{g,HISEI \times Age}$	-1.259	-1.185	-0.733	-1.733
$\gamma_{g,HISEI \times Experience}$	-0.061	1.014	0.255	0.142
σ_g^2	-1.295	0.249	2.147	1.226
υ_g^2	2.449	0.343	-0.972	-0.341

Notes: $C = 532$; $N = 14320$; $N_{CC} = 6748$; $J = 22$. Geweke statistic is calculated using the first MCMC trajectory with 20000 iterations after a burn in of 5000, assessing equality of means for the first 20% and last 50% of the MCMC trajectory. For model I less then 5% (4 out of 84) of the statistics are above the 95% critical value of $|\pm 1.96|$.

Table 20: NEPS grade 9, mathematical competencies (model I) – Convergence diagnostic (Geweke) of item parameters

Item discrimination		Item difficulty		Item category cutoff	
α_1	-1.117	β_1	-0.404	$\kappa_{3,2}$	-0.075
α_2	-0.306	β_2	0.778	$\kappa_{3,3}$	-0.914
α_3	0.195	β_3	0.224	$\kappa_{16,2}$	-1.281
α_4	0.914	β_4	1.358	$\kappa_{16,3}$	-1.364
α_5	-0.056	β_5	-0.736		
α_6	0.669	β_6	-0.342		
α_7	0.982	β_7	-1.313		
α_8	1.428	β_8	-0.789		
α_9	0.973	β_9	-0.948		
α_{10}	-0.594	β_{10}	-0.610		
α_{11}	-0.282	β_{11}	0.020		
α_{12}	-0.099	β_{12}	-0.187		
α_{13}	0.792	β_{13}	0.303		
α_{14}	-0.386	β_{14}	-1.066		
α_{15}	0.493	β_{15}	0.919		
α_{16}	-1.476	β_{16}	1.024		
α_{17}	-0.147	β_{17}	-0.965		
α_{18}	-0.536	β_{18}	-0.201		
α_{19}	-1.375	β_{19}	0.685		
α_{20}	0.365	β_{20}	-0.901		
α_{21}	-0.269	β_{21}	0.491		
α_{22}	-2.675	β_{22}	-1.953		

Notes: $C = 532$; $N = 14320$; $N_{CC} = 6748$; $J = 22$. Geweke statistic is calculated using the first MCMC trajectory with 20000 iterations after a burn in of 5000, assessing equality of means for the first 20% and last 50% of the MCMC trajectory. For model I less then 5% (4 out of 84) of the statistics are above the 95% critical value of $|\pm 1.96|$.

Table 21: NEPS GRADE 9, MATHEMATICAL COMPETENCIES (model II) – Convergence diagnostic (Geweke) of regression parameters

	HS	RS	GYM	$OTHER$
$\gamma_{g,Intercept}$	-0.056	0.746	0.212	0.122
$\gamma_{g,Gender:1}$	-0.677	-2.295	1.211	0.347
$\gamma_{g,GenerationStatus:1}$	0.537	-0.994	-0.329	0.394
$\gamma_{g,GenerationStatus:2}$	0.594	-0.736	-0.224	-1.609
$\gamma_{g,GenerationStatus:3}$	-0.289	0.354	0.198	-0.797
$\gamma_{g,GradeMathematics:2}$	0.151	1.610	-0.037	-0.025
$\gamma_{g,GradeMathematics:3}$	-0.159	2.339	0.936	-0.655
$\gamma_{g,GradeMathematics:4}$	-0.717	1.891	0.677	-0.342
$\gamma_{g,GradeMathematics:5}$	-0.578	1.609	-0.807	-0.580
$\gamma_{g,GradeMathematics:6}$	-0.584	-1.138	-1.173	0.119
$\gamma_{g,SchoolYearRepeated:1}$	0.213	-0.644	-1.243	-1.101
$\gamma_{g,Computer:2}$	-0.913	-0.013	0.119	-0.427
$\gamma_{g,Computer:3}$	1.079	0.983	0.377	-1.777
$\gamma_{g,Room:1}$	-0.476	-1.011	2.030	-0.551
$\gamma_{g,HCASMIN:1}$	0.857	-0.886	-0.593	-1.230
$\gamma_{g,HCASMIN:2}$	0.427	-0.637	-0.892	0.428
$\gamma_{g,HCASMIN:3}$	-0.021	-0.413	-0.195	0.265
$\gamma_{g,HCASMIN:4}$	1.364	-0.258	-0.451	0.065
$\gamma_{g,HCASMIN:5}$	1.699	-0.335	-0.509	0.375
$\gamma_{g,HCASMIN:6}$	-0.348	-0.458	-0.269	0.278
$\gamma_{g,HCASMIN:7}$	1.620	-0.181	-0.337	0.602
$\gamma_{g,HCASMIN:8}$	0.239	-0.403	-0.629	0.087
σ_g^2	0.610	-0.768	-2.149	1.782
υ_g^2	-0.519	-1.090	0.284	-1.482

Notes: $C = 532$; $N = 14320$; $N_{CC} = 7708$; $J = 22$. Geweke statistic is calculated using the first MCMC trajectory with 20000 iterations after a burn in of 5000, assessing equality of means for the first 20% and last 50% of the MCMC trajectory. For model II less then 5% (5 out of 144) of the statistics are above the 95% critical value of $|\pm 1.96|$.

Table 22: NEPS GRADE 9, MATHEMATICAL COMPETENCIES (model II) – Convergence diagnostic (Geweke) of item parameters

Item discrimination		Item difficulty		Item category cutoff	
α_1	-0.895	β_1	-1.493	$\kappa_{3,2}$	0.576
α_2	1.047	β_2	1.053	$\kappa_{3,3}$	0.617
α_3	1.455	β_3	-0.455	$\kappa_{16,2}$	0.506
α_4	-0.185	β_4	-0.633	$\kappa_{16,3}$	0.342
α_5	-1.810	β_5	1.340		
α_6	0.134	β_6	0.798		
α_7	0.357	β_7	-0.134		
α_8	0.272	β_8	0.774		
α_9	-0.599	β_9	-0.981		
α_{10}	-0.554	β_{10}	0.079		
α_{11}	0.100	β_{11}	-0.042		
α_{12}	0.434	β_{12}	-0.312		
α_{13}	-1.082	β_{13}	-0.895		
α_{14}	1.893	β_{14}	2.143		
α_{15}	-0.482	β_{15}	-0.434		
α_{16}	0.323	β_{16}	-0.059		
α_{17}	1.263	β_{17}	0.624		
α_{18}	-1.699	β_{18}	-0.901		
α_{19}	-0.012	β_{19}	1.101		
α_{20}	0.170	β_{20}	0.445		
α_{21}	1.307	β_{21}	-0.064		
α_{22}	-0.577	β_{22}	-0.627		

Notes: $C = 532$; $N = 14320$; $N_{CC} = 7708$; $J = 22$. Geweke statistic is calculated using the first MCMC trajectory with 20000 iterations after a burn in of 5000, assessing equality of means for the first 20% and last 50% of the MCMC trajectory. For model II less then 5% (5 out of 144) of the statistics are above the 95% critical value of $|\pm 1.96|$.

Table 23: NEPS GRADE 9, MATHEMATICAL COMPETENCIES (model I) – Convergence diagnostic (Gelman-Rubin $\hat{R}$ and ESS)

ψ	$\widehat{R}$ (UCB)	ESS	ψ	$\widehat{R}$ (UCB)	ESS
$\gamma_{0,1}$	1.00 (1.00)	24243	α_1	1.00 (1.00)	16748
$\gamma_{1,1}$	1.00 (1.00)	27102	α_2	1.00 (1.00)	19073
$\gamma_{2,1}$	1.00 (1.01)	24127	α_3	1.00 (1.00)	21064
$\gamma_{3,1}$	1.00 (1.00)	27647	α_4	1.00 (1.00)	7207
$\gamma_{4,1}$	1.00 (1.00)	33453	α_5	1.00 (1.00)	13884
$\gamma_{5,1}$	1.00 (1.00)	28181	α_6	1.00 (1.00)	23525
$\gamma_{6,1}$	1.00 (1.00)	36399	α_7	1.00 (1.01)	6759
$\gamma_{0,2}$	1.00 (1.00)	31189	α_8	1.00 (1.00)	22104
$\gamma_{1,2}$	1.00 (1.00)	33502	α_9	1.00 (1.00)	10591
$\gamma_{2,2}$	1.00 (1.00)	31664	α_{10}	1.00 (1.00)	15136
$\gamma_{3,2}$	1.00 (1.00)	35420	α_{11}	1.00 (1.00)	24666
$\gamma_{4,2}$	1.00 (1.00)	42932	α_{12}	1.00 (1.00)	19826
$\gamma_{5,2}$	1.00 (1.00)	35612	α_{13}	1.00 (1.00)	17856
$\gamma_{6,2}$	1.00 (1.00)	42995	α_{14}	1.00 (1.00)	19537
$\gamma_{0,3}$	1.00 (1.00)	37170	α_{15}	1.00 (1.00)	6261
$\gamma_{1,3}$	1.00 (1.00)	32317	α_{16}	1.00 (1.00)	25594
$\gamma_{2,3}$	1.00 (1.00)	35814	α_{17}	1.00 (1.00)	12940
$\gamma_{3,3}$	1.00 (1.00)	39889	α_{18}	1.00 (1.00)	20262
$\gamma_{4,3}$	1.00 (1.00)	46650	α_{19}	1.00 (1.00)	8114
$\gamma_{5,3}$	1.00 (1.00)	38639	α_{20}	1.00 (1.00)	16428
$\gamma_{6,3}$	1.00 (1.00)	45267	α_{21}	1.00 (1.00)	11206
$\gamma_{0,4}$	1.00 (1.00)	36351	α_{22}	1.00 (1.00)	16334
$\gamma_{1,4}$	1.00 (1.00)	36310	β_1	1.00 (1.00)	35538
$\gamma_{2,4}$	1.00 (1.00)	36634	β_2	1.00 (1.00)	36268
$\gamma_{3,4}$	1.00 (1.00)	39585	β_3	1.00 (1.00)	15956
$\gamma_{4,4}$	1.00 (1.00)	47822	β_4	1.00 (1.00)	6286
$\gamma_{5,4}$	1.00 (1.00)	38783	β_5	1.00 (1.00)	34194
$\gamma_{6,4}$	1.00 (1.00)	47366	β_6	1.00 (1.00)	40173
σ_1^2	1.00 (1.00)	11122	β_7	1.00 (1.00)	15418
σ_2^2	1.00 (1.00)	14574	β_8	1.00 (1.00)	34924
σ_3^2	1.00 (1.00)	12473	β_9	1.00 (1.00)	28551
σ_4^2	1.00 (1.00)	16394	β_{10}	1.00 (1.00)	33230
υ_1^2	1.00 (1.00)	36985	β_{11}	1.00 (1.00)	31105
υ_2^2	1.00 (1.00)	46063	β_{12}	1.00 (1.00)	33323
υ_3^2	1.00 (1.00)	54312	β_{13}	1.00 (1.00)	39148
υ_4^2	1.00 (1.00)	49016	β_{14}	1.00 (1.00)	22586
			β_{15}	1.00 (1.00)	11440
			β_{16}	1.00 (1.00)	14317
			β_{17}	1.00 (1.00)	16799
			β_{18}	1.00 (1.00)	33620
			β_{19}	1.00 (1.00)	26272
			β_{20}	1.00 (1.00)	30740
			β_{21}	1.00 (1.00)	29954
			β_{22}	1.00 (1.00)	32575
			$\kappa_{3,2}$	1.00 (1.00)	22713
			$\kappa_{3,3}$	1.00 (1.00)	15398
			$\kappa_{16,2}$	1.00 (1.00)	15430
			$\kappa_{16,3}$	1.00 (1.00)	14172
$\widehat{R}_M$				1.01	

Notes: $C = 532$; $N = 14320$; $N_{CC} = 6748$; $J = 22$. Gelman-Rubin statistic ($\hat{R}$) and effective sample size (ESS) are calculated using the first 20000 iterations after a burn in of 5000 for each of the five MCMC trajectories. The upper confidence bound is provided in parentheses (UCB).

Table 24: NEPS GRADE 9, MATHEMATICAL COMPETENCIES (model II) – Convergence diagnostic (Gelman Rubin $\hat{R}$ and ESS)

ψ	$\widehat{R}$ (UCB)	ESS	ψ	$\widehat{R}$ (UCB)	ESS	ψ	$\widehat{R}$ (UCB)	ESS
$\gamma_{0,1}$	1.00 (1.00)	19089	$\gamma_{0,3}$	1.00 (1.00)	29146	α_1	1.00 (1.00)	18042
$\gamma_{1,1}$	1.00 (1.00)	24062	$\gamma_{1,3}$	1.00 (1.00)	27427	α_2	1.00 (1.00)	20085
$\gamma_{2,1}$	1.00 (1.01)	24800	$\gamma_{2,3}$	1.00 (1.00)	31143	α_3	1.00 (1.00)	21203
$\gamma_{3,1}$	1.00 (1.00)	23356	$\gamma_{3,3}$	1.00 (1.00)	29376	α_4	1.00 (1.00)	7348
$\gamma_{4,1}$	1.00 (1.00)	25114	$\gamma_{4,3}$	1.00 (1.00)	30053	α_5	1.00 (1.00)	13677
$\gamma_{5,1}$	1.00 (1.00)	26851	$\gamma_{5,3}$	1.00 (1.00)	18989	α_6	1.00 (1.00)	25537
$\gamma_{6,1}$	1.00 (1.00)	25763	$\gamma_{6,3}$	1.00 (1.00)	18562	α_7	1.00 (1.01)	7222
$\gamma_{7,1}$	1.00 (1.00)	25742	$\gamma_{7,3}$	1.00 (1.00)	19750	α_8	1.00 (1.00)	23220
$\gamma_{8,1}$	1.00 (1.00)	24817	$\gamma_{8,3}$	1.00 (1.00)	25875	α_9	1.00 (1.00)	11118
$\gamma_{9,1}$	1.00 (1.00)	25121	$\gamma_{9,3}$	1.00 (1.00)	31768	α_{10}	1.00 (1.00)	15608
$\gamma_{10,1}$	1.00 (1.00)	23365	$\gamma_{10,3}$	1.00 (1.00)	32055	α_{11}	1.00 (1.00)	24193
$\gamma_{11,1}$	1.00 (1.00)	26929	$\gamma_{11,3}$	1.00 (1.00)	29298	α_{12}	1.00 (1.00)	19421
$\gamma_{12,1}$	1.00 (1.00)	24823	$\gamma_{12,3}$	1.00 (1.00)	30228	α_{13}	1.00 (1.00)	19015
$\gamma_{13,1}$	1.00 (1.00)	24285	$\gamma_{13,3}$	1.00 (1.00)	29894	α_{14}	1.00 (1.00)	19945
$\gamma_{14,1}$	1.00 (1.00)	23842	$\gamma_{14,3}$	1.00 (1.00)	42422	α_{15}	1.00 (1.00)	6509
$\gamma_{15,1}$	1.00 (1.00)	23678	$\gamma_{15,3}$	1.00 (1.00)	42539	α_{16}	1.00 (1.00)	28098
$\gamma_{16,1}$	1.00 (1.00)	23303	$\gamma_{16,3}$	1.00 (1.00)	42714	α_{17}	1.00 (1.00)	13102
$\gamma_{17,1}$	1.00 (1.00)	23643	$\gamma_{17,3}$	1.00 (1.00)	42835	α_{18}	1.00 (1.00)	21171
$\gamma_{18,1}$	1.00 (1.00)	23430	$\gamma_{18,3}$	1.00 (1.00)	42521	α_{19}	1.00 (1.00)	8040
$\gamma_{19,1}$	1.00 (1.00)	23759	$\gamma_{19,3}$	1.00 (1.00)	42269	α_{20}	1.00 (1.00)	16096
$\gamma_{20,1}$	1.00 (1.00)	24207	$\gamma_{20,3}$	1.00 (1.00)	41789	α_{21}	1.00 (1.00)	11031
$\gamma_{21,1}$	1.00 (1.00)	24405	$\gamma_{21,3}$	1.00 (1.00)	41897	α_{22}	1.00 (1.00)	17331
$\gamma_{0,2}$	1.00 (1.00)	25881	$\gamma_{0,4}$	1.00 (1.00)	13910	β_1	1.00 (1.00)	39265
$\gamma_{1,2}$	1.00 (1.00)	27028	$\gamma_{1,4}$	1.00 (1.00)	31756	β_2	1.00 (1.00)	37722
$\gamma_{2,2}$	1.00 (1.00)	29648	$\gamma_{2,4}$	1.00 (1.00)	30043	β_3	1.00 (1.00)	16229
$\gamma_{3,2}$	1.00 (1.00)	27598	$\gamma_{3,4}$	1.00 (1.00)	30062	β_4	1.00 (1.00)	6486
$\gamma_{4,2}$	1.00 (1.00)	29381	$\gamma_{4,4}$	1.00 (1.00)	31588	β_5	1.00 (1.00)	34011
$\gamma_{5,2}$	1.00 (1.00)	27356	$\gamma_{5,4}$	1.00 (1.00)	31869	β_6	1.00 (1.00)	41041
$\gamma_{6,2}$	1.00 (1.00)	26272	$\gamma_{6,4}$	1.00 (1.00)	30894	β_7	1.00 (1.00)	16088
$\gamma_{7,2}$	1.00 (1.00)	26126	$\gamma_{7,4}$	1.00 (1.00)	30557	β_8	1.00 (1.00)	33220
$\gamma_{8,2}$	1.00 (1.00)	26958	$\gamma_{8,4}$	1.00 (1.00)	30928	β_9	1.00 (1.00)	29086
$\gamma_{9,2}$	1.00 (1.00)	31644	$\gamma_{9,4}$	1.00 (1.00)	29852	β_{10}	1.00 (1.00)	33936
$\gamma_{10,2}$	1.00 (1.00)	27448	$\gamma_{10,4}$	1.00 (1.00)	29132	β_{11}	1.00 (1.00)	30620
$\gamma_{11,2}$	1.00 (1.00)	29426	$\gamma_{11,4}$	1.00 (1.00)	31754	β_{12}	1.00 (1.00)	33412
$\gamma_{12,2}$	1.00 (1.00)	29919	$\gamma_{12,4}$	1.00 (1.00)	31853	β_{13}	1.00 (1.00)	38429
$\gamma_{13,2}$	1.00 (1.00)	29496	$\gamma_{13,4}$	1.00 (1.00)	30853	β_{14}	1.00 (1.00)	22680
$\gamma_{14,2}$	1.00 (1.00)	31693	$\gamma_{14,4}$	1.00 (1.00)	37008	β_{15}	1.00 (1.00)	11582
$\gamma_{15,2}$	1.00 (1.00)	32018	$\gamma_{15,4}$	1.00 (1.00)	36039	β_{16}	1.00 (1.00)	14472
$\gamma_{16,2}$	1.00 (1.00)	31380	$\gamma_{16,4}$	1.00 (1.00)	36590	β_{17}	1.00 (1.00)	16414
$\gamma_{17,2}$	1.00 (1.00)	31613	$\gamma_{17,4}$	1.00 (1.00)	35812	β_{18}	1.00 (1.00)	34063
$\gamma_{18,2}$	1.00 (1.00)	30120	$\gamma_{18,4}$	1.00 (1.00)	36905	β_{19}	1.00 (1.00)	25707
$\gamma_{19,2}$	1.00 (1.00)	31559	$\gamma_{19,4}$	1.00 (1.00)	35685	β_{20}	1.00 (1.00)	31859
$\gamma_{20,2}$	1.00 (1.00)	31252	$\gamma_{20,4}$	1.00 (1.00)	35415	β_{21}	1.00 (1.00)	32225
$\gamma_{21,2}$	1.00 (1.00)	30963	$\gamma_{21,4}$	1.00 (1.00)	35449	β_{22}	1.00 (1.00)	33933
σ_1^2	1.00 (1.00)	10389	υ_1^2	1.00 (1.00)	37712	$\kappa_{3,2}$	1.00 (1.00)	22083
σ_2^2	1.00 (1.00)	12533	υ_2^2	1.00 (1.00)	45337	$\kappa_{3,3}$	1.00 (1.00)	15454
σ_3^2	1.00 (1.00)	9912	υ_3^2	1.00 (1.00)	51353	$\kappa_{16,2}$	1.00 (1.00)	15099
σ_4^2	1.00 (1.00)	13926	υ_4^2	1.00 (1.00)	48470	$\kappa_{16,3}$	1.00 (1.00)	14555
$\widehat{R}_M$				1.01				

Notes: $C = 532$; $N = 14320$; $N_{CC} = 7708$; $J = 22$. Gelman-Rubin statistic ($\hat{R}$) and effective sample size (ESS) are calculated using the first 20000 iterations after a burn in of 5000 for each of the five MCMC trajectories. The upper confidence bound is provided in parentheses (UCB).

Table 25: NEPS GRADE 9, MATHEMATICAL COMPETENCIES (model I) – Monte Carlo error of estimates

	$m(\hat{\psi})$	$sd(\hat{\psi})$	$m(\hat{\sigma}_\psi)$	$sd(\hat{\sigma}_\psi)$		$m(\hat{\psi})$	$sd(\hat{\psi})$	$m(\hat{\sigma}_\psi)$	$sd(\hat{\sigma}_\psi)$
$\gamma_{0,1}$	0.8552	0.0156	0.4388	0.0020	α_1	0.9326	0.0005	0.0227	0.0001
$\gamma_{1,1}$	-0.2151	0.0003	0.0148	0.0001	α_2	0.9714	0.0006	0.0217	0.0002
$\gamma_{2,1}$	-0.0227	0.0036	0.1011	0.0010	α_3	0.9393	0.0001	0.0192	0.0001
$\gamma_{3,1}$	-0.0547	0.0014	0.0382	0.0002	α_4	1.0469	0.0009	0.0294	0.0004
$\gamma_{4,1}$	-0.0304	0.0012	0.0354	0.0001	α_5	1.0326	0.0003	0.0244	0.0001
$\gamma_{5,1}$	-0.0024	0.0003	0.0087	0.0001	α_6	0.6978	0.0002	0.0196	0.0001
$\gamma_{6,1}$	0.0078	0.0003	0.0080	0.0000	α_7	1.3517	0.0012	0.0325	0.0004
$\gamma_{0,2}$	1.8772	0.0252	0.5270	0.0009	α_8	0.8296	0.0005	0.0203	0.0002
$\gamma_{1,2}$	-0.3128	0.0002	0.0148	0.0001	α_9	1.2463	0.0007	0.0276	0.0003
$\gamma_{2,2}$	-0.1164	0.0056	0.1047	0.0004	α_{10}	1.1625	0.0005	0.0243	0.0002
$\gamma_{3,2}$	-0.1094	0.0013	0.0457	0.0001	α_{11}	0.6492	0.0002	0.0195	0.0001
$\gamma_{4,2}$	-0.0127	0.0015	0.0456	0.0002	α_{12}	1.0114	0.0003	0.0221	0.0001
$\gamma_{5,2}$	0.0061	0.0003	0.0090	0.0000	α_{13}	0.7859	0.0006	0.0218	0.0002
$\gamma_{6,2}$	0.0044	0.0003	0.0090	0.0000	α_{14}	0.8262	0.0004	0.0211	0.0001
$\gamma_{0,3}$	1.8593	0.0125	0.7507	0.0067	α_{15}	1.0205	0.0010	0.0320	0.0002
$\gamma_{1,3}$	-0.3125	0.0001	0.0164	0.0000	α_{16}	0.7350	0.0002	0.0179	0.0001
$\gamma_{2,3}$	0.2080	0.0023	0.1180	0.0010	α_{17}	1.2089	0.0003	0.0273	0.0001
$\gamma_{3,3}$	-0.0714	0.0007	0.0595	0.0002	α_{18}	0.9366	0.0003	0.0215	0.0001
$\gamma_{4,3}$	-0.0182	0.0003	0.0603	0.0004	α_{19}	1.5017	0.0004	0.0316	0.0006
$\gamma_{5,3}$	-0.0100	0.0001	0.0094	0.0000	α_{20}	1.1475	0.0003	0.0239	0.0002
$\gamma_{6,3}$	-0.0034	0.0001	0.0096	0.0000	α_{21}	1.3834	0.0008	0.0284	0.0003
$\gamma_{0,4}$	1.0092	0.0526	1.1707	0.0024	α_{22}	1.1147	0.0004	0.0239	0.0003
$\gamma_{1,4}$	-0.2681	0.0004	0.0334	0.0002	β_1	-0.2103	0.0002	0.0112	0.0001
$\gamma_{2,4}$	0.1252	0.0094	0.2225	0.0004	β_2	0.1491	0.0002	0.0114	0.0001
$\gamma_{3,4}$	-0.0583	0.0037	0.0945	0.0003	β_3	-1.2397	0.0002	0.0152	0.0001
$\gamma_{4,4}$	-0.0249	0.0013	0.0933	0.0006	β_4	1.6937	0.0008	0.0229	0.0002
$\gamma_{5,4}$	-0.0078	0.0007	0.0178	0.0001	β_5	-0.2971	0.0003	0.0115	0.0001
$\gamma_{6,4}$	0.0024	0.0002	0.0175	0.0001	β_6	0.1324	0.0001	0.0112	0.0000
σ_1^2	0.1043	0.0001	0.0046	0.0000	β_7	-0.7111	0.0002	0.0132	0.0001
σ_2^2	0.1383	0.0001	0.0051	0.0001	β_8	0.3830	0.0001	0.0116	0.0001
σ_3^2	0.2259	0.0001	0.0068	0.0000	β_9	-0.3849	0.0002	0.0119	0.0001
σ_4^2	0.1529	0.0003	0.0118	0.0001	β_{10}	0.0780	0.0001	0.0118	0.0000
υ_1^2	0.0476	0.0000	0.0059	0.0000	β_{11}	0.6385	0.0002	0.0123	0.0000
υ_2^2	0.0598	0.0001	0.0079	0.0001	β_{12}	0.2901	0.0002	0.0117	0.0002
υ_3^2	0.0909	0.0001	0.0114	0.0001	β_{13}	-0.3458	0.0002	0.0112	0.0001
υ_4^2	0.0933	0.0003	0.0248	0.0002	β_{14}	0.8634	0.0001	0.0134	0.0001
					β_{15}	-1.1026	0.0002	0.0146	0.0000
					β_{16}	-1.4037	0.0003	0.0162	0.0001
					β_{17}	0.8778	0.0004	0.0168	0.0001
					β_{18}	0.3323	0.0001	0.0118	0.0001
					β_{19}	-0.2801	0.0001	0.0124	0.0001
					β_{20}	0.3179	0.0001	0.0123	0.0001
					β_{21}	-0.0018	0.0001	0.0121	0.0001
					β_{22}	0.2201	0.0002	0.0120	0.0001
					$\kappa_{3,2}$	0.6316	0.0002	0.0134	0.0000
					$\kappa_{3,3}$	1.8162	0.0003	0.0185	0.0001
					$\kappa_{16,2}$	1.2445	0.0004	0.0170	0.0001
					$\kappa_{16,3}$	1.8543	0.0003	0.0188	0.0002

Notes: $m(\hat{\psi})$ reports the arithmetic mean of the parameter estimates from the five different chains, $sd(\hat{\psi})$ denotes the corresponding standard deviation, $m(\hat{\sigma}_\psi)$ denotes the arithmetic mean of the parameter specific standard deviation from the different chains, $sd(\hat{\sigma}_\psi)$ the corresponding standard deviation.

Table 26: NEPS grade 9, mathematical competencies (model II) – Monte Carlo error of estimates

	$m(\hat{\psi})$	$sd(\hat{\psi})$	$m(\hat{\sigma}_\psi)$	$sd(\hat{\sigma}_\psi)$		$m(\hat{\psi})$	$sd(\hat{\psi})$	$m(\hat{\sigma}_\psi)$	$sd(\hat{\sigma}_\psi)$		$m(\hat{\psi})$	$sd(\hat{\psi})$	$m(\hat{\sigma}_\psi)$	$sd(\hat{\sigma}_\psi)$
$\gamma_{0,1}$	-0.1070	0.0015	0.0666	0.0005	$\gamma_{0,3}$	1.3443	0.0019	0.1305	0.0007	α_1	0.9297	0.0004	0.0224	0.0002
$\gamma_{1,1}$	-0.1605	0.0001	0.0145	0.0001	$\gamma_{1,3}$	-0.2943	0.0002	0.0143	0.0001	α_2	0.9633	0.0006	0.0215	0.0002
$\gamma_{2,1}$	-0.0662	0.0005	0.0279	0.0003	$\gamma_{2,3}$	-0.2511	0.0004	0.0419	0.0004	α_3	0.9503	0.0003	0.0190	0.0003
$\gamma_{3,1}$	-0.0892	0.0005	0.0221	0.0001	$\gamma_{3,3}$	-0.1574	0.0005	0.0304	0.0002	α_4	1.0523	0.0005	0.0287	0.0007
$\gamma_{4,1}$	0.0120	0.0006	0.0257	0.0001	$\gamma_{4,3}$	-0.0593	0.0004	0.0231	0.0001	α_5	1.0417	0.0006	0.0242	0.0003
$\gamma_{5,1}$	-0.1089	0.0012	0.0373	0.0002	$\gamma_{5,3}$	-0.4462	0.0007	0.0282	0.0003	α_6	0.7057	0.0002	0.0195	0.0002
$\gamma_{6,1}$	-0.3008	0.0009	0.0367	0.0001	$\gamma_{6,3}$	-0.7670	0.0006	0.0277	0.0002	α_7	1.3361	0.0006	0.0318	0.0005
$\gamma_{7,1}$	-0.4219	0.0007	0.0377	0.0002	$\gamma_{7,3}$	-0.9503	0.0006	0.0293	0.0002	α_8	0.8291	0.0002	0.0202	0.0002
$\gamma_{8,1}$	-0.4289	0.0006	0.0425	0.0002	$\gamma_{8,3}$	-1.0183	0.0005	0.0430	0.0004	α_9	1.2365	0.0006	0.0272	0.0005
$\gamma_{9,1}$	-0.4597	0.0013	0.1059	0.0006	$\gamma_{9,3}$	-0.8714	0.0041	0.1937	0.0015	α_{10}	1.1510	0.0002	0.0241	0.0002
$\gamma_{10,1}$	0.0320	0.0009	0.0155	0.0001	$\gamma_{10,3}$	0.0829	0.0007	0.0263	0.0002	α_{11}	0.6512	0.0002	0.0192	0.0001
$\gamma_{11,1}$	0.0041	0.0004	0.0158	0.0001	$\gamma_{11,3}$	0.0232	0.0003	0.0165	0.0002	α_{12}	1.0047	0.0003	0.0218	0.0002
$\gamma_{12,1}$	-0.0298	0.0018	0.0614	0.0005	$\gamma_{12,3}$	-0.0307	0.0009	0.1181	0.0005	α_{13}	0.7882	0.0002	0.0216	0.0002
$\gamma_{13,1}$	0.0243	0.0003	0.0233	0.0001	$\gamma_{13,3}$	0.0175	0.0006	0.0378	0.0002	α_{14}	0.8346	0.0003	0.0208	0.0002
$\gamma_{14,1}$	0.1161	0.0008	0.0538	0.0005	$\gamma_{14,3}$	-0.0221	0.0008	0.1367	0.0004	α_{15}	1.0218	0.0008	0.0317	0.0005
$\gamma_{15,1}$	0.1130	0.0005	0.0518	0.0004	$\gamma_{15,3}$	0.0279	0.0015	0.1269	0.0007	α_{16}	0.7434	0.0004	0.0178	0.0001
$\gamma_{16,1}$	0.1129	0.0009	0.0563	0.0004	$\gamma_{16,3}$	-0.0036	0.0015	0.1305	0.0008	α_{17}	1.2155	0.0004	0.0271	0.0002
$\gamma_{17,1}$	0.1328	0.0004	0.0522	0.0005	$\gamma_{17,3}$	0.0077	0.0019	0.1223	0.0008	α_{18}	0.9344	0.0004	0.0212	0.0003
$\gamma_{18,1}$	0.1320	0.0005	0.0586	0.0004	$\gamma_{18,3}$	0.0494	0.0018	0.1243	0.0009	α_{19}	1.4905	0.0004	0.0309	0.0004
$\gamma_{19,1}$	0.1988	0.0005	0.0571	0.0003	$\gamma_{19,3}$	0.0927	0.0020	0.1228	0.0008	α_{20}	1.1457	0.0003	0.0237	0.0002
$\gamma_{20,1}$	0.2193	0.0008	0.0678	0.0004	$\gamma_{20,3}$	0.0420	0.0013	0.1238	0.0007	α_{21}	1.3817	0.0003	0.0280	0.0004
$\gamma_{21,1}$	0.1814	0.0011	0.0684	0.0004	$\gamma_{21,3}$	0.0755	0.0021	0.1228	0.0008	α_{22}	1.1046	0.0003	0.0235	0.0003
$\gamma_{0,2}$	0.4749	0.0017	0.1300	0.0015	$\gamma_{0,4}$	0.4772	0.0037	0.1799	0.0014	β_1	-0.2114	0.0002	0.0113	0.0001
$\gamma_{1,2}$	-0.2607	0.0002	0.0141	0.0001	$\gamma_{1,4}$	-0.1902	0.0004	0.0321	0.0001	β_2	0.1471	0.0001	0.0114	0.0001
$\gamma_{2,2}$	-0.0930	0.0007	0.0360	0.0004	$\gamma_{2,4}$	-0.1798	0.0009	0.0712	0.0007	β_3	-1.2418	0.0002	0.0151	0.0002
$\gamma_{3,2}$	-0.1110	0.0008	0.0279	0.0002	$\gamma_{3,4}$	-0.1931	0.0010	0.0548	0.0003	β_4	1.6993	0.0005	0.0227	0.0005
$\gamma_{4,2}$	-0.0383	0.0004	0.0248	0.0002	$\gamma_{4,4}$	-0.0959	0.0005	0.0488	0.0001	β_5	-0.2978	0.0001	0.0116	0.0001
$\gamma_{5,2}$	-0.2766	0.0007	0.0350	0.0002	$\gamma_{5,4}$	-0.2498	0.0011	0.0624	0.0003	β_6	0.1333	0.0001	0.0112	0.0001
$\gamma_{6,2}$	-0.5288	0.0006	0.0342	0.0002	$\gamma_{6,4}$	-0.4505	0.0004	0.0617	0.0003	β_7	-0.7105	0.0001	0.0131	0.0001
$\gamma_{7,2}$	-0.6437	0.0006	0.0352	0.0002	$\gamma_{7,4}$	-0.5728	0.0003	0.0659	0.0003	β_8	0.3824	0.0002	0.0117	0.0001
$\gamma_{8,2}$	-0.6768	0.0006	0.0428	0.0001	$\gamma_{8,4}$	-0.7139	0.0016	0.0882	0.0008	β_9	-0.3855	0.0001	0.0119	0.0000
$\gamma_{9,2}$	-0.5621	0.0012	0.1393	0.0010	$\gamma_{9,4}$	-0.8352	0.0031	0.4480	0.0031	β_{10}	0.0763	0.0001	0.0116	0.0001
$\gamma_{10,2}$	0.0006	0.0002	0.0176	0.0001	$\gamma_{10,4}$	-0.0081	0.0021	0.0518	0.0004	β_{11}	0.6393	0.0002	0.0123	0.0001
$\gamma_{11,2}$	0.0054	0.0005	0.0160	0.0001	$\gamma_{11,4}$	-0.0484	0.0006	0.0366	0.0003	β_{12}	0.2883	0.0002	0.0117	0.0001
$\gamma_{12,2}$	-0.0927	0.0015	0.0817	0.0006	$\gamma_{12,4}$	0.0878	0.0029	0.1607	0.0015	β_{13}	-0.3463	0.0001	0.0112	0.0001
$\gamma_{13,2}$	0.0524	0.0005	0.0297	0.0002	$\gamma_{13,4}$	-0.0496	0.0010	0.0616	0.0001	β_{14}	0.8661	0.0002	0.0134	0.0001
$\gamma_{14,2}$	-0.0021	0.0015	0.1304	0.0009	$\gamma_{14,4}$	-0.0377	0.0012	0.1640	0.0012	β_{15}	-1.1036	0.0002	0.0146	0.0001
$\gamma_{15,2}$	0.0781	0.0014	0.1222	0.0010	$\gamma_{15,4}$	-0.0082	0.0014	0.1586	0.0017	β_{16}	-1.4041	0.0003	0.0164	0.0002
$\gamma_{16,2}$	0.1349	0.0012	0.1244	0.0009	$\gamma_{16,4}$	0.0918	0.0018	0.1653	0.0015	β_{17}	0.8853	0.0004	0.0169	0.0001
$\gamma_{17,2}$	0.1022	0.0014	0.1211	0.0010	$\gamma_{17,4}$	0.0171	0.0012	0.1543	0.0018	β_{18}	0.3316	0.0001	0.0118	0.0000
$\gamma_{18,2}$	0.1506	0.0015	0.1255	0.0008	$\gamma_{18,4}$	0.0633	0.0024	0.1629	0.0015	β_{19}	-0.2808	0.0004	0.0125	0.0000
$\gamma_{19,2}$	0.1251	0.0015	0.1221	0.0009	$\gamma_{19,4}$	0.0890	0.0019	0.1584	0.0016	β_{20}	0.3172	0.0001	0.0123	0.0001
$\gamma_{20,2}$	0.1980	0.0013	0.1242	0.0012	$\gamma_{20,4}$	0.0955	0.0020	0.1640	0.0014	β_{21}	-0.0032	0.0001	0.0121	0.0001
$\gamma_{21,2}$	0.1872	0.0015	0.1238	0.0012	$\gamma_{21,4}$	0.1779	0.0020	0.1591	0.0015	β_{22}	0.2183	0.0001	0.0119	0.0001
σ^2_1	0.0872	0.0001	0.0042	0.0000	υ^2_1	0.0443	0.0001	0.0055	0.0000	$\kappa_{3,2}$	0.6326	0.0002	0.0134	0.0001
σ^2_2	0.1078	0.0001	0.0043	0.0000	υ^2_2	0.0595	0.0000	0.0077	0.0000	$\kappa_{3,3}$	1.8212	0.0002	0.0184	0.0002
σ^2_3	0.1509	0.0001	0.0052	0.0000	υ^2_3	0.0810	0.0001	0.0100	0.0000	$\kappa_{16,2}$	1.2457	0.0003	0.0172	0.0002
σ^2_4	0.1217	0.0001	0.0102	0.0001	υ^2_4	0.0898	0.0003	0.0236	0.0002	$\kappa_{16,3}$	1.8569	0.0005	0.0189	0.0003

Notes: $m(\hat{\psi})$ reports the arithmetic mean of the parameter estimates from the five different chains, $sd(\hat{\psi})$ denotes the corresponding standard deviation, $m(\hat{\sigma}_\psi)$ denotes the arithmetic mean of the parameter specific standard deviation from the different chains, $sd(\hat{\sigma}_\psi)$ the corresponding standard deviation.

Figure 1: Simulation study (BD) - Trace plots and ACF plots

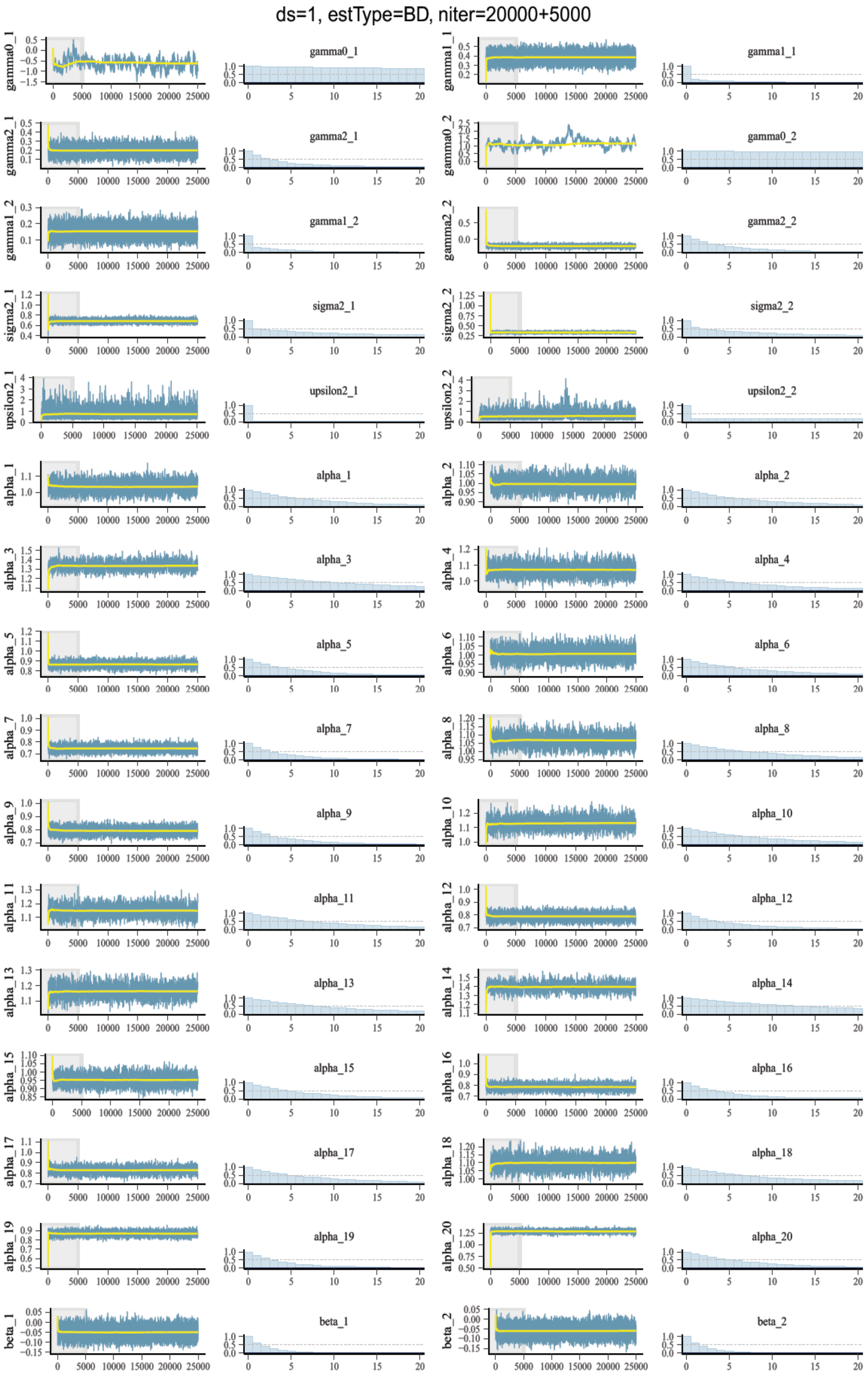

Figure 2: Simulation study (BD) - Trace plots and ACF plots

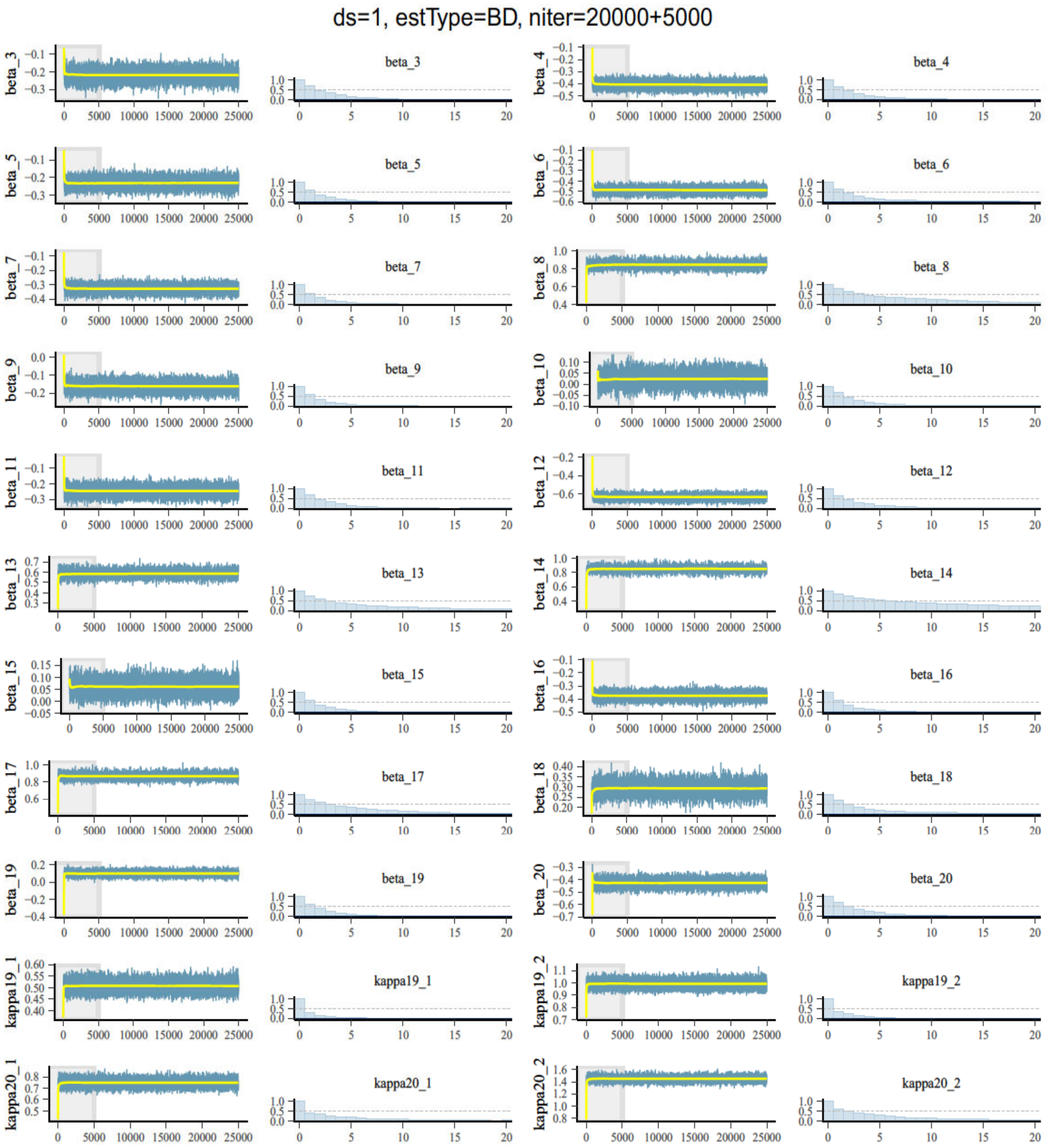

Figure 3: Simulation study (DART-m, Scenario 1) - Trace plots and ACF plots

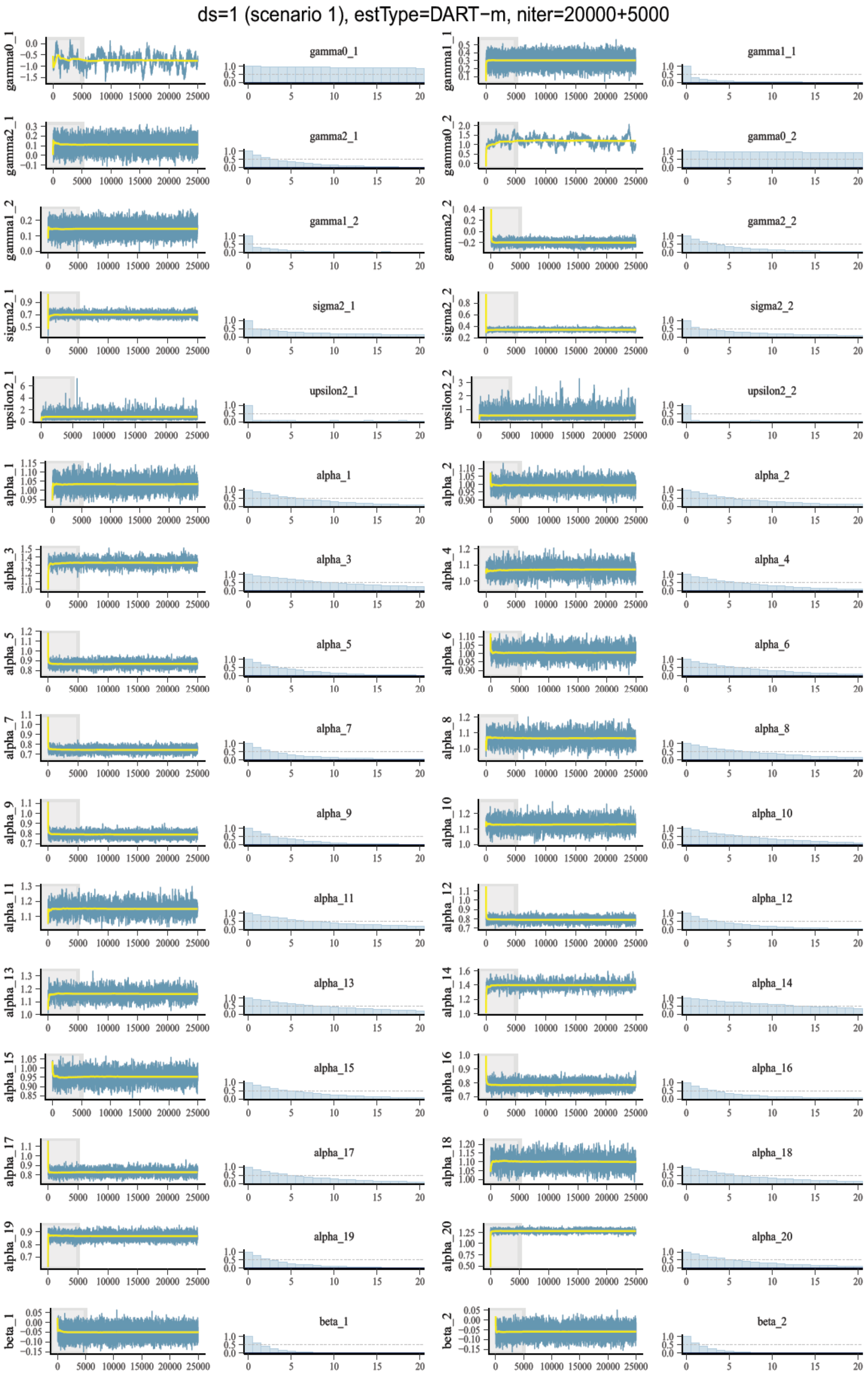

Figure 4: Simulation study (DART-m, Scenario 1) - Trace plots and ACF plots

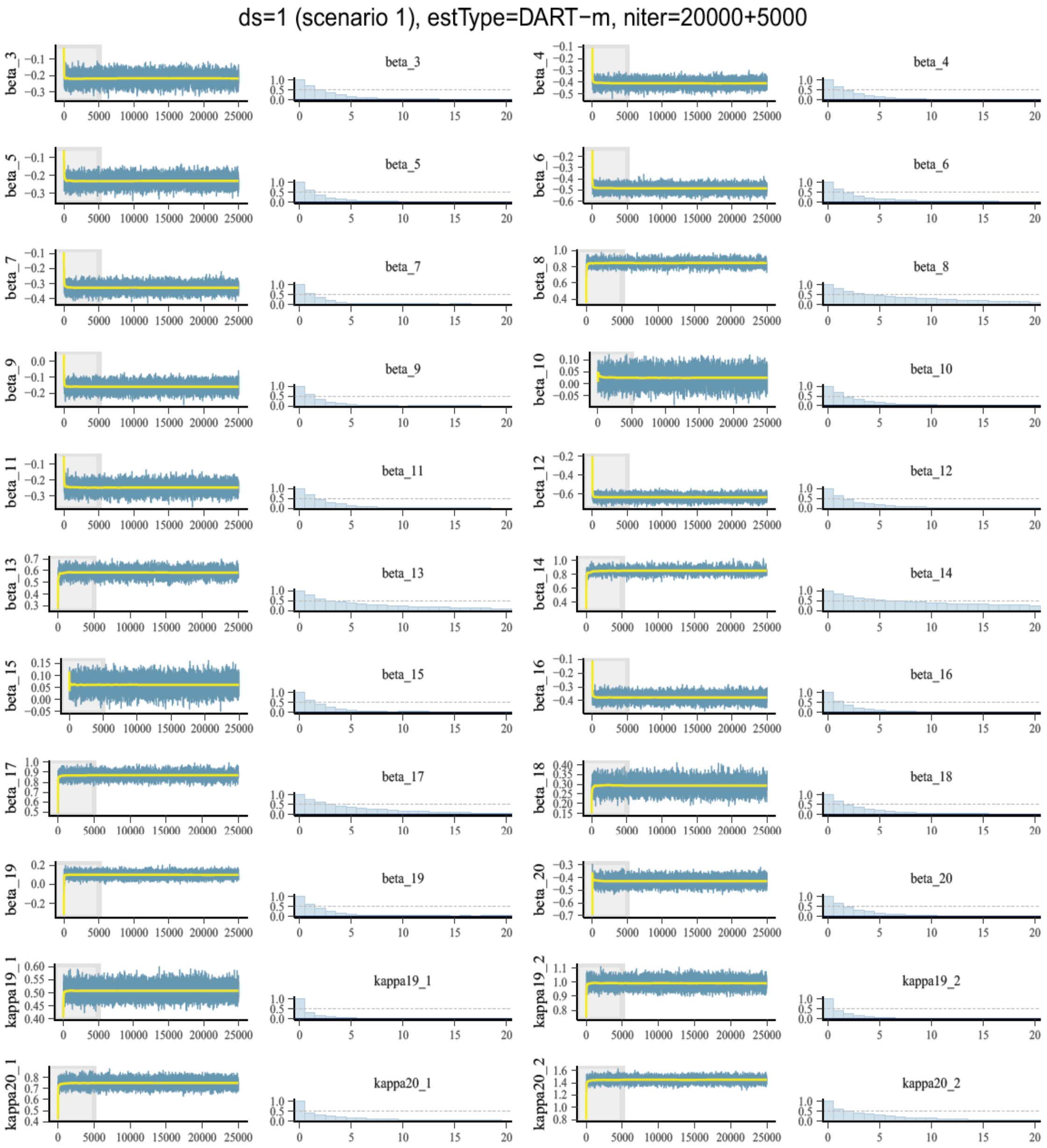

Figure 5: Simulation study (DART-m, Scenario 2) - Trace plots and ACF plots

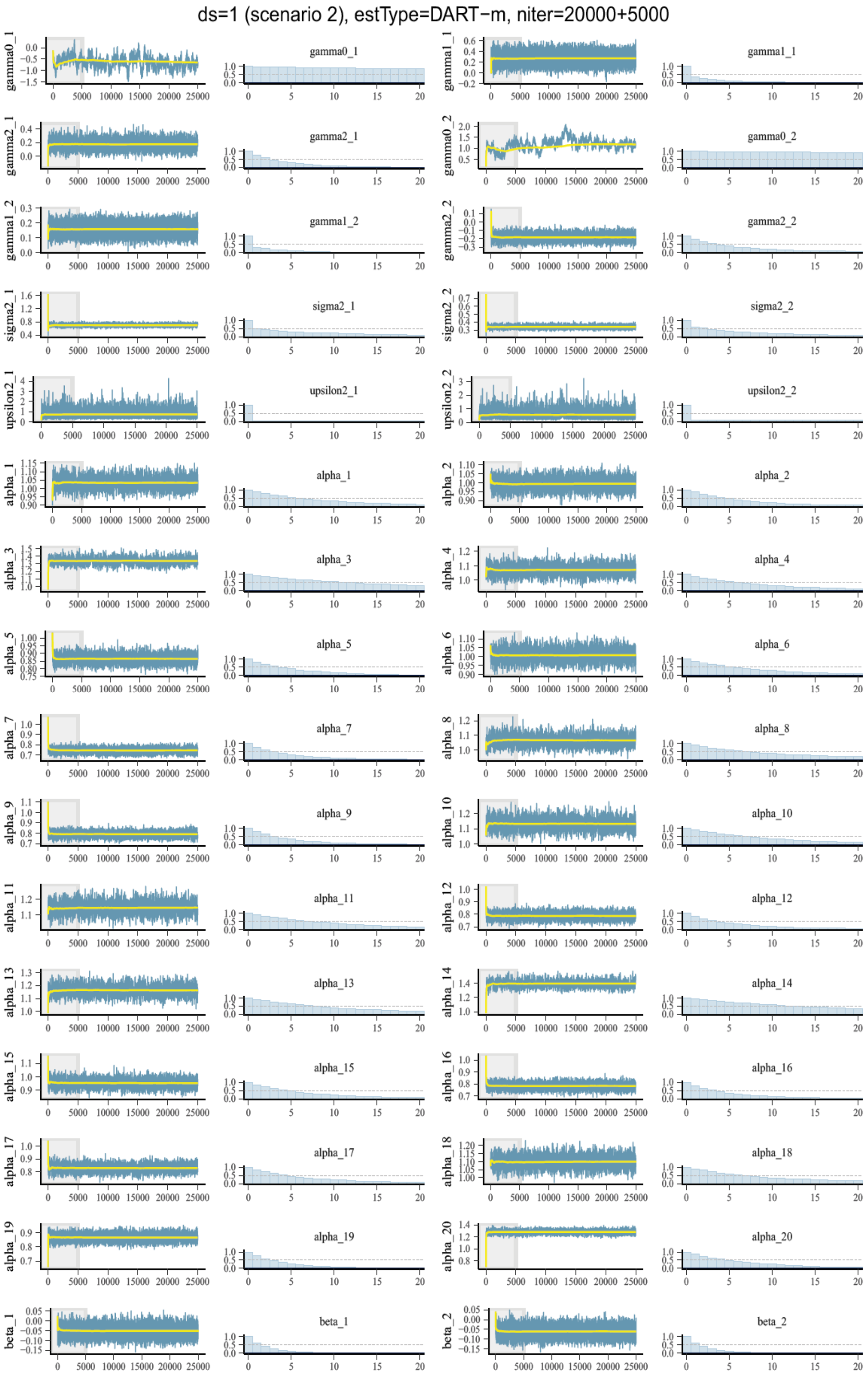

Figure 6: Simulation study (DART-m, Scenario 2) - Trace plots and ACF plots

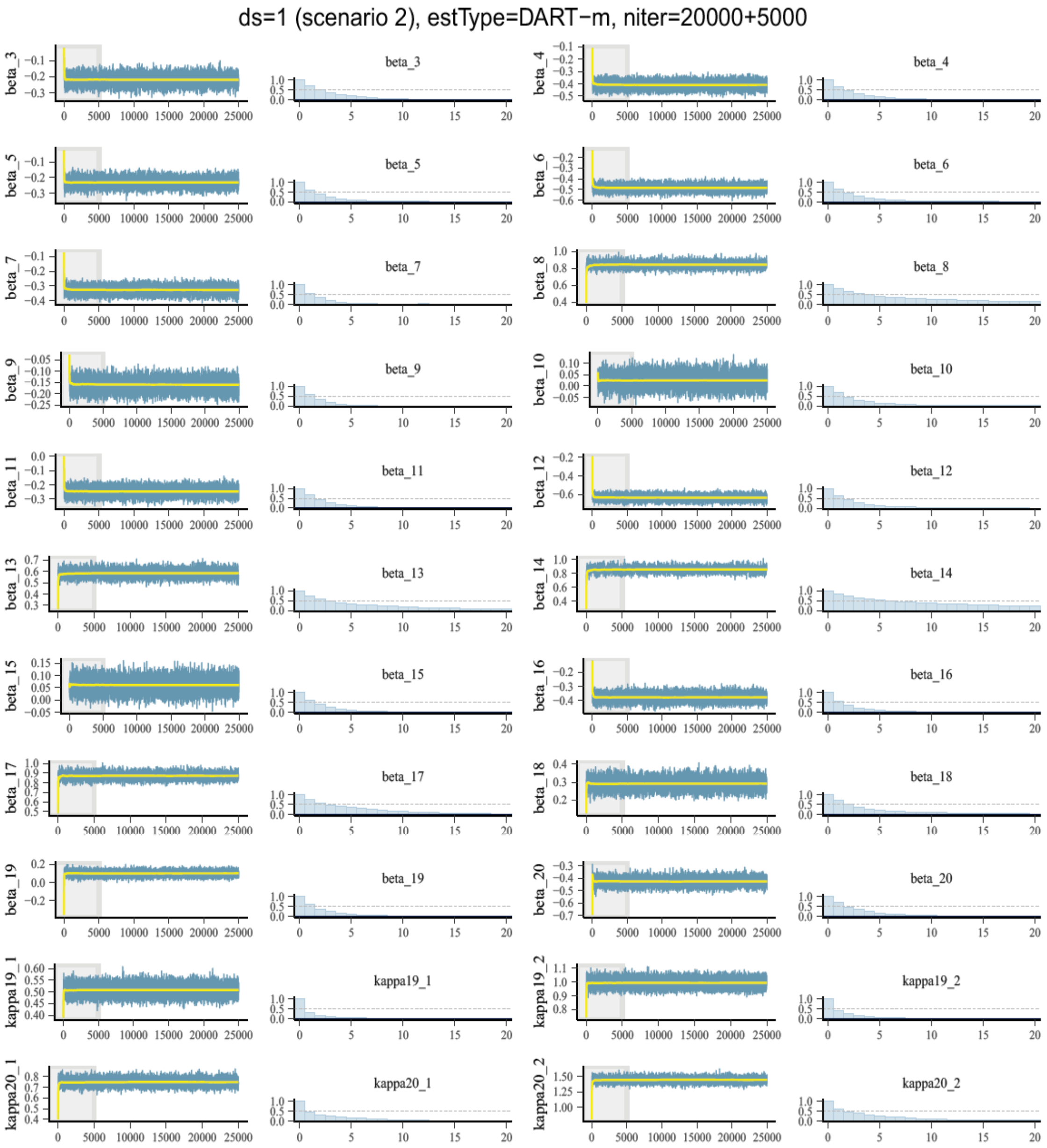

Figure 7: Simulation study (DART-m, Scenario 3) - Trace plots and ACF plots

ds=1 (scenario 3), estType=DART−m, niter=20000+5000

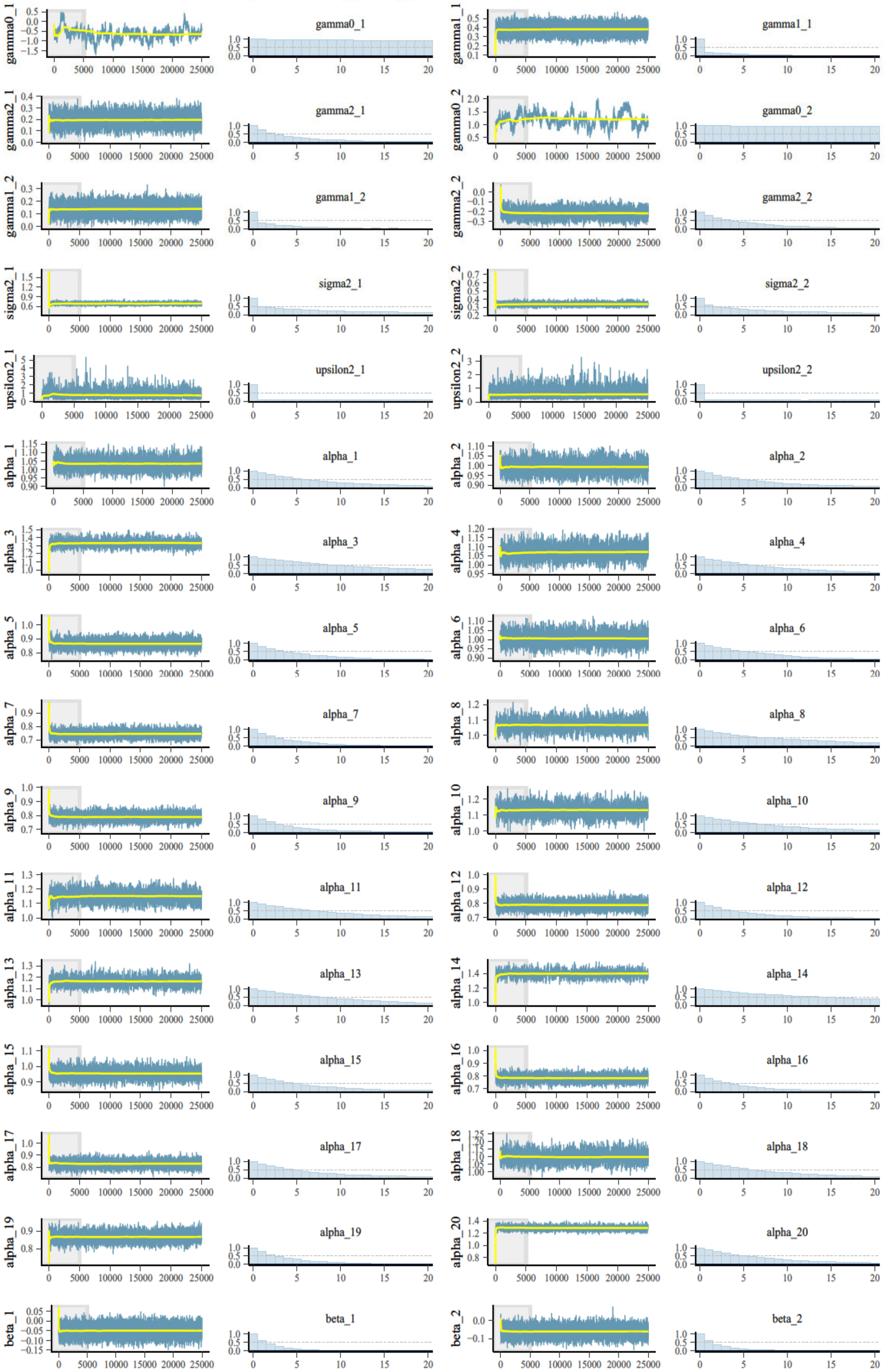

Figure 8: Simulation study (DART-m, Scenario 3) - Trace plots and ACF plots

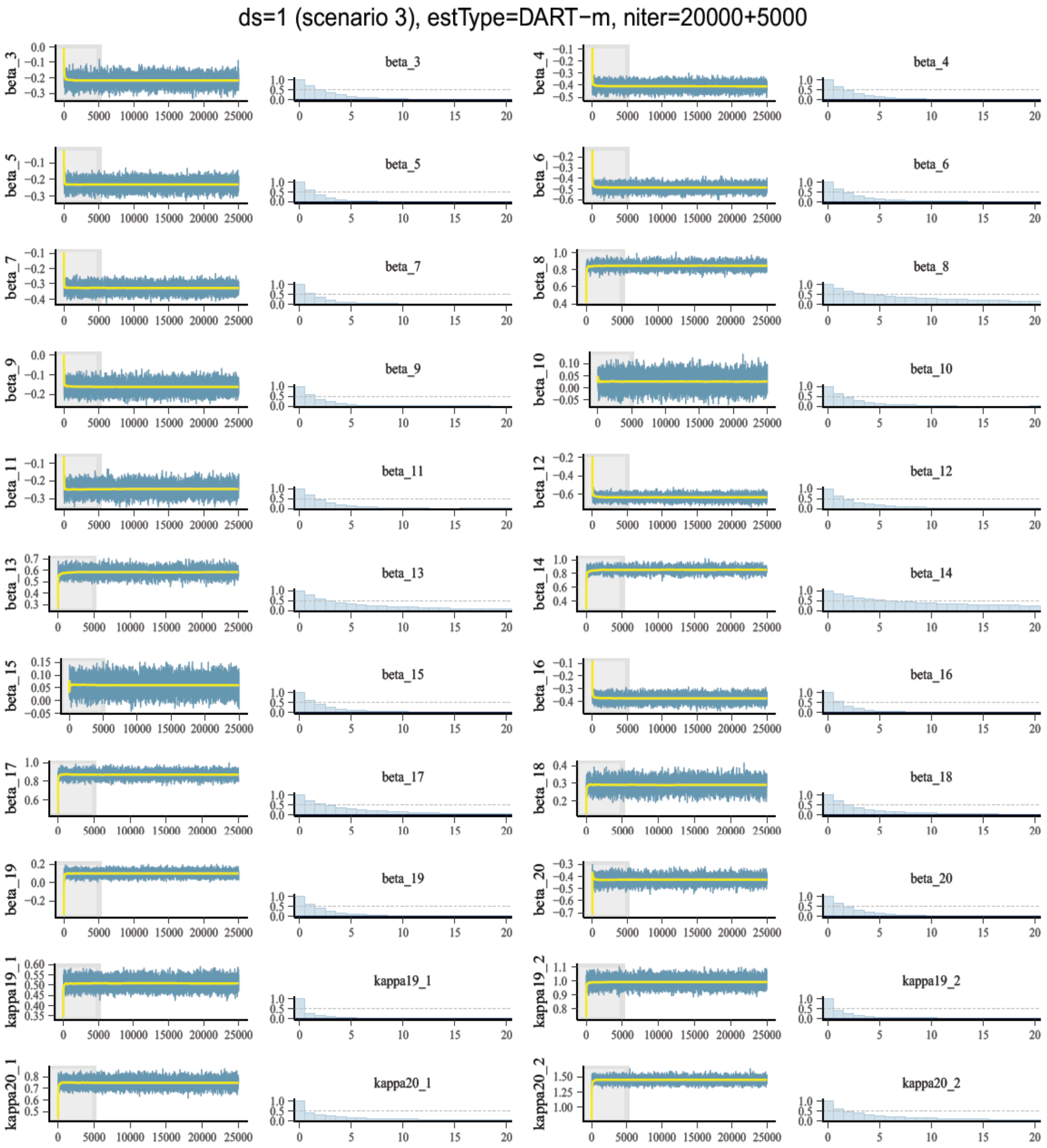

Figure 9: Simulation study (DART-m, Scenario 4) - Trace plots and ACF plots

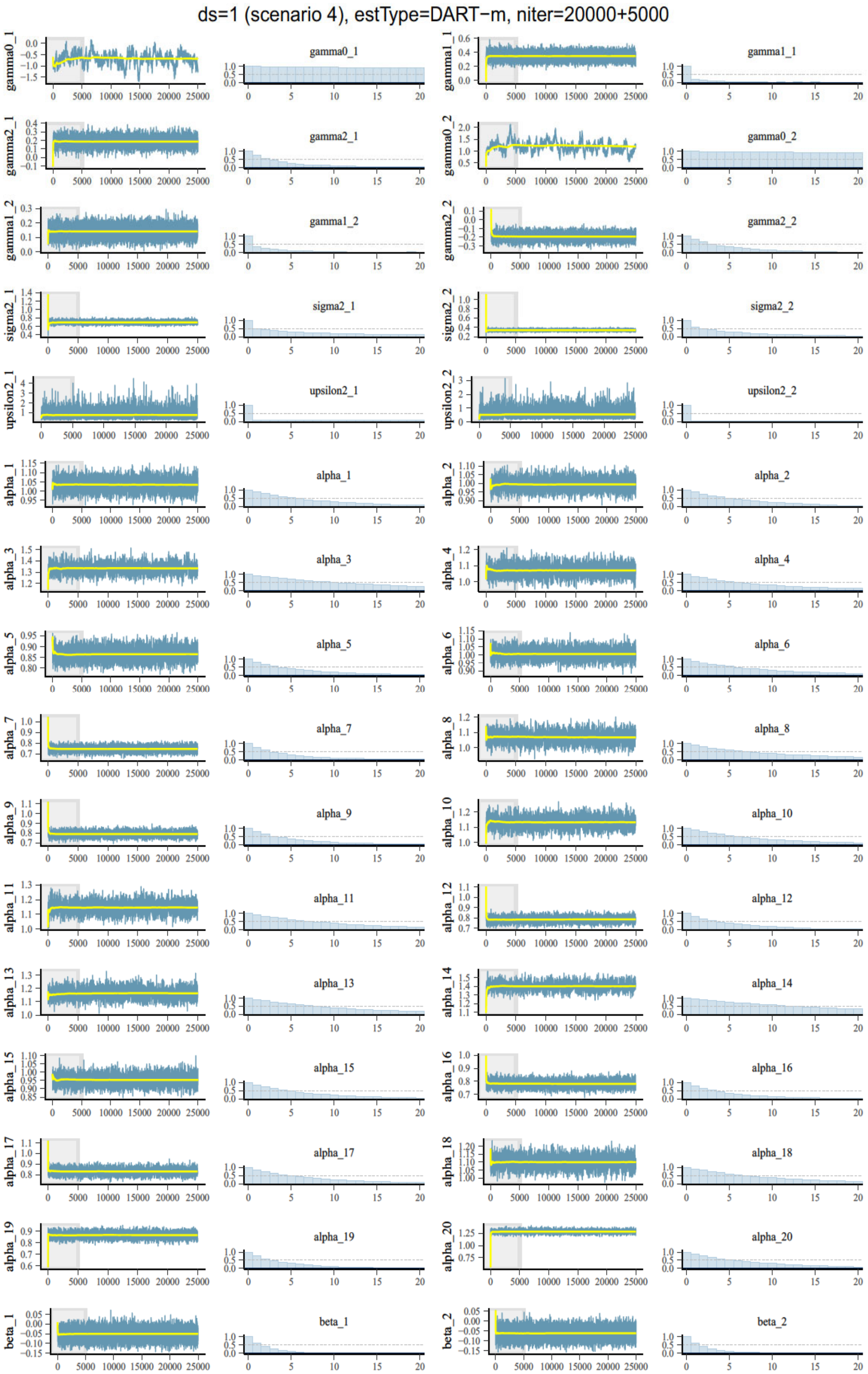

Figure 10: Simulation study (DART-m, Scenario 4) - Trace plots and ACF plots

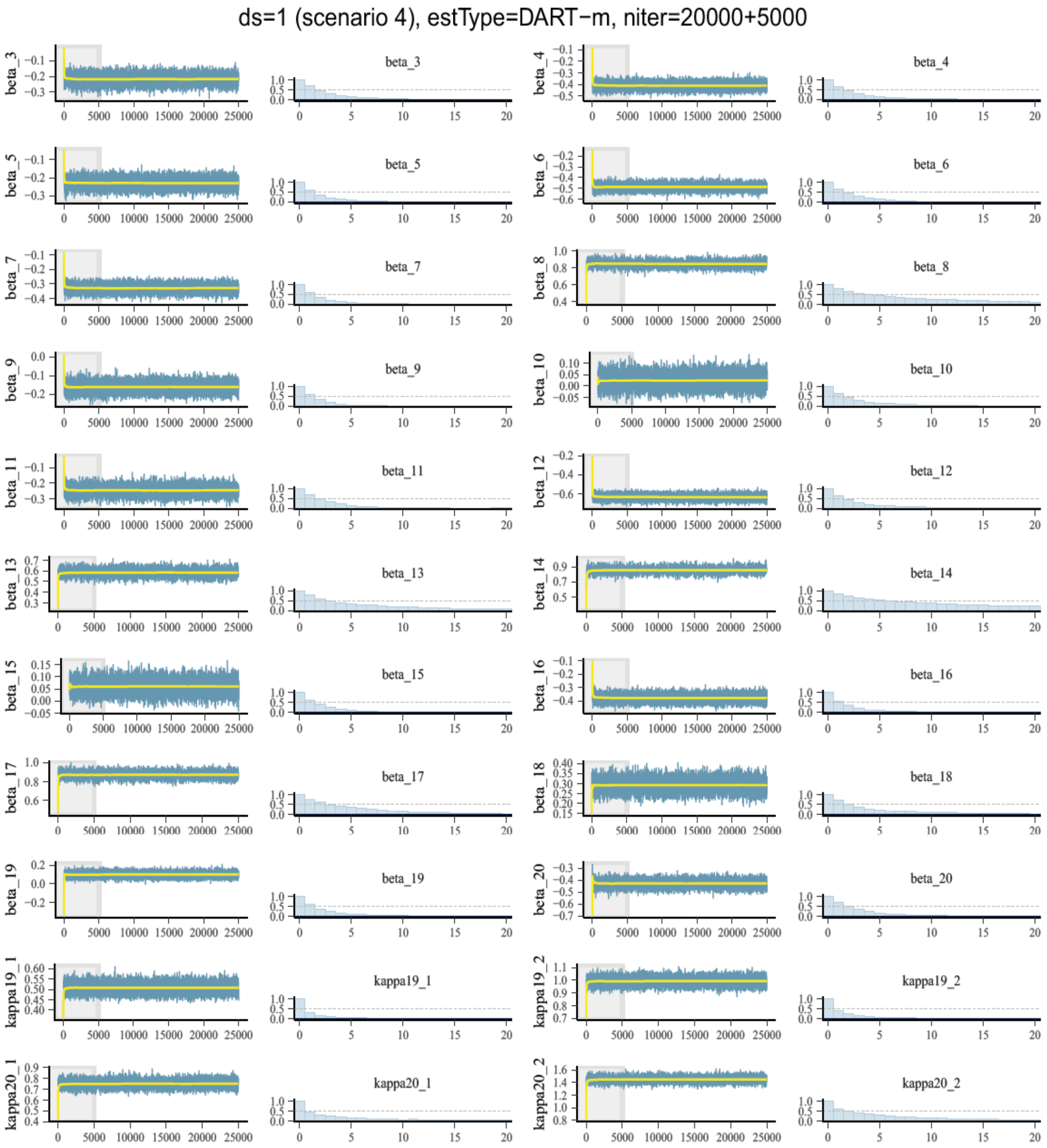

Figure 11: NEPS GRADE 9, MATHEMATICAL COMPETENCIES (model I) - Trace plots and ACF plots

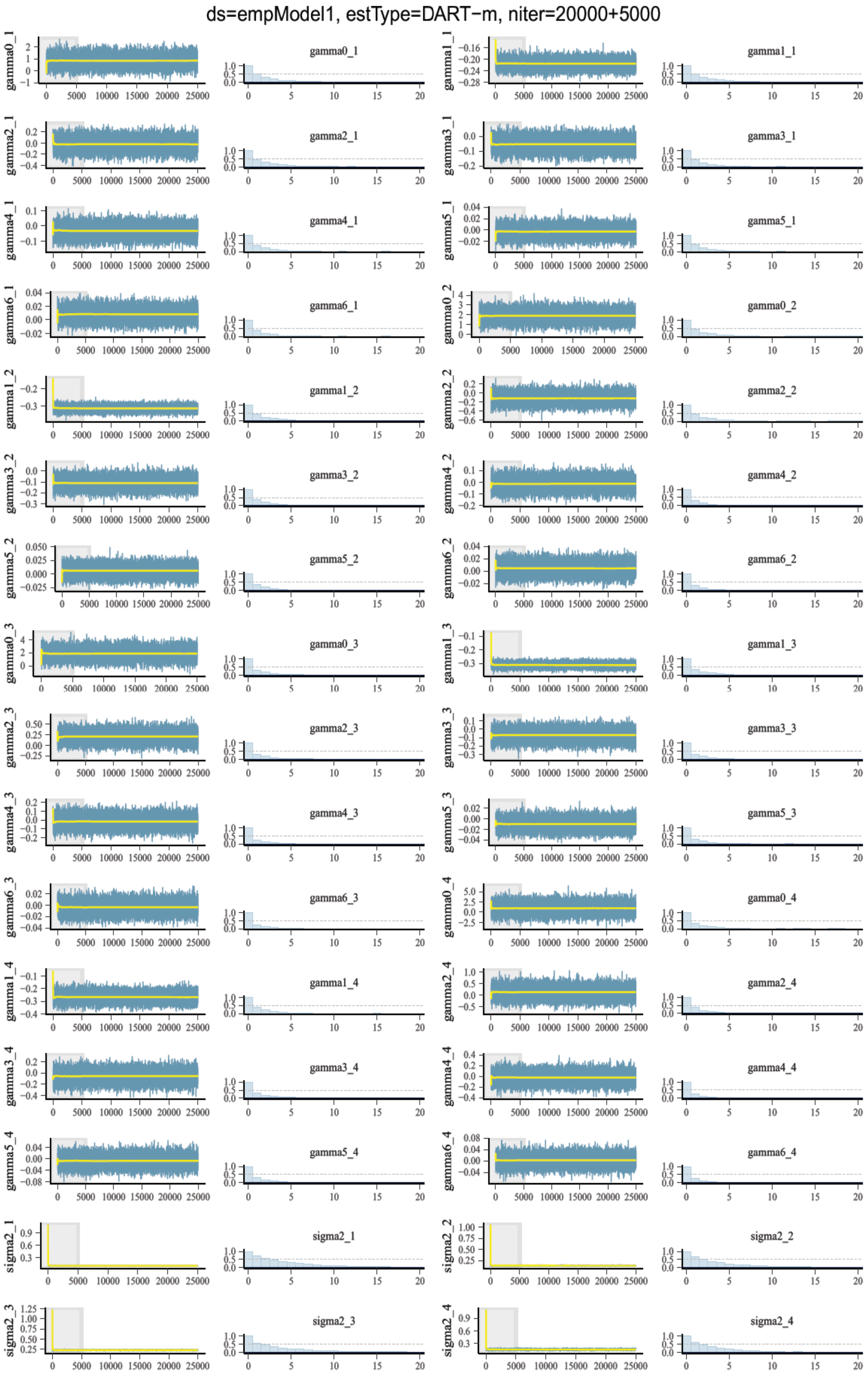

Figure 12: NEPS GRADE 9, MATHEMATICAL COMPETENCIES (model I) - Trace plots and ACF plots

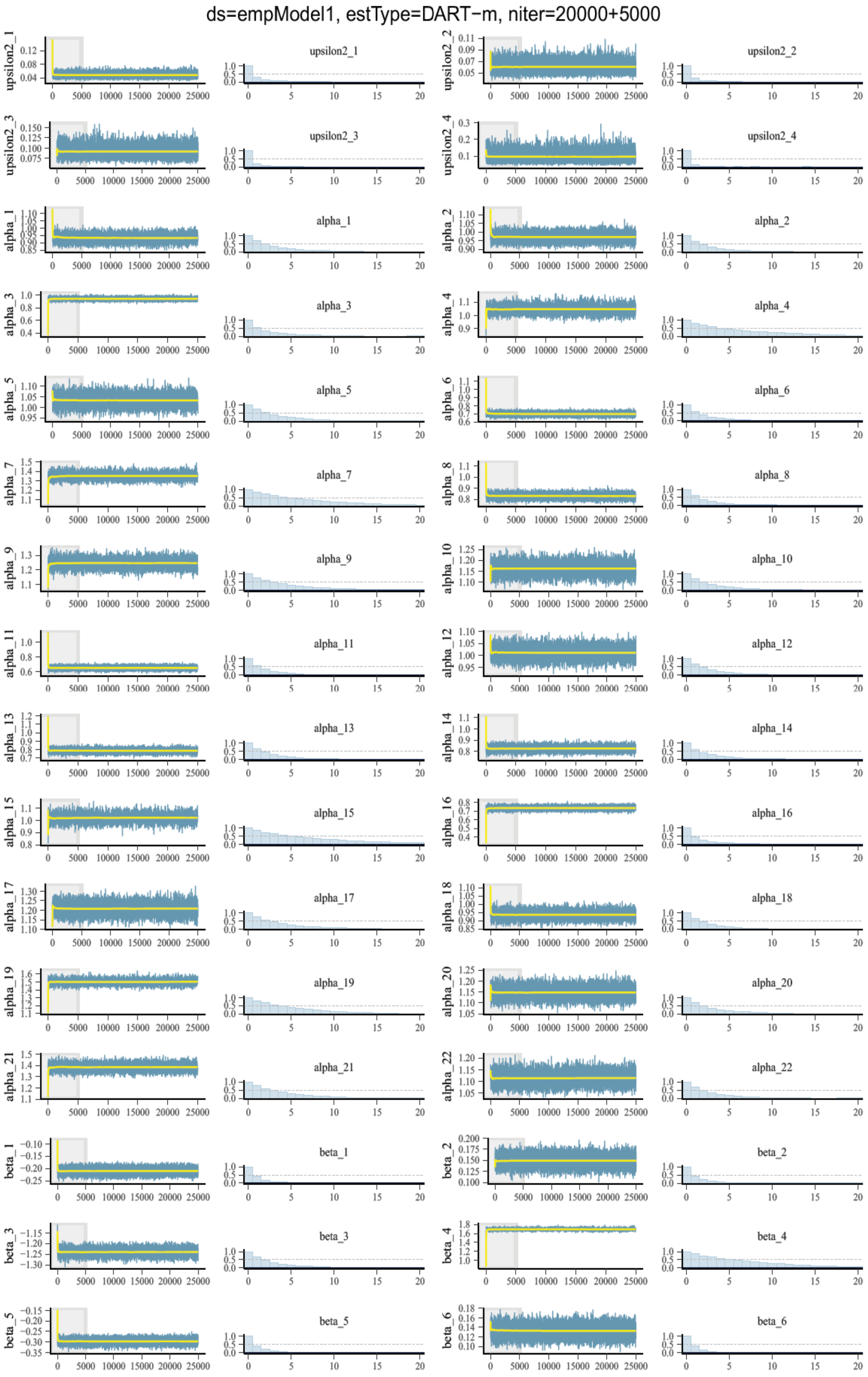

Figure 13: NEPS GRADE 9, MATHEMATICAL COMPETENCIES (model I) - Trace plots and ACF plots

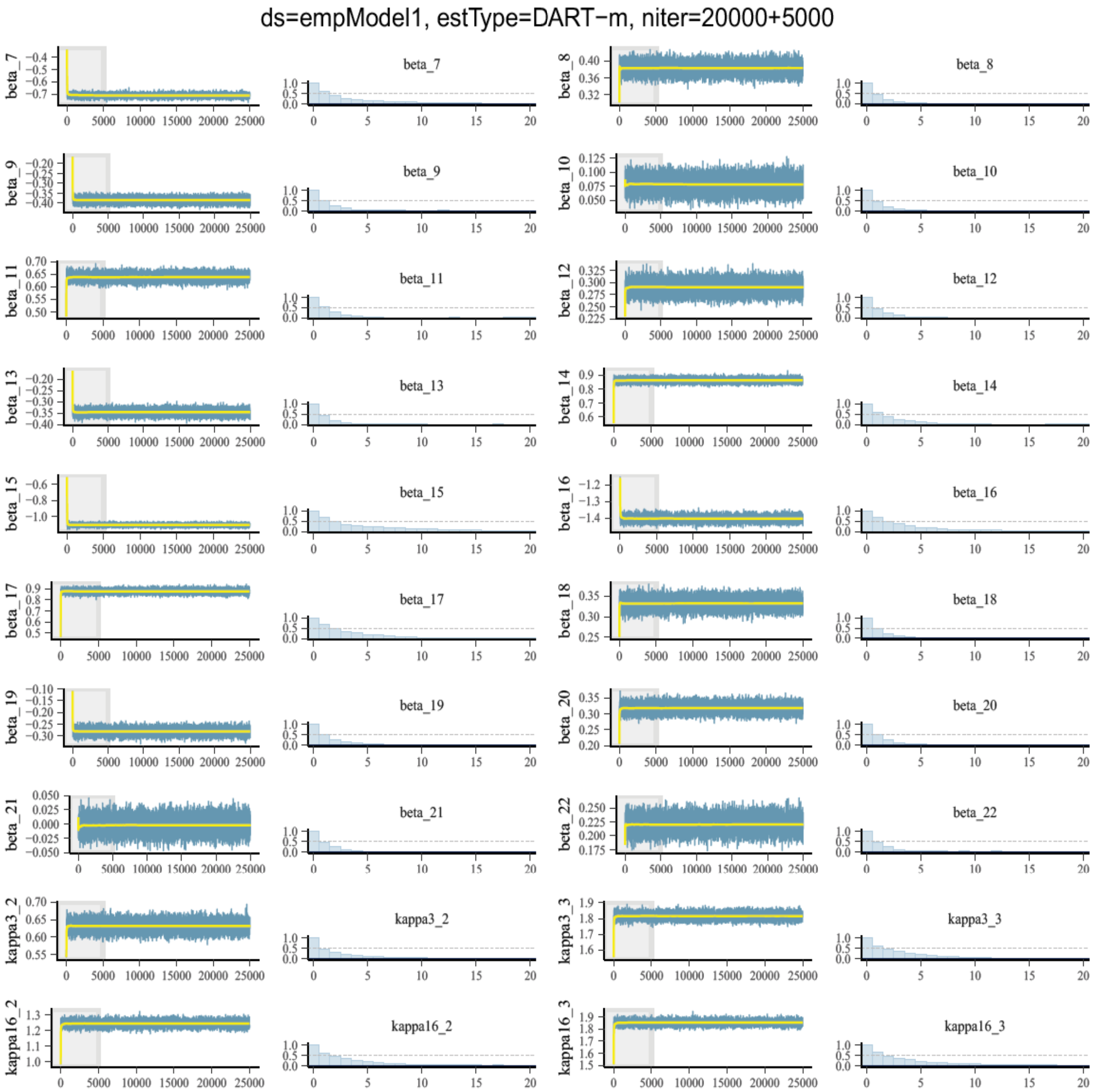

Figure 14: NEPS GRADE 9, MATHEMATICAL COMPETENCIES (model *II*) - Trace plots and ACF plots

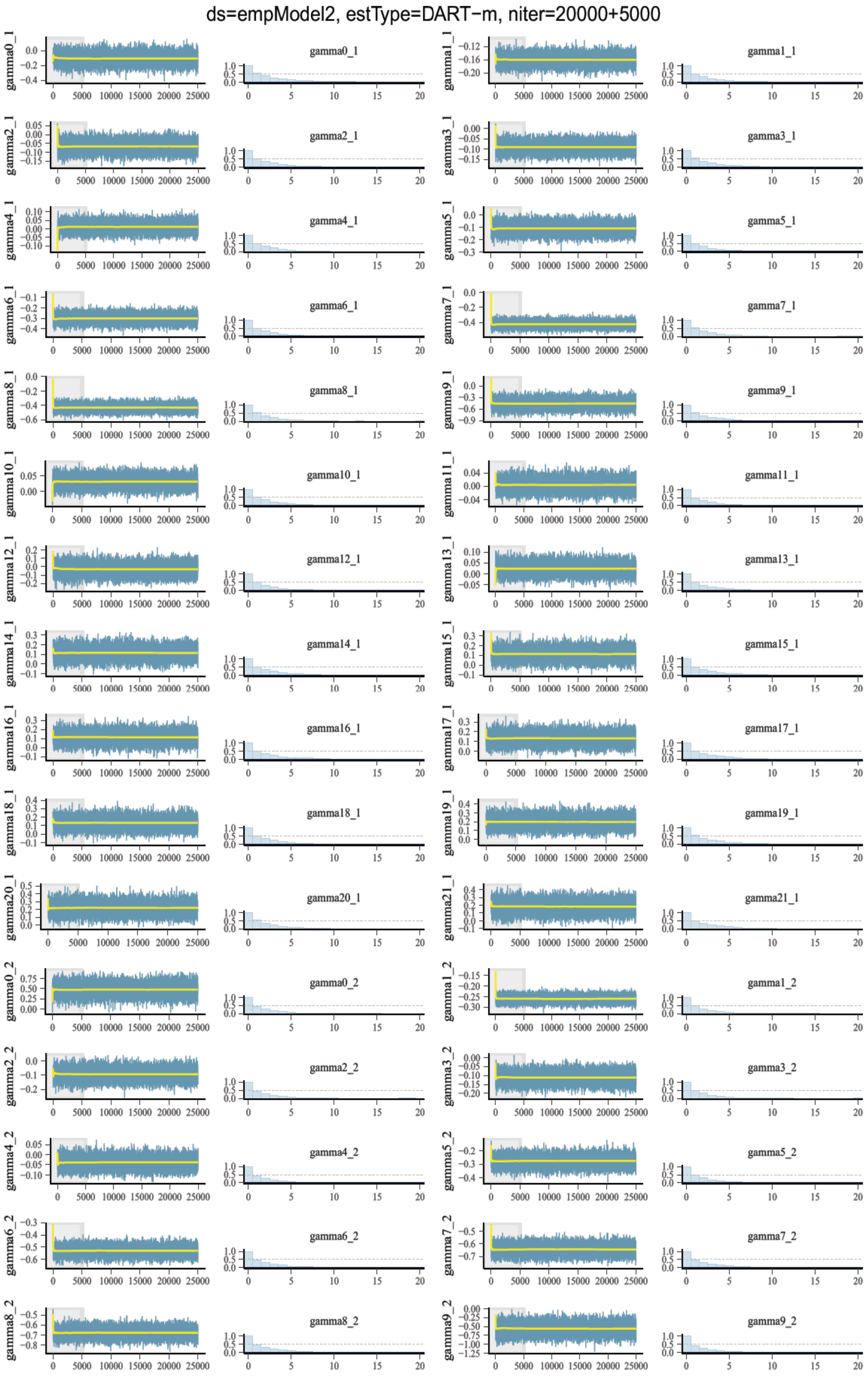

Figure 15: NEPS grade 9, mathematical competencies (model II) - Trace plots and ACF plots

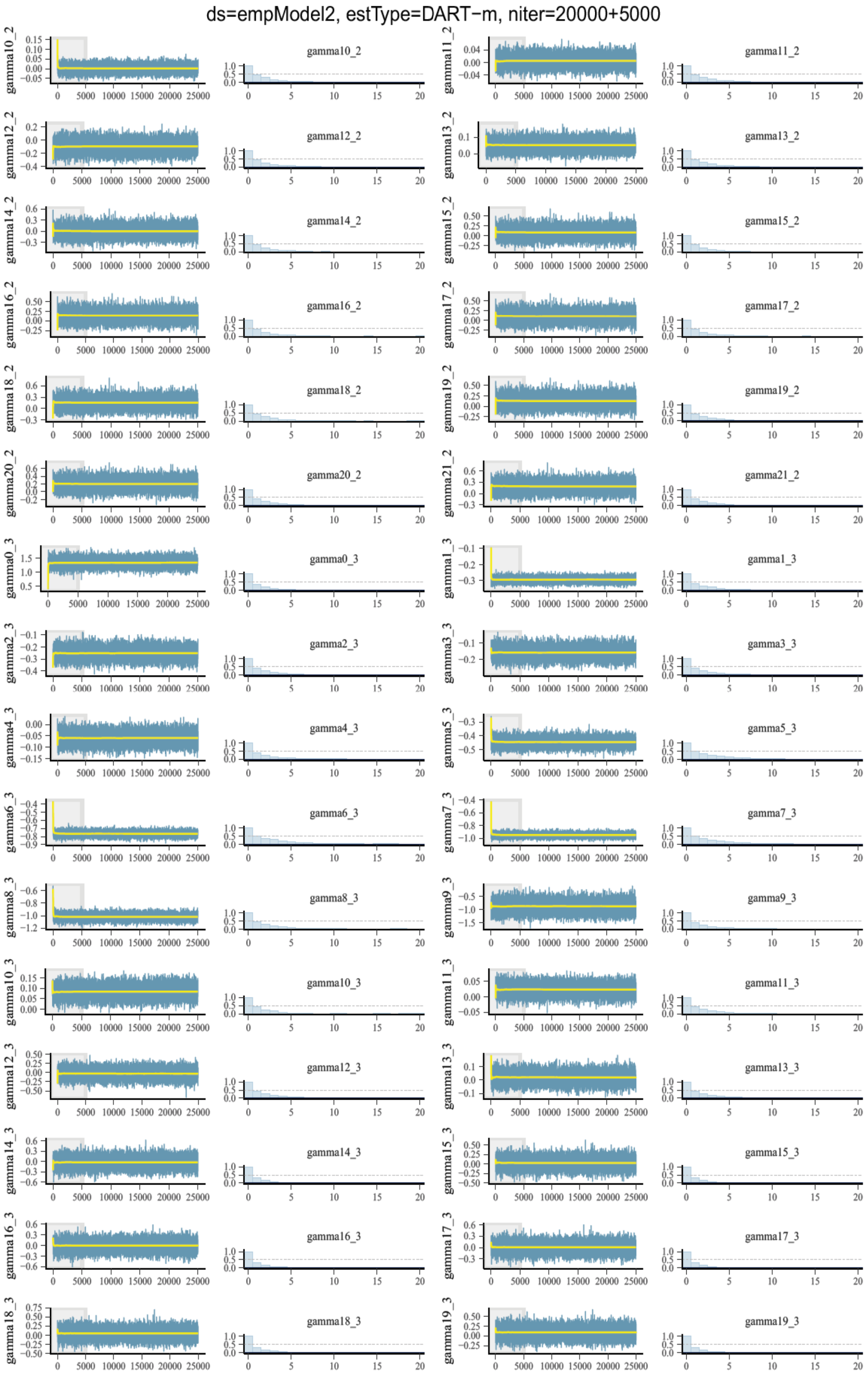

Figure 16: NEPS GRADE 9, MATHEMATICAL COMPETENCIES (model *II*) - Trace plots and ACF plots

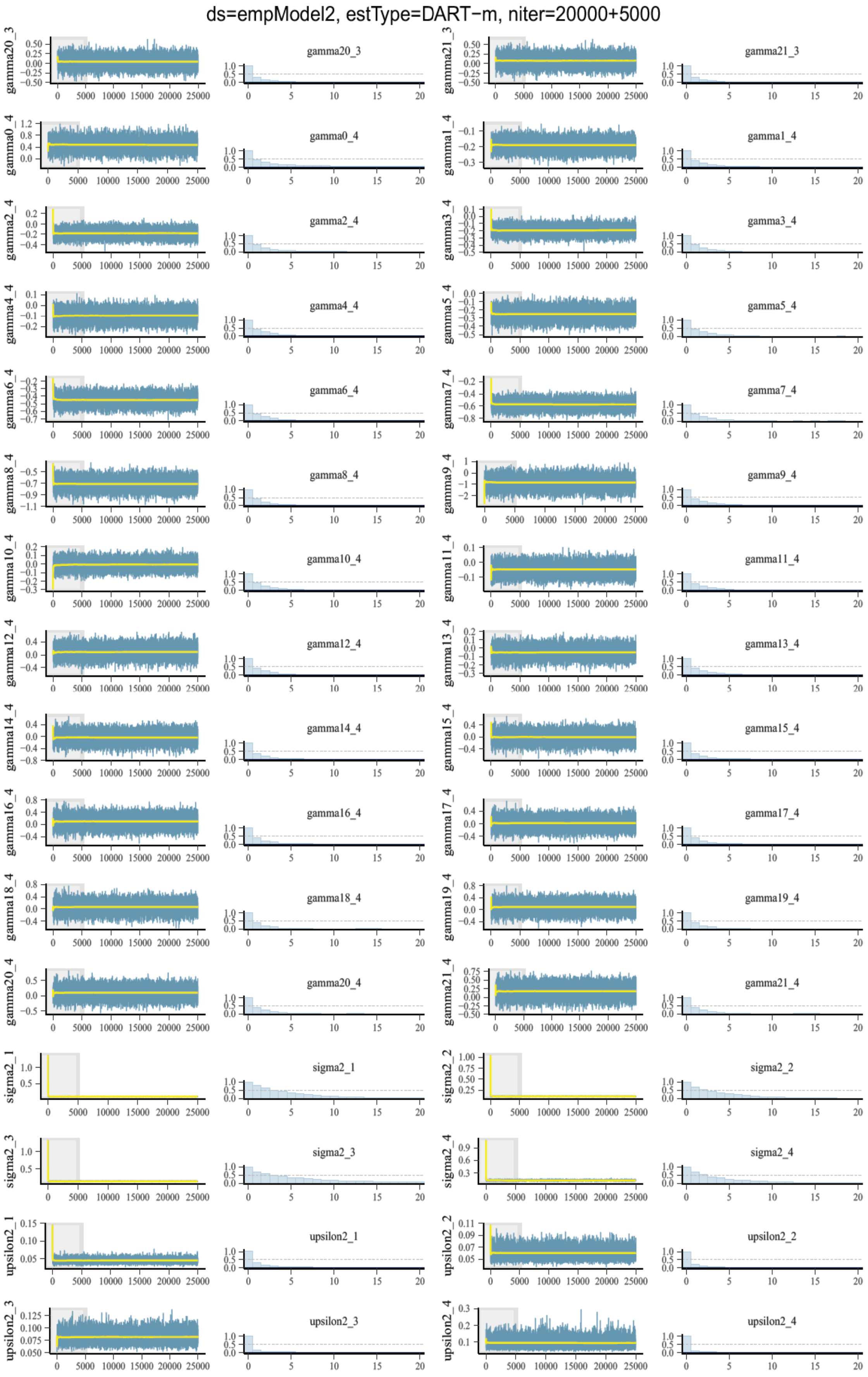

Figure 17: NEPS grade 9, mathematical competencies (model *II*) - Trace plots and ACF plots

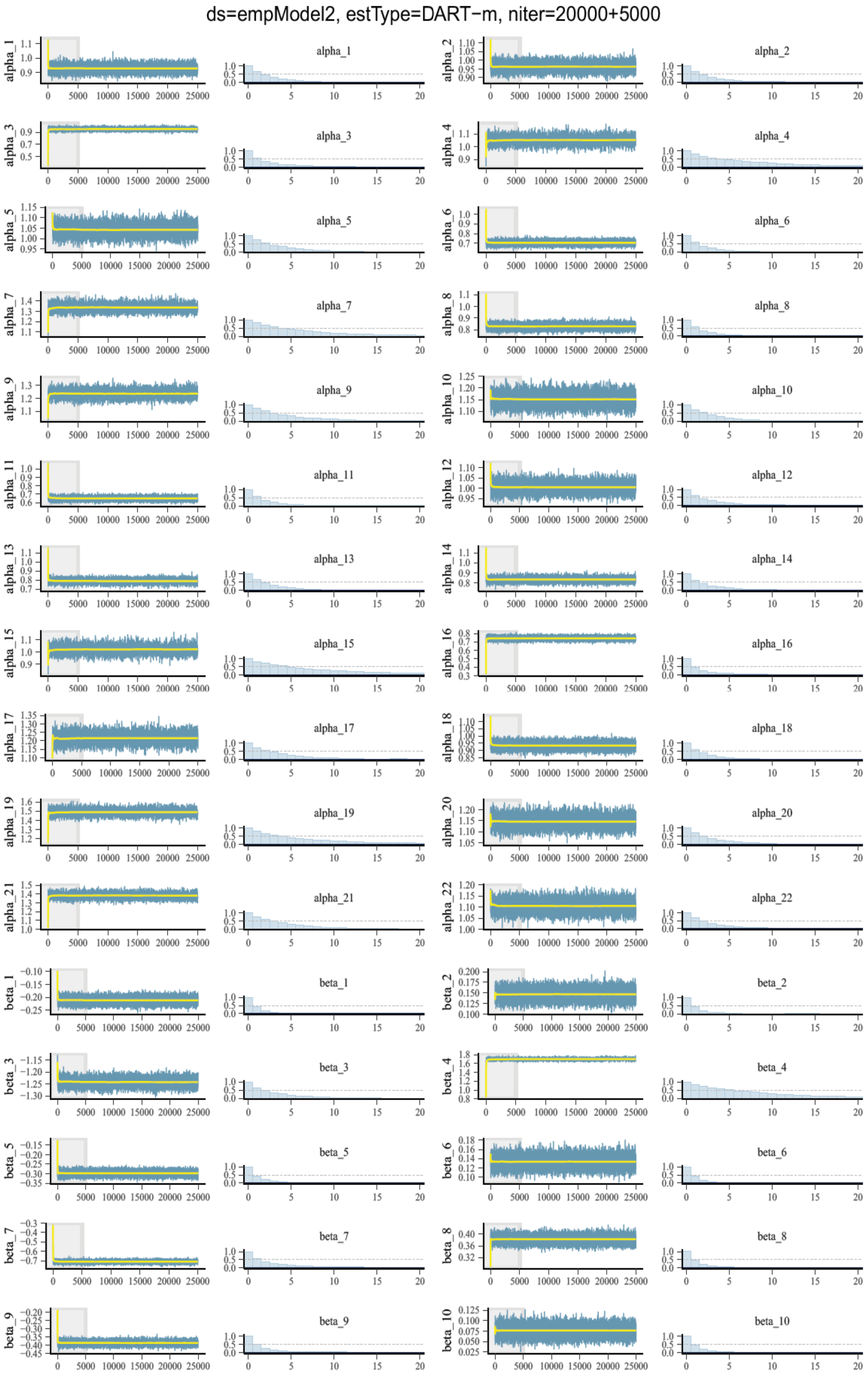

Figure 18: NEPS GRADE 9, MATHEMATICAL COMPETENCIES (model *II*) - Trace plots and ACF plots

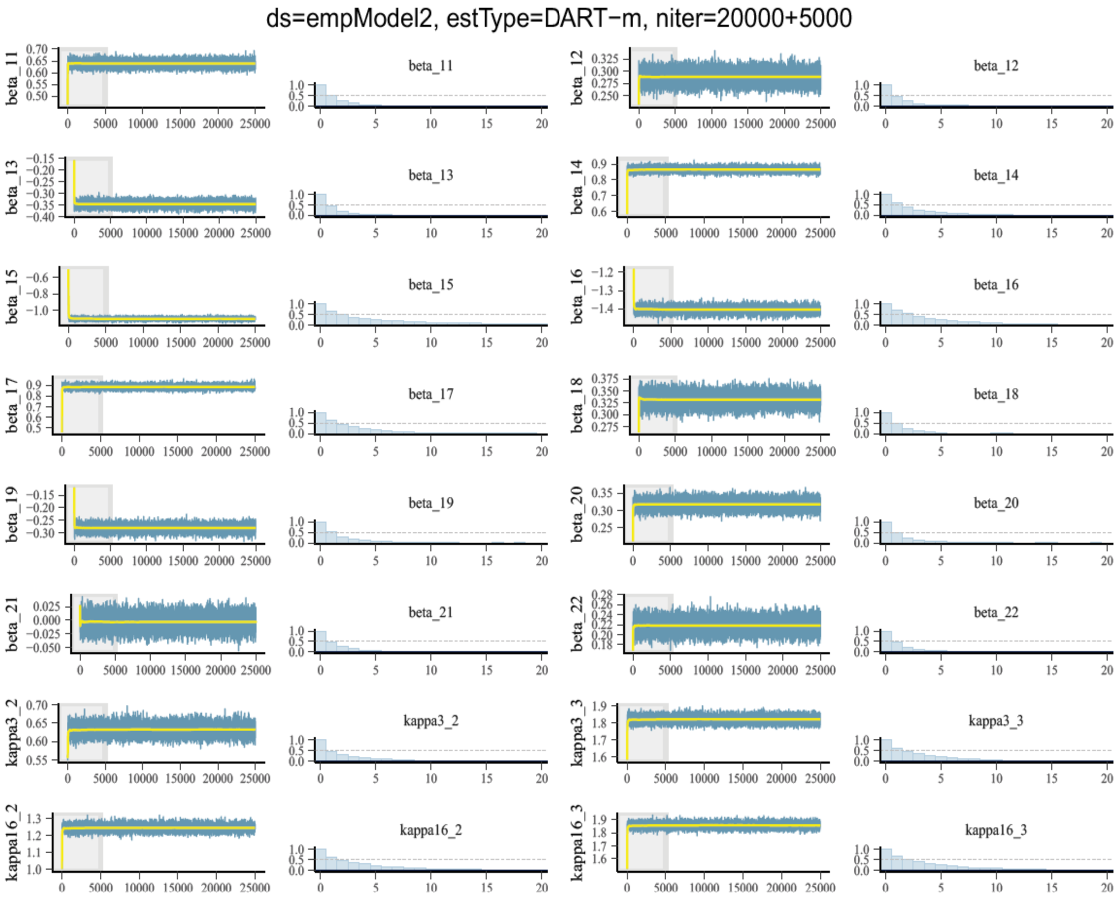

Supplement: Supplementary file 1 — (pdf 2994 KB) [file 11336_2022_9888_MOESM1_ESM.pdf]
